# A mixed-methods study on impact of silicosis on tuberculosis treatment outcomes and need for TB-silicosis collaborative activities in India

**DOI:** 10.1038/s41598-023-30012-4

**Published:** 2023-02-16

**Authors:** Mihir P. Rupani

**Affiliations:** Clinical Epidemiology (Division of Health Sciences), ICMR - National Institute of Occupational Health (NIOH), Indian Council of Medical Research, Meghaninagar, Ahmedabad City, Gujarat 380016 India

**Keywords:** Tuberculosis, Population screening, Occupational health, Health policy

## Abstract

Globally, silicosis and tuberculosis (TB) have been targeted for elimination by 2030. The study’s objectives were to determine the association of silicosis with unfavorable TB treatment outcomes, as well as to explore experts’ perspectives on improving treatment outcomes among silico-tuberculosis patients. A retrospective cohort study evaluated TB treatment outcomes in Khambhat block, the western part of India, between 138 patients with silico-tuberculosis and 2610 TB patients without silicosis in February–March 2022. ‘Unfavorable TB treatment outcomes’ was defined as a patient stopping treatment for at least one month, a positive sputum smear at the end of treatment, or, a patient dying while on treatment. During April–July 2022, fifteen in-depth interviews with experts in the field of silicosis/tuberculosis were audio-recorded, transcribed, and analyzed to generate codes (thematic analysis). On multivariable logistic regression analysis, patients with silico-tuberculosis had a 2.3 (95% CI 1.6–3.4) times higher odds of unfavorable treatment outcomes. The experts recommended collaborative TB-silicosis activities for improving treatment outcomes of patients with silico-tuberculosis. I conclude from the study’s findings that silicosis is associated with unfavorable TB treatment outcomes in this study setting. All patients with silicosis should be screened for TB and treated according to national TB program guidelines. All patients with TB who have a history of occupational dust exposure should be evaluated for silicosis and provided appropriate pulmonary/vocational rehabilitation.

## Introduction

Silicosis, a fibrotic lung disease caused by inhaling crystalline silica dust, is common among workers in a variety of occupations worldwide^1^. Stone quarrying, mining, building construction, foundries, and glass production are some of these vocations^1^. In 2019, 2.65 million prevalent cases of silicosis were reported globally^2^. Nearly 227 million workers are at risk of developing silicosis, with the vast majority in the unorganized sector and being migratory^3–5^. With the introduction of informal contracts and other types of casual labor in bigger companies across India, over 92% of workers are working in informal employment, which means they do not have access to social security benefits^6,7^. Over 12.9 thousand deaths occurred due to silicosis worldwide, with an estimated 0.65 million disability-adjusted life years (DALYs) in 2019^8^. Between 1990 and 2019, the number of fatalities and DALYs attributable to silicosis decreased overall but increased in low- and middle-income countries^8^.

Tuberculosis (TB) is one of the most prevalent diseases among patients with silicosis^9^. Patients with silicosis are three to four times more likely to get TB than those who do not have silicosis^9,10^. The average time between the termination of silica dust exposure and diagnosis of pulmonary TB has been reported as eight years^11^. Patients with both silicosis and TB are given the combined diagnosis of silico-tuberculosis, the true burden of which is largely unknown due to a lack of surveillance and insufficient healthcare access^3,12,13^.

Several mechanisms have been implicated in silica dust impeding successful treatment completion and increasing the odds of relapse among patients with TB^14^. Exposure to crystalline silica dust triggers oxidative-nitrosative stress, immunological dysfunction, macrophage impairment, and poor drug penetration, allowing the TB bacilli to survive in the lungs’ alveoli^15–18^. Patients with silico-tuberculosis have a higher risk of relapse and more treatment interruptions^19,20^.

India has the highest number of TB cases in the world^21^. The global End-TB strategy aims to achieve ≥ 90% treatment success rates among patients with TB by the year 2025^22^. Under programmatic settings of TB, the risk of failure to complete treatment among patients with silico-tuberculosis is not yet known. Globally, both silicosis, as well as tuberculosis, are targeted for elimination by the year 2030^22–24^. The need of integrating silicosis control efforts with TB control programs has been widely emphasized^3,12^. However, the implementation mechanisms of such an integrated TB-silicosis program are unexplored. The objectives of this study were to determine the association of silicosis with unfavorable TB treatment outcomes and to explore experts’ perspectives on improving TB treatment outcomes among silico-tuberculosis patients and their perspectives on implementing collaborative TB-silicosis activities.

## Methods

### Study design and duration

I undertook a sequential explanatory mixed-methods research among patients with silico-tuberculosis in the Khambhat block of Gujarat state’s Anand district. A retrospective cohort study to determine the association of silicosis with unfavorable treatment outcomes of TB was followed by qualitative in-depth interviews. The purpose of the in-depth interviews was to explore the experts’ perspectives on improving the treatment outcomes and to explore the mechanisms of collaborative TB-silicosis activities. The qualitative component of the study was founded on the constructivist paradigm of knowledge acquisition. According to the constructivist paradigm, individuals construct their own perception of the world via their experiences and interactions with others, implying that knowledge is continually evolving and reconstructed as people engage with new experiences and viewpoints^25^. The purpose of this approach was to acquire a thorough understanding of the study participants’ subjective experiences and perspectives. A descriptive design was used to describe the codes and categories generated based on the in-depth interviews. The data for the retrospective cohort study were collected in February–March 2022, while the in-depth interviews were conducted in April-July 2022. The study follows the criteria for reporting cohort and qualitative research^26,27^.

### Study setting

The Khambhat block of Anand district in the state of Gujarat, with a population of 0.3 million, is a city surrounded by rural people. Many companies and small cottage enterprises exist in Khambhat block, where workers cut, rough polish, and fine polish agate stones. The agate stones are primarily composed of crystalline silica, and the processing produces silica dust, which causes silicosis and TB in long-term exposure^1^.

A clinic has been established at the Khambhat block’s Community Health Center to care for and treat patients with TB. Apart from treating patients, this TB clinic also refers X-rays of patients with a mixed presentation of silicosis and silico-tuberculosis to the Civil Hospital (a tertiary care hospital in Ahmedabad) for confirmation of diagnosis. In India, the National TB Elimination Program (NTEP) employs a variety of diagnostic methods for all types of TB, including sputum microscopy, cartridge-based nucleic acid amplification testing, chest X-ray, culture, and histopathology. The program prescribes conventional treatment regimens ranging from 6 months (for drug-sensitive TB) to 20 months (for drug-resistant TB).

There is currently no national health program in India for silicosis. However, chest X-rays or high-resolution computed tomography (HRCT) are used to confirm the diagnosis. The TB unit in Khambhat block has signed an MOU with private radiology centers to offer HRCT at a reduced rate, with payment from the government health center. Only around 5–6% of patients in the specified study setting have an HRCT based on a pneumoconiosis board’s prescription. Special medical boards have been established by state and federal government-run employee schemes to provide compensation following the death of a patient with silicosis. The state government of Gujarat compensates the next of kin with Indian Rupees (INR) 100,000 upon the death of a silicosis sufferer. Additionally, an insured person/family member receives 90% of income in the event of disability under the Employees State Insurance Corporation (ESIC) plan, a social security scheme for workers in India's organized sector^28^.

### Study population

#### Quantitative

I included all silico-tuberculosis patients, aged ≥ 19 years, notified under the TB program in Khambhat block between January 2006 and February 2022 as the exposure group. For the unexposed group, all TB patients aged ≥ 19 years, not suffering from silicosis, notified under the TB program in Khambhat block between January 2017 and February 2022 were included in the study (an adult is defined as a person ≥ 19 years under the TB program in India). I removed patients who may have been exposed to silica dust from the unexposed group, including those found to be working in the agate industry, silicosis misdiagnosed as TB, and mine workers. For retreated cases, the most recent episode of TB was included in the data.

#### Qualitative

Experts in the fields of silicosis, silico-tuberculosis, and industrial hygiene, as well as officials from regulatory agencies, were included in the in-depth interviews. Important points were taken down during the interviews. The interviews were conducted until saturation was reached (i.e., until the same information was obtained in subsequent interviews). Saturation was verified by comparing the notes after each interview.

A total of fifteen in-depth interviews were carried out till information saturation was attained. Four TB program managers in Khambhat block (the district TB officer, a medical officer, a senior treatment supervisor, and a TB health visitor) and two state-level TB program managers were interviewed, all of whom had experience working with patients with silico-tuberculosis. Other interviews included five silicosis experts, two labor department officials, a retired government industrial hygienist, and a senior pulmonary medicine professor at a government medical college. All of the experts, purposively selected in the study, have worked in the states of Gujarat or Rajasthan (the western part of India). None of the experts refused participation in the study.

### Study variables (quantitative)

#### Outcome variable

The national TB program defines treatment outcomes for patients put on treatment for TB^29^. Treatment success is defined when the patients test negative on sputum/culture at the end of treatment (termed as ‘cured’) or, have completed treatment without any evidence of clinical or radiological deterioration (defined as ‘treatment completed’)^29^. ‘Unfavorable TB treatment outcomes’ are defined when patients are categorized as ‘lost to follow-up’ (stopped treatment for at least one consecutive month), test positive on sputum/culture at the end of treatment (termed as ‘treatment failure’), died while on treatment (categorized as ‘died’)^29^. The outcome variable was dichotomous, with unfavorable vs. successful treatment outcomes.

#### Exposure variable

The exposure variable was also dichotomous. The exposed group consisted of silico-tuberculosis patients (those who had both silicosis and tuberculosis), while the unexposed group consisted of TB patients who did not have silicosis.

#### Confounding variables

Age, gender, type of case (new vs. retreated), sputum positive TB, site of TB (pulmonary vs. extra-pulmonary), drug resistance status, human immunodeficiency virus (HIV) positivity and diabetes were the confounding variables. Patients are considered ‘retreated’ when they are restarted on TB treatment after being declared cured (termed as ‘relapse’), following treatment failure, or after being declared ‘lost to follow-up’.

### Data collection

#### Quantitative

The TB unit co-located at the Khambhat Community Health Center was visited for data collection. Since January 2006, the TB unit has maintained a list of patients with silicosis, silico-tuberculosis, and TB among agate workers. The data of patients notified under the program were obtained from the treatment registers provided by the TB program's health staff. The treatment registers contained information on the confounding variables as well as the treatment outcomes of patients.

In 2017, the Nikshay (https://nikshay.in/Home/AboutUs) online portal for patients notified under the TB program was launched. Thus, the treatment records were accessible through the Nikshay online portal in the form of an Excel document for the data between January 2017 and February 2022, with access given by the district TB officer. The data on patients with silico-tuberculosis between January 2006 and December 2016 was obtained from the physical treatment registers and entered into EpiInfo before being downloaded as an Excel sheet.

For ascertaining the exposure groups, the list of patients with silicosis, silico-tuberculosis, and TB was consulted, and a new column (variable) was created in the Excel sheets. Treatment outcomes, at the end of TB treatment, were also accessed from the treatment registers (passive follow-up). The duration of treatment is six months for drug-sensitive TB and 8–20 months for drug-resistant TB.

#### Qualitative

All in-depth interviews were performed by the sole author, who holds an MD degree and is trained in qualitative research methodologies. The investigator, a male, was a researcher at a national institute focusing on occupational health at the time of the study. The in-depth interviews were conducted using an interview guide (Additional file 1) focusing on ways to improve treatment outcomes of patients with silico-tuberculosis and on implementation mechanisms for collaborative activities.

The experts were invited to participate in the study after discussing the purpose and benefits of the study as well as the reasons and interests of the investigator on the topic over the phone. All in-depth interviews took place at a location and time suitable to the experts once they agreed to participate. There was no other person (third person) present throughout the in-depth interviews, which lasted between 20 and 35 min. All the interviews were audio-recorded. There were no follow-up interviews or field notes taken to collect more data.

### Statistical analysis

#### Quantitative

The categorical variables were described in simple percentages while the continuous variables were expressed as median (interquartile range IQR). The baseline characteristics of the exposed and unexposed groups were compared using Pearson’s chi-squared test or the median test. Univariable logistic regression was used to determine which variables should be included in multivariable logistic regression—variables with *p* value < 0.2 were included in the multivariable model. Co-linearity was checked using the tolerance and variance inflation factor (VIF) statistics, with values of tolerance < 0.1 and VIF > 10 considered co-linear. Multivariable logistic regression was performed to determine whether patients with silico-tuberculosis had significantly higher odds of unfavorable treatment outcomes than TB patients without silicosis. Adjusted odds ratios (with 95% confidence intervals CIs) were calculated using multivariable logistic regression. Sub-group analysis was carried out using Pearson’s chi-squared test for the association of silicosis with relapse, drug resistance, death, treatment failure, and ‘lost to follow-up’, and the results were reported as crude odds ratios (with 95% CIs). The differences were considered significant when the *p* value was < 0.05. Statistical Package for Social Sciences (SPSS) version 23 was used for all the statistical analyses.

#### Qualitative

All of the audio-recorded interviews were transcribed into the English language in a Word document. The transcript was coded and the codes were organized into categories (thematic analysis) in an Excel sheet. While assigning the codes, the a-priori idea was to improve the treatment outcomes of patients with silico-tuberculosis and to establish mechanisms for collaborative TB-silicosis activities under programmatic settings. As there had been no previous qualitative studies on this subject, the codes emerged from the perceptions and suggestions of the experts who were interviewed. Inductive reasoning was used to analyze the allocated codes (led by our data). On the initial step of the coding process, known as open coding, broad codes were assigned after splitting down the transcript into smaller bits and naming them, which were then free listed in an Excel sheet. Some codes that reflected the same idea or conveyed the same information were merged, or recoded as singular codes. After analyzing and revising the codes to verify that they appropriately represent the data, a codebook was utilized as a reference that briefly defined each code.

The code assignment was inductive in nature, which means that I started with specific observations and then proceeded on to discover patterns in the data. The open codes were linked to each other in this second step of the coding process, known as axial coding, to establish a linkage between them. Comparable emergent patterns were then allocated as categories and similar categories were grouped under the themes. Important themes that expressed the idea behind the analysis were discovered in the last step of the coding process, known as selective coding. The categories and themes that emerged from the coded data were used to derive conclusions. Any inconsistencies in the data were validated by listening to the interviews again. No additional data was collected to resolve the inconsistencies. The transcript/analysis was not returned to the participants for feedback.

### Ethics approval and consent to participate

Approval for the study was obtained from the Scientific Advisory Committee and the Institutional Human Ethics Committee of the ICMR—National Institute of Occupational Health (Ahmedabad, Gujarat). Permission for conducting the study was obtained from the State TB Operations Research committee (Government of Gujarat). Written informed consent was taken from the participants of the in-depth interviews, which included consent for the audio recording of the interviews. The research was carried out following the Helsinki Declaration. The research team had exclusive access to all data accessed and/or collected throughout the investigation. To ensure patient anonymity, we used the identities given by the National TB Elimination Program to identify the patients (generated through the Nikshay online portal).

## Results

### Quantitative

#### Selection and characteristics of patients

**Figure 1:**
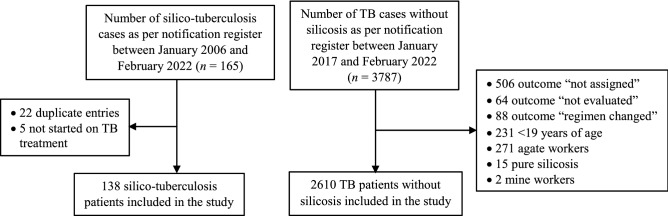
Selection of patients with silico-tuberculosis and patients with TB without silicosis in Khambhat block.

The data were gathered from 138 patients with silico-tuberculosis and 2610 TB patients without silicosis (Fig. 1).

There were no statistically significant differences between the age, weight, HIV status, and sputum positivity of the two groups of patients (Table 1). When compared to the TB without silicosis group, males (79% vs. 66%), chest X-ray positivity (47% vs. 35%), previously treated (51% vs. 22%), multi-drug resistance (7% vs. 2%), relapse (59% vs. 20%), and unfavorable treatment outcomes (39% vs. 18%) were significantly higher in the silico-tuberculosis group. However, higher percentage of TB patients without silicosis were treated empirically than those with silicosis (11% vs. 3%).

**Table 1:** Differences in characteristics of patients with silico-tuberculosis and TB patients without silicosis in Khambhat block.

Characteristics	Silico-tuberculosis (n = 138)	TB without silicosis (n = 2610)	*p* value
	Number (%) or Median (IQR)	Number (%) or Median (IQR)	
Socio-demographic and clinical			
Age (years)	45 (35–53)	42 (30–55)	0.14
Male gender	109 (79)	1716 (66)	0.001
Weight (kilograms)	41 (38–50) (n = 67)	42 (36–49) (n = 762)	0.722
HIV positive	2 (1)	43 (2)	0.858
Diabetic	20 (15)	885 (34)	< 0.001
Tuberculosis			
Sputum positive	78 (57)	1435 (55)	0.723
Chest X-ray positive	65 (47)	906 (35)	0.003
Empirically treated	4 (3)	274 (11)	0.004
Previously treated	71 (51)	571 (22)	< 0.001
Extra-pulmonary	6 (4)	321 (12)	0.005
Multi-drug resistance	10 (7)	51 (2)	< 0.001
Relapsed	81 (59)	508 (20)	< 0.001
Treatment outcome			< 0.001
Successful			
Cured	36 (26)	956 (36)	
Treatment completed	48 (35)	1199 (46)	
Unfavorable			
Treatment failure	11 (8)	43 (2)	
Lost to follow-up	11 (8)	133 (5)	
Died	32 (23)	279 (11)	

IQR: Interquartile Range; HIV: Human Immunodeficiency Virus; TB: Tuberculosis.

#### Association of silicosis with unfavorable TB treatment outcomes

On univariable logistic regression, all the variables had a *p* value < 0.2 (Table 2). However, the tolerance and variance inflation factor (VIF) values for ‘previously treated for TB’ and ‘relapsed for TB’ suggested co-linearity (see Supplementary Table 1). Thus, we removed the variable ‘relapsed for TB’ from multivariable logistic regression as it was a function of the ‘previously treated for TB’ variable (see Supplementary Table 2). Weight was removed from the multivariable model as we did not have data for this variable for all the patients.

**Table 2:** Univariable and multivariable logistic regression^*^ for variables predicting unfavorable treatment outcomes in Khambhat block (n = 2748).

Variables	Unadjusted Odds Ratio (95% CI)	*p* value	Adjusted Odds Ratio (95% CI)	*p* value
Silico-tuberculosis	3 (2–4)	< 0.001	2.3 (1.6–3.4)	** < 0.001**
Age (years)†	1.12 (1.08–1.15)	< 0.001	1.13 (1.11–1.17)	** < 0.001**
Male gender	1.7 (1.4–2.1)	< 0.001	1.4 (1.2–1.8)	**0.002**
Weight (n = 829)	0.99 (0.97–1.004)	0.114	–	-
HIV positive	1.8 (0.9–3.5)	0.075	1.9 (0.9–3.8)	0.057
Diabetic	0.9 (0.7–1.1)	0.155	0.81 (0.65–1.01)	0.057
Sputum positive TB	1.7 (1.4–2.03)	< 0.001	1.4 (1.2–1.8)	**0.001**
Previously treated for TB	1.7 (1.4–2.1)	< 0.001	1.5 (1.2–1.9)	** < 0.001**
Extra-pulmonary TB	0.5 (0.3–0.7)	< 0.001	0.7 (0.5–1.1)	0.105
Multi-drug resistant TB	5 (3–9)	< 0.001	5 (3–9)	** < 0.001**
Relapsed for TB	1.6 (1.2–1.9)	< 0.001	–	–

*Model statistics: Omnibus chi-square = 173 (*p* value < 0.001); Hosmer–Lemeshow *p* value = 0.027; Nagelkerke r^2^ = 0.099; classification accuracy = 82%

^†^Calculated for a five-year increment in age.

Significant values are in bold.

Multivariable logistic regression revealed that silico-tuberculosis, age (years), male gender, sputum positive TB, previously treated for TB, and multi-drug resistant TB were significant predictors of unfavorable TB treatment outcomes (Table 2). Patients with silico-tuberculosis had 2.3 times (95% CI 1.6–3.4) higher odds of unfavorable treatment outcomes than TB patients without silicosis. Each five-year increase in age increased the chances of unfavorable TB treatment outcomes by 13% (95% CI 11%-17%). When compared to their counterparts, being male or having sputum positive TB was linked with 1.4 times (95% CI 1.2–1.8) higher odds of unfavorable TB treatment outcomes, whereas being previously treated for TB was related with 1.5 times (95% CI 1.2–1.9) higher odds. When compared to drug-sensitive TB, multi-drug resistant TB was associated with 5 times (95% CI 3–9) higher odds of unfavorable TB treatment outcomes. The variables included in the model explained 10% of the variation in the outcome variable (unfavorable TB treatment outcomes), which was deemed sufficient by a few researchers^30^.

#### Sub-group analysis

Patients with silico-tuberculosis had 6 times (95% CI 4–8) higher odds of experiencing relapses of TB, 4 times (95% CI 2–8) higher odds of developing drug-resistant TB, 3 times (95% CI 2–4) higher odds of death due to TB, and 5 times (95% CI 3–10) higher odds of treatment failure as compared to TB patients without silicosis (see Supplementary Table 3). Although patients with silico-tuberculosis had a higher rate of ‘lost to follow-up’, the association was not statistically significant.

### Qualitative

**Figure 2:**
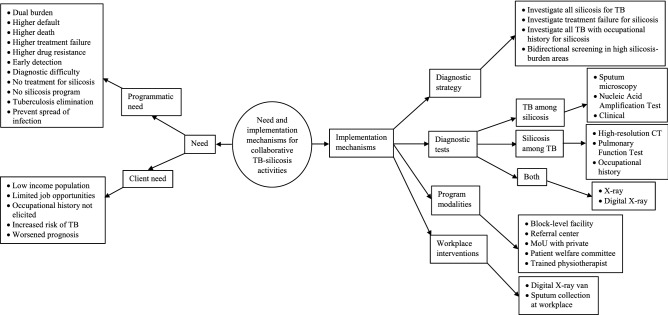
Need and implementation mechanisms for collaborative TB-silicosis activities as perceived by experts during April–July 2022.

**Figure 3:**
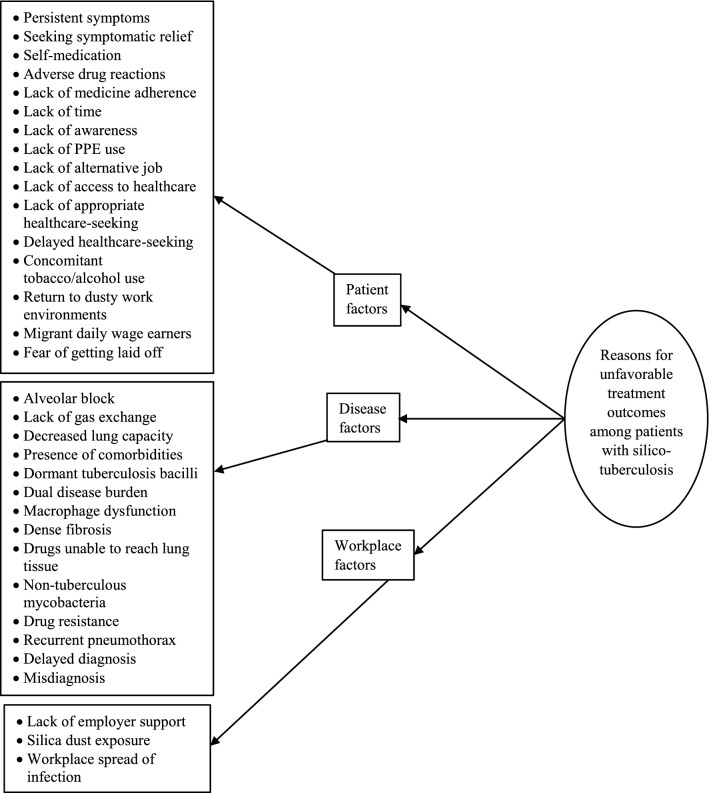
Reasons for unfavorable treatment outcomes among patients with silico-tuberculosis as perceived by experts during April–July 2022.

**Figure 4:**
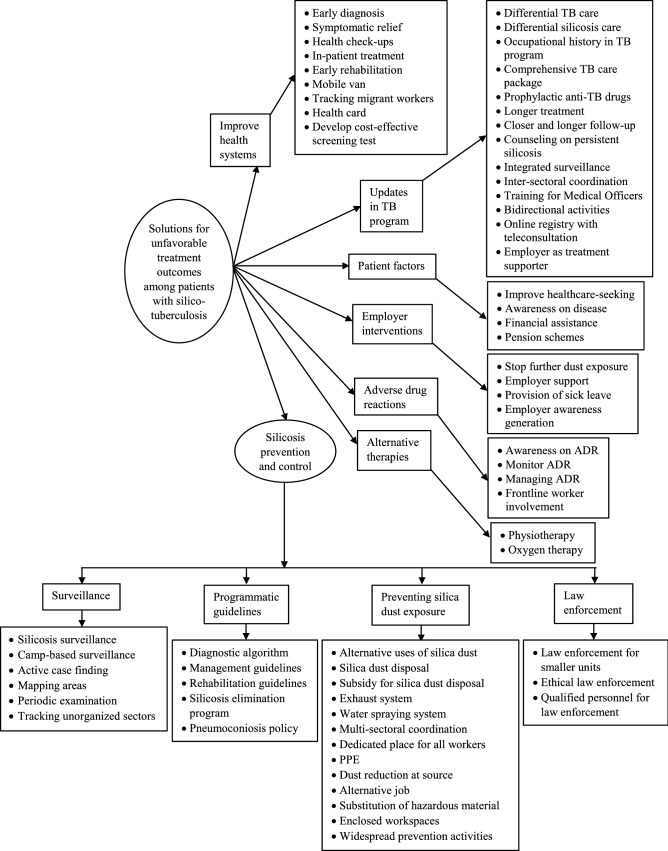
Solutions for unfavorable treatment outcomes among patients with silico-tuberculosis as perceived by experts during April–July 2022.

The median (interquartile range IQR) experience of the fifteen in-depth interviewees, all men except one, was 17 (7.5–27.5) years. The analysis of the codes and categories from the transcript (Additional file 3) revealed four primary themes based on the experts’ perceptions: the need for and implementation mechanisms for collaborative activities (Fig. 2), as well as the reasons (Fig. 3) and solutions (Fig. 4) for unfavorable treatment outcomes.

#### Need for collaborative activities

Experts viewed tuberculosis and silicosis as a twin burden, with higher rates of treatment discontinuation, drug resistance, treatment failure, and death (see Supplementary Table 4, for the description of each need). Because exposure to silica dust makes people susceptible to tuberculosis, it was thought that addressing the rising number of cases of silicosis would be important to keep a control on the number of cases of TB and move closer to TB elimination. The lack of a cure for silicosis, diagnostic challenges in distinguishing the two diseases even on radiography, and facilitating early detection of cases called for the need for collaborative TB-silicosis activities.

“Definitely… the silicosis cases and thereby secondarily affected tuberculosis like silico-tuberculosis cases, both of these are important because the science says that those who are exposed to silica dust, are somewhere or the other immunocompromised and then more susceptible for tuberculosis. Also, tuberculosis happening in these immunocompromised cases of silicosis usually remains dormant, and so, in the favorable condition it may come up as an overt disease. So, definitely, when we want to target tuberculosis elimination by 2030 or by 2025 as India has committed, we have to tackle silicosis cases also side-by-side. Otherwise, these cases and silicosis have a significant burden on our country because we have a lot of mining population, and one of the biggest employers is mining. We have many cases of silicosis though not reported, it is there… and then they may be harboring tubercle bacilli and then our all efforts of eliminating tuberculosis will go in vain if we don’t target these cases.” (Expert in silicosis, 22 years of experience).

#### Implementation mechanisms for collaborative activities

Because silica dust impairs the immune system and increases the risk of developing TB, the experts recommended that all patients with silicosis should be screened for TB (see Supplementary Table 5, for a description of each code). It was also felt that all patients with TB who had a history of occupational exposure to silica dust should be tested for silicosis.

“Those people who are treating these workers, need to be sensitized. As I said that there will definitely be a lack of manpower for occupational health so but then in these high-burden areas where there are pockets and there are likely chances of suffering from silicosis, at least all those people who are dealing with these types of cases, they should be sensitized that whenever a case of cough with sputum production or hemoptysis comes to you, you should also suspect silicosis in them. You are suspecting tuberculosis and giving anti-Koch’s treatment but you should also suspect silicosis in them and then all measures should be taken to prevent the further exposure and thereby arrest silicosis there.” (Expert in silicosis, 22 years of experience).

“In my experience or in my opinion or my perception, I would say each case of silicosis is a potential case of tuberculosis because lungs are damaged, their socioeconomic condition is much worse, their income stops, and the area they live in and you look at all other conditions… many factors which promote tuberculosis are already there, then the hemoglobin also reduces, body resistance reduces and as you know TB is an opportunistic infection because most of us are Mantoux positive, the organism is around us. So, every case of silicosis is a potential case of tuberculosis.” (Expert in silicosis, 40 years of experience).

The experts also recommend that in-patient facilities with X-rays and pulmonary function tests be developed at the block-level Community Health Centers (CHCs) for the execution of collaborative TB-silicosis actions. Experts also proposed establishing a referral center at the district level to which patients requiring advanced care might be referred from CHCs.

“Patients with silicosis are fewer, if you prepare some guidelines where patients get diagnostic facilities like X-ray and PFT at the taluka-level [block-level] and for management of such patients there should be a separate silicosis ward at CHC. If not manageable at CHC then there should be a place where the patient can go for further treatment for free of cost.” (Medical Officer, 12 years of experience).

#### Reasons for unfavorable treatment outcomes

The complementarity of both diseases in causing lung damage, the presence of other comorbidities, the inability of macrophages to remove the TB bacilli, the inability of medications to reach lung tissue owing to fibrosis, recurrent pneumothorax, and concurrent use of tobacco/alcohol were some of the disease-related factors that were thought to lead to adverse TB treatment outcomes (see Supplementary Table 6, for a description of each reason).

“The most important reason is that silico-tuberculosis patients are suffering from two diseases. Treat a patient for tuberculosis, you are curing only one aspect of that… you are not curing the silicotic aspect. What will happen is that these symptoms will not go away fully. Second thing is that immunity is also reduced among patients with silicosis because basically, the primary clearing of tubercle bacilli is through the macrophages, and in presence of silica particles or quartz particles the macrophages cannot effectively clear tuberculosis so the susceptibility is increased. Third thing is that if you treating a patient… I know this about the silicotic aspect… if you are treating the patient, if you know the pathology of the disease, you know that there is dense fibrosis in silicosis. Now in fibrotic tissue, you know that the blood supply is very poor. When a drug is given orally or by injection it has to penetrate those, it has to reach the tissues where the tubercle bacilli are going but the drug may not reach there so the concentration of the drug is not effective and so the drug treatment is not effective. Now second thing is that in many of the studies they have found that in silicosis, there is the presence of not only the tubercle bacilli but also the non-tubercle bacilli. You see e.g. mycobacterium kansasii or avium you know that they are not actually pathogenic but they also have been found to multiply freely in presence of silicosis and usually, they are resistant to most of the drugs so this leads to drug resistance. Drug resistance is caused by the inadequate reaching of the drug to the site of the tubercle bacilli. Second thing is that tubercle bacilli are becoming resistant to the presence of the quartz dust and third thing is that the patient also does not comply because his all symptoms are not going away.” (Expert in silicosis, 25 years of experience).

Because silicosis cannot be treated, patients’ symptoms continue even after TB therapy, leading them to believe that the medications are ineffective and discontinuing treatment. After the initial 1–2 months of intensive anti-TB treatment, patients return to dusty work conditions, increasing their exposure to silica dust even further, and the vicious cycle continues, causing them to develop TB again. Most of these workers are migrant daily-wage earners, making it difficult for them to find alternative employment.

“In these cases, usually what happens… tubercle bacilli many times remain dormant. So under favorable conditions and because these are silico-TB patients, once you give the treatment and once they start feeling better, they will again go back to their job and further expose themselves to the hazardous dust. So that makes favorable conditions and makes them relapse and so more frequently these scenarios are there. This will go into the MDR types of tuberculosis and so the adverse treatment outcomes will be there. The continuous exposure to that environment and improper treatment, because what our experience says that when these people because these are daily wagers, when they get benefited from the initial aggressive phase of anti-TB treatment, they usually return to their job and which is causing all these harms. Again vicious cycle goes on. So they are exposed, they are suffering from TB, then they go to any health care center for treatment to obtain initial relief and again go to work. Continuous exposure to dust is one of the causes of treatment failure in these cases.” (Expert in silicosis, 22 years of experience).

#### Solutions for unfavorable treatment outcomes

Implementing bidirectional activities, halting additional dust exposure, and implementing a comprehensive silicosis elimination program with an emphasis on preventive measures were among the key solutions perceived by the experts for improving the treatment outcomes (see Supplementary Table 7, for a description of each solution). To begin with, silicosis prevention and control should be integrated with the national TB program.

“Given that silicosis may be misdiagnosed or confused with tuberculosis, silicosis patients are very susceptible to tuberculosis, the presence of tuberculosis infection in society, in the community… I feel… I mean nothing can be more important than this bidirectional activity between tuberculosis and silicosis. Both these programs should and must work together. In fact, I am active in the International Commission on Occupational Health… there have been a lot of discussions in the international forum too. Initially, the silicosis activities, silicosis treatment, and detection and all these activities are focused on as independently, but subsequently internationally also it was learned that it will help and it will aid if we combine tuberculosis and silicosis together, then we will be able to diagnose or find out, detect more cases of silicosis and treat them effectively. So it is not only in India but international wisdom also says that both have to coordinate and collaborate with each other and bidirectional activities should be taken up in the right direction.” (Expert in silicosis, 40 years of experience).

Other measures proposed included collecting a complete employment history, treating patients in indoor facilities, giving early rehabilitation, lengthier follow-up, counseling, awareness building, and financial support.

“For improving the outcomes, patients need to be treated indoors for two months or till the time their sputum turns negative or till he is relieved symptomatically… like giving oxygen, IV lines, aminophylline, deriphylline, bronchodilators, steroid injections… using which you can relieve his symptoms. Then the patient might trust you more. He will come to you directly instead of going for expensive treatment at private hospitals and without out-of-pocket expenses, he gets the benefits of treatment. I think all such types of facilities can be implemented in government hospitals—a silicosis ward can be set up with all equipment, and facilities like PFT, and HRCT—so that we can know their lung function and lung damage. If such facilities can be implemented in indoor wards then we can improve the outcomes of silicosis.” (District TB officer, 8 years of experience).

A silicosis elimination program with guidelines on diagnostic criteria, preventative therapy, symptomatic care, dust exposure reduction, and pulmonary and vocational rehabilitation was deemed critical.

“The most important thing about silicosis is the exposure to free silica dust. Now because we find the patients of silicosis it means that the free silica level of dust is high in the working environment and unless we reduce the dust level to the permissible level, the occurrence of silicosis will continue. So the most important thing in the elimination of silicosis is the reduction in the dust level to the level of permissible level or to stop the exposure of workers, changing them to other types of work where there is no free silica dust exposure. Unless we do this the elimination of silicosis is very difficult. What we do is that we try to diagnose silicosis not emphasizing the prevention part of the disease. Most important thing is that it is the dust that causes the disease so we have to stop the exposure to the causative agent… Then, if silicosis is there you must take it seriously because these patients are many times resistant to anti-tuberculosis drugs. If the exposure continues then you are likely to have experienced failure of the treatment, so it is most important that we prevent the dust exposure along with the treatment.” (Expert in silicosis, 31 years of experience).

## Discussion

To summarize the findings, among patients with TB, silicosis was associated with unfavorable TB treatment outcomes and a higher likelihood of relapse and drug resistance. The experts emphasized the need of limiting silica dust exposures at the source for achieving considerable gains toward eliminating silicosis. The experts also perceived that bidirectional collaborative activities between silicosis and TB would help in improving treatment outcomes of anti-TB regimen through early diagnosis and prompt management. The chest X-ray was regarded as a valuable examination for diagnosing both diseases while underlining the necessity for a low-cost screening test for early detection of silicosis.

In the current study, silicosis was also associated with treatment failure and death due to TB, with a higher rate of ‘lost to follow-up’ (treatment discontinuation) among silico-tuberculosis patients compared to TB patients without silicosis. A study on TB among gold miners also reported an increased risk of mortality from silicosis^31^. TB, on the other hand, has been identified as one of the risk factors for mortality among patients with silicosis^13,32^. The present study also found statistically significant higher odds of relapse among patients with silico-tuberculosis who were treated with the standard 4-drug 6-month regimen, which contradicted previous studies using short-course chemotherapies among South African gold miners^33–35^. The present study's failure to distinguish between relapse and re-infection, as well as a lack of data on patient follow-up time to define relapse, may explain the disparity in the researchers' findings. Nevertheless, even after adjusting for other potential risk factors, gold miners were reported to have a significant chance of TB relapse in the presence of HIV infection^36^.

Long-term follow-up among gold miners showed that the relapse rate in the silico-tuberculosis group was marginally higher than in the TB without silicosis group, however this difference was not statistically significant^19^. Further, my study reported that 8% of patients with silico-tuberculosis stopped treatment, while a clinical trial of 6-month vs. 8-month chemotherapy indicated that 12% of patients defaulted and 22% discontinued treatment due to adverse drug reactions^20^. This clinical trial in Hong Kong showed that patients with silico-tuberculosis require at least 8 months of treatment with a four-drug first-line regimen to achieve successful treatment outcomes^20^. Because mechanisms such as oxidative-nitrosative stress and immunological dysfunction play a role in TB bacilli reactivation, the role of novel therapeutics such as corticosteroids or immunomodulators must be investigated^14,37^. Future clinical trials should also investigate the long-term efficacy, toxicity, and outcomes of adding second-line drugs to the conventional four-drug regimen for the treatment of silico-tuberculosis patients.

My analysis revealed that with an increase in age, the chances of experiencing unfavorable TB treatment outcomes rise. This observation was corroborated by other researchers^13,20^, a possible explanation being a 12% yearly increase in chances of silicosis with sustained silica dust exposure^38^. Age is a potential confounding variable in the association between silicosis and unfavorable treatment outcomes, as both age and silica dust exposure can increase over time. Age is associated with an increased risk of silicosis, as well as a higher possibility of an unfavorable treatment outcome, such as death. Despite age being adjusted in the current study’s multivariable analysis, given the lack of data on the levels and duration of silica dust exposure, age may be a reflection of the period of silica dust exposure among the workers.

Experts recognized the need for programmatic guidelines for the management of patients with silicosis in hospital indoor facilities. Additionally, the experts supported collaborative TB-silicosis activities, however, suggested focusing on areas with a high burden of silicosis. Bidirectional screening for TB-silicosis might help in the early diagnosis of either disease, but, X-ray facilities need to be made available in town places. The experts also felt that while TB surveillance was well-established, silicosis surveillance was inadequate in India. Researchers in Zimbabwe and other experts have also suggested surveillance among mine workers for both diseases^39,40^.

Early detection of silicosis and TB among silica-exposed workers has been recommended to prevent further lung damage and limit the spread of TB in the community^40^. Controlling/eliminating silica dust exposure would significantly lower the burden of silicosis and TB^40–42^. Although a very low concentration of silica dust prevents silicosis and the level of exposure for the development of TB is not yet established, reducing silica dust to the lowest possible levels is recommended in India^9,40–43^. Intertwined in a silicosis elimination program, primary prevention interventions such as health education, capacity building, active case finding in high-burden silicosis regions, sentinel surveillance, and rehabilitation would further reduce the burden of silicosis^40,43^. Governments should compensate patients who have developed occupational diseases as a result of silica dust exposure, with the resulting losses being passed on to industries, who may then consider investing in improved engineering controls to limit silica dust exposures^42^. Integrating communicable/non-communicable disease control programs with TB control programs has been proposed for many years^44^, with current evidence favoring integration of the silicosis elimination program with the TB elimination program^3,12^.

Stopping any further silica dust exposures would necessitate the workers’ removal from their jobs, highlighting the need for alternative employment, either by employers in a location where they are no longer exposed to silica dust or by governments through alternative skill-building programs. As pointed out by the experts in the current study, there is a significant possibility that the workers may return to work in dusty conditions after the initial remission of symptoms to sustain their livelihood. It would consequently be required to financially compensate the workers as well as raise awareness about the dangers of continuing exposure to silica dust. A recent research in Rajasthan, a state in western India, noted poor awareness among medical professionals and workers regarding the state government’s financial compensation policy and the Workmen’s Compensation Act^7^. The researchers in Rajasthan also found that many patients with silicosis were not notified since the mines were unregistered, rendering them ineligible for compensatory payments^7^. In addition to stricter enforcement of legislative laws and widespread community awareness, governments should evaluate whether patients with silico-tuberculosis should be offered higher compensation due to the poorer prognosis, and if TB (without radiological silicosis) related to silica dust exposure should be compensated. Providing pulmonary rehabilitation in the form of fitness training, breathing exercises, and other activities, in addition to vocational and financial rehabilitation, would improve their health-related quality of life^45,46^.

This is the first study documenting the treatment outcomes among patients with silico-tuberculosis in the context of TB programmatic settings. Any prospective data collection was avoided since it takes an average of seven years for TB to emerge in individuals after silica exposure ends. Due to the lack of data for all patients, the weight of the patients had to be omitted as a confounder from the multivariable analysis. Because this was a record-based analysis, I was unable to control for several known confounding variables such as nutritional status, silicosis severity, years of employment, occupational history, length of silica dust exposure, smoking status, or any pre-existing comorbidities. Moreover, no information was available on which drugs the patients were resistant to. The in-depth interview guide was not pilot tested, which might have limited the validity and reliability of the data acquired. However, efforts were taken to assure the quality of the data by using structured interview guides and the interviewer’s expertise. Despite these limitations, the findings can be generalized to populations similarly exposed to silica dust elsewhere in India.

## Conclusions

I conclude from the study that co-prevalence of silicosis increases the chances of treatment failure, treatment interruption, death, relapse, and drug resistance among patients with TB. A ‘national silicosis elimination program’ at the block level must be devised through multi-sector engagement, and its implementation must be monitored by the government. In India, X-ray facilities, particularly with high-resolution digital films, should be expanded throughout block levels to aid in the early detection of silicosis. Collaborative TB-silicosis activities should be undertaken in India to achieve significant progress in the prevention and control of silico-tuberculosis. All patients with silicosis should be screened for TB and treated according to NTEP guidelines. All patients with TB who have a history of occupational dust exposure should be evaluated for silicosis and provided appropriate pulmonary/vocational rehabilitation. Future research should demonstrate the effectiveness of bidirectional activities between silicosis and TB in improving TB treatment outcomes and consolidating efforts toward elimination. The author recommends undertaking qualitative exploratory studies or incorporating a qualitative component into observational studies in occupational health to gain rich insights for developing effective and targeted interventions as well as policy that is grounded in a thorough understanding of the needs of the communities they serve.

## Supplementary Information

Supplementary Information 1.

Supplementary Information 2.

Supplementary Information 3.

Supplementary Information 4.

Supplementary Information 5.

## Data Availability

The data for the quantitative component (retrospective cohort) that support the findings of this study are available from the Gujarat State TB Cell but restrictions apply to the availability of these data, which were used under license for the current study, and so are not publicly available. Data for the retrospective cohort component are however available from the authors upon reasonable request and with permission of Gujarat State TB Cell. All data generated or analyzed for the qualitative component (in-depth interviews) of the study are included in this published article [and its supplementary information files].
