# Supplementary material for: A mixed-methods study on impact of silicosis on tuberculosis treatment outcomes and need for TB-silicosis collaborative activities in India
Source: Sci Rep. 2023 Feb 16;13:2785. doi: 10.1038/s41598-023-30012-4 (PMC9935606; doi:10.1038/s41598-023-30012-4)
Supplement: Supplementary file 1 — Supplementary Information 1. [file 41598_2023_30012_MOESM1_ESM.doc]

**In-depth interview guide:**

**Purpose of the interview:**

Good morning/ afternoon/ evening. As has been explained to you, I am here today to talk about the most common comorbidity among silicosis patients, i.e., tuberculosis. You must be aware that globally, we are targeting to eliminate both silicosis as well as tuberculosis by the year 2030. In our study, we found that silico-TB patients are at 2.3 times higher odds of adverse treatment outcomes as compared with TB patients without silicosis. The purpose of this interview is to explore the reasons for the higher odds of adverse treatment outcomes among silico-TB patients, explore interventions that can improve the treatment outcomes among silico-TB patients, and the way forward for implementation of bi-directional activities for silicosis-tuberculosis.

**Informed consent procedure:** Written informed consent for participating in the in-depth interview along with consent for the audio recording of the interview.

**Briefing:**

This is a part of a study by ICMR-National Institute of Occupational Health among silico-TB patients. The research question which is to be answered is “What are the perceptions of program managers of TB and experts in silicosis for improving the care of silico-TB patients and the way forward for bi-directional activities for silico-TB suggested by them?”

It is expected that you feel free to express your opinions. All the information will be kept confidential and anything told by you will not be linked with your names and it will not be disclosed to anyone, so feel free to give your comments and give your detailed opinion rather than just yes or no. Your suggestions can help the government to make appropriate changes to the national TB program. We will begin the recording now.

Can you tell me your designation and the number of years since you are in this position?

**Opening questions:**

1. **India is one of the highest-burden countries for silicosis as well as tuberculosis. What is your opinion on the importance of addressing this dual burden for India, considering that both diseases are targeted for elimination by 2030?**

**Specific questions:**

1. **We found in our study that silico-TB patients are at 2.3 times higher odds of adverse treatment outcomes as compared to TB patients without silicosis. What is your opinion on this?**

**Leading question: What according to you are the reasons for these higher odds?**

**(Probing questions if needed:** That’s interesting, can you give me an example of what you just mentioned……? What do you mean by that…..? Can you please elaborate on that……?)

1. **What can be done for improving the treatment outcomes of silico-TB patients?**

**Leading question: What interventions can be planned for silico-TB patients so that their overall care and management be improved?**

**(Probing questions if needed:** That’s interesting, can you give me an example of what you just mentioned……? What do you mean by that…..? Can you please elaborate on that……?)

1. **What is your opinion on collaborative bi-directional activities between silicosis and tuberculosis?**

(**Probing questions if needed:** That’s interesting, can you give me an example of what you just mentioned……? What do you mean by that…..? Can you please elaborate on that……?)

1. **What would be the implementation mechanisms for bi-directional activities between silicosis and tuberculosis?**

**Leading question: Bi-directional activities essentially would mean that every patient with silicosis gets tested-treated for TB and every TB patient would get tested-managed for silicosis. How exactly can this be done? What would be the tests we can use for diagnosis of each disease and how would they be managed under programmatic settings?**

(**Probing questions if needed:** That’s interesting, can you give me an example of what you just mentioned……? What do you mean by that…..? Can you please elaborate on that……?)

**Exit questions:**

1. **Is there anything else you would like to say that you feel you were not able to say during the interview?**

**De-briefing:**

During the interview, a few of the suggestions you mentioned were………… and a few implementation mechanisms given by you were………….

I thank you for participating actively in this interview.

Can I call you for knowing more about this, in case I need to clarify a few of your answers?
