# Supplementary material for: A mixed-methods study on impact of silicosis on tuberculosis treatment outcomes and need for TB-silicosis collaborative activities in India
Source: Sci Rep. 2023 Feb 16;13:2785. doi: 10.1038/s41598-023-30012-4 (PMC9935606; doi:10.1038/s41598-023-30012-4)
Supplement: Supplementary file 2 — Supplementary Information 2. [file 41598_2023_30012_MOESM2_ESM.doc]

**Supplementary Table 1: Co-linearity statistics highlighting multi-co-linearity between ‘previously treated for TB’ and ‘relapsed for TB’ variables entered in the model for predicting unfavorable treatment outcomes of TB in Khambhat block (n=2748)**

| **Variables** | **Unstandardized Coefficients** | | **Standardized Coefficients** | **t-statistic** | **p-value** | **Co-linearity Statistics** | |
| --- | --- | --- | --- | --- | --- | --- | --- |
|  | **B** | **Std. Error** | **Beta** |  |  | **Tolerance** | **VIF** |
| (Constant) | -.039 | .026 | - | -1.477 | .140 | - | - |
| Age (years) | .003 | .000 | .131 | 7.001 | .000 | .967 | 1.034 |
| Male gender | .047 | .015 | .057 | 3.040 | .002 | .973 | 1.028 |
| HIV positive | .100 | .057 | .033 | 1.773 | .076 | .998 | 1.002 |
| Diabetic | -.029 | .016 | -.035 | -1.856 | .063 | .963 | 1.039 |
| Sputum positive for TB | .051 | .016 | .066 | 3.208 | .001 | .810 | 1.234 |
| Previously treated for TB | .162 | .045 | .176 | 3.606 | .000 | .143 | 7.000 |
| Extra-pulmonary TB | -.028 | .024 | -.023 | -1.172 | .241 | .854 | 1.170 |
| Multi-drug resistant TB | .334 | .050 | .127 | 6.708 | .000 | .952 | 1.050 |
| Relapsed for TB | -.110 | .046 | -.116 | -2.368 | .018 | .142 | 7.026 |
| Silico-tuberculosis | .190 | .035 | .104 | 5.421 | .000 | .924 | 1.082 |

VIF: variance inflation factor; TB: tuberculosis; HIV: human immunodeficiency virus

**Supplementary Table 2: Co-linearity statistics after removing variable ‘relapsed for TB’ for variables entered in the multivariable model for predicting unfavorable treatment outcomes of TB in Khambhat block (n = 2748)**

| **Variables** | **Unstandardized Coefficients** | | **Standardized Coefficients** | **t-statistic** | **p-value** | **Co-linearity Statistics** | |
| --- | --- | --- | --- | --- | --- | --- | --- |
|  | **B** | **Std. Error** | **Beta** |  |  | **Tolerance** | **VIF** |
| (Constant) | -.039 | .026 | - | -1.497 | .134 | - | - |
| Age (years) | .003 | .000 | .131 | 6.980 | .000 | .967 | 1.034 |
| Male gender | .047 | .015 | .057 | 3.064 | .002 | .973 | 1.028 |
| HIV positive | .103 | .057 | .034 | 1.828 | .068 | .999 | 1.001 |
| Diabetic | -.030 | .016 | -.037 | -1.945 | .052 | .964 | 1.037 |
| Sputum positive for TB | .053 | .016 | .068 | 3.341 | .001 | .813 | 1.230 |
| Previously treated for TB | .064 | .018 | .070 | 3.631 | .000 | .927 | 1.079 |
| Extra-pulmonary TB | -.028 | .024 | -.023 | -1.170 | .242 | .854 | 1.170 |
| Multi-drug resistant TB | .328 | .050 | .125 | 6.594 | .000 | .955 | 1.048 |
| Silico-tuberculosis | .175 | .034 | .096 | 5.079 | .000 | .953 | 1.049 |

VIF: variance inflation factor; TB: tuberculosis; HIV: human immunodeficiency virus

**Supplementary Table 3: Association of silicosis with relapse, drug resistance, death, treatment failure, and lost to follow-up in Khambhat block (n = 2748)**

| **Groups** | **Silico-tuberculosis**  **n (%)** | **TB without silicosis**  **n (%)** | **Crude OR (95% CI)** | **Chi-square value** | **p-value** |
| --- | --- | --- | --- | --- | --- |
| Relapsed for TB | 81 (59) | 508 (20) | 6 (4-8) | 120 | <0.001 |
| Not relapsed for TB | 57 (41) | 2102 (80) |
| Total | 138 (100) | 2610 (100) |
| Drug-resistant TB | 10 (7) | 51 (2) | 4 (2-8) | 17 | <0.001 |
| Drug-sensitive TB | 128 (93) | 2559 (98) |
| Total | 138 (100) | 2610 (100) |
| Died | 32 (23) | 279 (11) | 3 (2-4) | 20 | <0.001 |
| Survived | 106 (77) | 2331 (89) |
| Total | 138 (100) | 2610 (100) |
| Treatment failure | 11 (8) | 43 (2) | 5 (3-10) | 27 | <0.001 |
| Treatment failure not reported | 127 (92) | 2567 (98) |
| Total | 138 (100) | 2610 (100) |
| Lost to follow-up | 11 (8) | 133 (5) | 2 (0.9-3) | 2.2 | 0.14 |
| Not reported as lost to follow-up | 127 (92) | 2477 (95) |
| Total | 138 (100) | 2610 (100) |

CI: confidence intervals; OR: odds ratio; TB: tuberculosis
