# Supplementary material for: A mixed-methods study on impact of silicosis on tuberculosis treatment outcomes and need for TB-silicosis collaborative activities in India
Source: Sci Rep. 2023 Feb 16;13:2785. doi: 10.1038/s41598-023-30012-4 (PMC9935606; doi:10.1038/s41598-023-30012-4)
Supplement: Supplementary file 3 — Supplementary Information 3. [file 41598_2023_30012_MOESM3_ESM.doc]

**TRANSCRIPT WITH CODING**

(Click on “Show Comments” under the REVIEW tab for viewing the assigned codes)

**In-depth interview 1: Senior treatment supervisor, 12 years of experience**

**India is one of the highest-burden countries for silicosis as well as tuberculosis. What is your opinion on the importance of addressing this dual burden for India, considering that both diseases are targeted for elimination by 2030?**

It is very important to eliminate it from India and the whole world, but for that, we need to take lots of steps. In India, there are lots of patients with TB and silicosis. Chances of silicosis are higher in those workers who work in Akik [agate] stone, mines, rice mill, tobacco khadi work, and mason work where the dust particles are more in the air. In Khambhat, Akik [agate] industries are more so that need to be well developed. In such industries, exhaust fans must be installed and workers must wear masks and goggles so that dust particles will not enter their bodies hence there will be fewer chances of developing silico-TB. Right now they dilute the silica dust with water but it is again coming in inhalation when this dilute form of the mixture will dry, so there must be a proper way of disposing of it e.g. dispose of it by digging a deep hole and covering it with soil so that it cannot come in the air. There must be mandatory screening (X-ray, sputum, medical checkup) for these workers. Agate industry owner must maintain one register in which there should be details like his name and medical checkup history so that we can check the details when we go for checkup & doubtful cases can be diagnosed again and we can give them proper treatment. For those patients who die due to silicosis, his/her nominee is getting 1 lakh rupees [~US$ 1400] help from the government. However, some patients opined that the benefit should be given when they are alive, which is more important for a good diet and living a better life. For diagnosing TB among patients with silicosis and silicosis among patients with TB, an X-ray of TB patients who visits us at clinics must be checked for the possibility of silicosis. Silicosis patients must be tested for sputum, X-ray, and other medical checkups.

**We found in our study that silico-TB patients are at 2.3 times higher odds of adverse treatment outcomes as compared to TB patients without silicosis. What according to you are the reasons for these higher odds? (Reasons for higher death rate and relapse of silica TB cases)**

The reason is that they are very busy with their work and do not have time to visit the clinic/hospital. Their owner also does not allow them to visit a hospital for medicines. We should go there and meet their owner for checkups of their workers, as early diagnosis is very important. They are not taking medicines regularly even after being diagnosed with TB due to their job chart. For that, we should make the owner a DOT provider and they will treat their workers for six months and cure them. In this way, we may get better outcomes.

**As you mentioned that we do screening at the factory, then how can we do the arrangement of X-ray & other facilities?**

There is a facility of X-ray vans in the Anand district in collaboration with the X-ray house. If we do an X-ray of all workers in each factory compulsorily then we can diagnose silicosis cases easily.

**Does the resolution of the X-ray is of that good quality that can diagnose silicosis?**

A digital X-ray facility is available in a van that can diagnose silicosis. When we send these X-rays to STDC, Ahmedabad, then it will be confirmed about silicosis. We can also do a sputum test.

**Bi-directional activities essentially would mean that every patient with silicosis gets tested-treated for TB and every TB patient would get tested-managed for silicosis. How exactly can this be done? What would be the tests we can use for diagnosis of each disease and how would they be managed under programmatic settings?**

After diagnosis, the DOT medicine packet should be given to the owner of the industry and he will take care of the worker by giving the medicine regularly for six months and ultimately he can track the condition of the worker. By any means, the owner should ensure that the worker must take the medicines on time for six months.

**We are doing bidirectional screening for TB & HIV. What is your opinion on collaborative bi-directional activities between silicosis and tuberculosis? Why?**

Yes, we should do bidirectional screening of silicosis and TB, it will be more helpful. As we don’t know the occupational history (in past they have worked in Akik [agate industry] or not) of the patient coming for TB treatment. Sometimes they tell the occupational history and sometimes they do not. Sometimes even doctors are also not taking occupational history. When the patient comes, they take a sputum sample and start treatment without taking any history. If we take the X-ray and forward it to an expert team for silicosis diagnosis then the early diagnosis is possible, irrespective of whether the patient is giving or doctors taking an occupational history or not. We can start the medicine if the patient is diagnosed with silico-TB. If govt. will provide some benefits to the patient when he is alive then it will be more beneficial for them.

**As you mentioned that we should do their X-ray at the factory through an X-ray van then we can know about silicosis, for TB we need to do sputum for that how can we do it at the factory?**

For that, we need to collect sputum from the factory and diagnose it in the laboratory. We can call them at CHC for HIV and diabetes testing.

**Is there anything else you would like to say that you feel you were not able to say during the interview?**

As per my experience, I have told all the things. If higher authorities will listen and understand us and do something for this from their level, then it will be beneficial otherwise no meaning in our views. They neglect our views and do not consider them. They only consider the views of higher-level employees only. They are not considering the solutions/suggestions of lower-level employees. This thing we have noticed.

**How can we give suggestions to higher authorities?**

If state-level higher authority gives strict guidelines/suggestions to be followed for lower-level authority, then only something can be done. If pressure comes from the state level, then only proper work will be done.

**In-depth interview 2: TB Health Visitor, 5 years of experience**

**India is one of the highest-burden countries for silicosis as well as tuberculosis. What is your opinion on the importance of addressing this dual burden for India, considering that both diseases are targeted for elimination by 2030?**

It is necessary to eliminate TB and silicosis. At present, there are approx. 2.8 million cases of TB, among whom the death rate is something about 1.4 million, which is very high. Many silicosis patients are found in Khambhat. Monthly, we see around 20-25 X-rays of such cases in our OPD, many of them are poor people without any other job so they are involved in this work. If the head of the family suffers from this disease then it is very difficult for the family to survive, so it is important to find such cases.

**We found in our study that silico-TB patients are at 2.3 times higher odds of adverse treatment outcomes as compared to TB patients without silicosis. What according to you are the reasons for these higher odds?**

The first reason is diagnosis. Poor people working in silicosis occupations like Akik [agate industry], if they suffer from cough or any respiratory problem, generally go to nearby clinics like BHMS/ BAMS doctor or, directly to the medical store to take medicines. They do not visit us directly due to a lack of awareness which in turn results in a lack of early diagnosis and an increase in infection. If people directly visit us for a checkup, then early diagnosis and treatment are possible. If such patients visit at nearby govt. clinics/hospitals, doctors need to take occupation history in detail at OPD level, based on which they should suggest X-ray and all related investigations which results in early diagnosis and better outcome. In government set up, X-ray facilities are old, instead of doing MoU for only 2-3 months with private setup, it should be of one year for better diagnosis. Akik [agate industry] workers are very poor and not able to spend money on X-rays in private, so we should concentrate on that. If a patient is diagnosed with TB and silicosis, then they should get proper medicines for six months by the ASHA worker or DOT provider with proper guidance and awareness should be given to the patient about TB and silicosis. ADR is more among patients with silicosis like fever, cough, blood in sputum, etc. for which doctors are not available. DOT providers do not know how to further treat them, therefore, awareness of the patient is necessary. Awareness among the owners of such occupations must be there for handling such patients like giving money to such workers as they suffer from malnutrition. The government is providing NPY [Nikshay Poshan Yojana - 500 rupees (~US$ 7) per month for TB treatment] but beyond that, they should also get some help from the owner of the factory. For example, they should get 15 days of leave for proper rest without a cut in their salary. There should be monthly or quarterly checkups of such patients for early diagnosis and to decrease the death rate and relapse.

**As you mentioned that due to the lack of other jobs, they work in such occupations, for that what else we can do?**

We cannot give employment to all. If we shut Akik [agate] factories, then we need to set up alternative employment for all the workers, which is not possible. We need to set up silicosis industries in such a way that silica dust can be minimized by setting up an exhaust system, water spraying system, wearing masks, hand gloves, goggles, etc. Government can implement a subsidy system for the owner of silicosis industries to encourage them to set up govt. recommended system in their industries. Dust collected as a by-product can be re-used as raw material for manufacturing tiles, blocks, etc. as we cannot throw it anywhere. So owners should be encouraged to implement such systems and follow the recommendations.

**What is your opinion on collaborative bi-directional activities between silicosis and tuberculosis?** **What would be the implementation mechanisms for bi-directional activities between silicosis and tuberculosis?**

In my view, the diagnosis of silicosis is very important. As we are diagnosing HIV and diabetes on an OPD basis, in the same way we can diagnose and treat silicosis patients in the OPD. We can also do campaigns quarterly or annually in those areas where the silicosis industries are located. We can also do ACF as we are doing in TB by our govt. employees. They can do a survey in the industries and find the silicosis patient and refer them to nearby govt. hospitals.

**Bi-directional activities essentially would mean that every patient with silicosis gets tested-treated for TB and every TB patient would get tested-managed for silicosis. How exactly can this be done? What would be the tests we can use for diagnosis of each disease and how would they be managed under programmatic settings?**

We can do a diagnosis of silicosis on an OPD basis by ACF. Patients who say that we are Akik [agate stone] workers are very less. The primary approach can be that of a campaign, we can also do IEC for the owners of industries to make them aware of the campaign. We can reach such patients through OPD and ACF only, which is very less because even if we go there they do not give us a good response. They thought that we go there to shut their industries. Even if we assure them that we are not here to shut your industries, then also they are not coming.

**Which are the diagnostic methods to do bidirectional activities?**

Most commonly we can diagnose silicosis through X-ray. We can also do sputum and CBNAAT. If we do MoU with the private sector, then we can also do HRCT through which we can diagnose silicosis early. MoU with the private sector can be done through higher authorities. We have done MoU through the district TB office for the last two years for X-rays. We have referred something about 80 to 90 X-rays in the last four months from which we found around 45 to 55 silicosis patients. We have MoU for only X-rays so we cannot do HRCT. If HRCT can be included along with X-rays in MoU then it's more beneficial for patients, as these are poor patients and they cannot even afford to do an X-ray in private. This will result in better diagnoses of patients. This can be done through higher authorities only.

**How can you further diagnose TB in silicosis patients?**

We are doing X-rays and sputum among patients with silicosis, if sputum results are positive then it indicates TB… and we have the X-ray findings too to support the diagnosis of TB. Doctors also diagnose TB clinically based on 4 symptoms and we can start medicines. In higher investigations, once they test positive on CBNAAT or TrueNAT, then we can diagnose them as TB.

**Is there anything else you would like to say that you feel you were not able to say during the interview?**

Role after the diagnosis is very important. After the patient is diagnosed with TB and silicosis, then we send them to a DOT provider for further care, we are not concentrating on ADR. It is very important to follow the patient for his six months’ treatment, it’s necessary to do field visits of that patient regularly by staff to know their problems, is the patient taking medicines regularly or not? It might be possible that the patient is not taking the medicines regularly due to ADR. So it’s necessary to avail them of symptomatic medicines for ADR. For that, we should give guidelines and give training to ASHA workers, DOT providers, MPHW, and FHW to upgrade their knowledge. We should also inform the patients about the benefits of completing the course of medicines for six months, if there is any ADR due to medicines then call your DOT provider and visit the govt. hospital without any fear and take medicines. Silicosis patients mainly suffer from breathing-related problems, a lung breathing machine [spirometer] can be at least arranged at CHC level if it is not possible to avail it at all PHCs. We should take the readings of it every two months then it’s beneficial to patients and which is necessary also. Even if the patient suffers more, then also they cannot afford to go to a private setup. So, in my view, we should admit them indoors at nearby CHC.

**How its more beneficial, if we provide a hospital-based facility for silicosis or silico-TB patients?**

We can prevent ADR and if the patient gets good treatment, then other patients will also come to govt. hospital. If he gets good treatment and is cured here, then he will endorse it in his community about the good treatment he got from the government hospital without any cost and also about NPY [cash assistance] he receives. In this way, patients themselves endorse awareness among their relatives and other workers and also encourage them to get treatment at govt. hospital. He will also tell them that they will get good indoor treatment with all facilities by good doctors at the govt. hospital, which will create a good image of the government hospital among the general public.

**Anything more you want to tell?**

No, all my points are covered here.

**In-depth interview 3: District TB officer, 8 years of experience**

**India is one of the highest-burden countries for silicosis as well as tuberculosis. What is your opinion on the importance of addressing this dual burden for India, considering that both diseases are targeted for elimination by 2030?**

Both the diseases silicosis, as well as TB, are notifiable. Both are important as you can see that both have a high death rate. If we want to prevent silicosis, we need to collect surveillance data as we are collecting in case of TB, then only we can bring prevalence aspect. In the case of TB, we are already moving toward its elimination as per the dream of our Prime Minister to eliminate TB in 2025. We are moving in that direction as the figures also indicate the same. But for silicosis, as such there is no program like that, so we are lagging in it. There should be robust guidelines for the vulnerable population for which there should be some diagnostic algorithm according to guidelines, something for preventive treatment, some absolute criteria after diagnosis, then only we can do something more in that. After prevention, if we want to rehabilitate silicosis patients then there should be some guidelines. Unless we make it mandatory or do not bring it under some program, we cannot do anything.

**We found in our study that silico-TB patients are at 2.3 times higher odds of adverse treatment outcomes as compared to TB patients without silicosis. What according to you are the reasons for these higher odds?**

Silicosis patients are mainly concerned about their loss of function. Parts of TB treatment like medicines kill the bacilli or the medicines stop the multiplication of bacilli, and others do not bother them. Improvement in lung function is the only parameter that is important to them. As we know that in silicosis there are irreversible changes, we can see in X-ray that the silica dust gets deposited in alveoli, which cannot be removed by any means. The gas exchange takes place at the level of alveoli, which we can’t prevent. If gas exchange can’t take place, then you do not get oxygen. So patient slowly moves towards respiratory failure. Symptoms of patients are not cured so they think that the medicines are not effective and that increases the tendency of patients to become defaulters. Hence patients will go to another caretaker/ doctor/ hospital, but ultimately the result remains the same. No doubts that the tendency of defaulting on treatment is more in the case of silico-TB patients as compared to normal TB, as patients think that there is no improvement. Deaths are also high in cases of silico-TB as compared to normal TB.

**What can be done for improving the treatment outcomes of silico-TB patients?**

For improving the outcomes, patients need to be treated indoors for two months or till the time their sputum turns negative or till he is relieved symptomatically… like giving oxygen, IV lines, aminophylline, deriphylline, bronchodilators, steroid injections… using which you can relieve his symptoms, then the patient might trust you more. He will come to you directly instead of going for expensive treatment at private hospitals and without out-of-pocket expenses, he gets the benefits of treatment. I think all such types of facilities can be implemented in government hospitals - a silicosis ward can be set up with all equipment, and facilities like PFT, and HRCT - so that we can know their lung function and lung damage. If such facilities can be implemented in indoor wards then we can improve the outcomes of silicosis.

**What is your opinion on collaborative bi-directional activities between silicosis and tuberculosis?**

In my view, if we do unidirectional instead of bidirectional then it’s more beneficial because silicosis occurs in vulnerable populations and is area specific. We cannot prepare generalized guidelines for all areas. Silicosis patients are more prone to TB. But every TB patient is not vulnerable to silicosis. Suppose that a patient is living in a normal residence, where there is no occupational activity that can damage his lungs, then the test of silicosis in TB will be unnecessary. So, if we keep unidirectional, like testing all patients with silicosis for TB, then it would be more helpful.

**What would be the implementation mechanisms for bi-directional activities between silicosis and tuberculosis?**

For the implementation of the bidirectional activities, for diagnosis of TB, we are doing sputum, CBNAAT, and X-ray. But in X-ray, there is a dilemma, so we should do HRCT, and, PFT should be done to know the lung capacity and radiological damages. So we can know the reasons for its proneness and we can keep the cut-off above which it can damage the patients’ lungs irreversibly. Hence, we can try to remove the patient from his occupation for prevention and do rehabilitation at other places.

**Is there anything else you would like to say that you feel you were not able to say during the interview?**

If the government wishes to reduce the burden of silicosis… similar to that of TB elimination… the TB control program was started before the TB elimination program… then we moved toward TB elimination. In the same way, we can start a silicosis control program. We need to identify the areas prone to silicosis which comes under factories and mines acts and need to prepare the proposal to get the surveillance data and start the control program which might take us towards silicosis elimination in the future.

**In-depth interview 4: Medical Officer, 12 years of experience**

**India is one of the highest-burden countries for silicosis as well as tuberculosis. What is your opinion on the importance of addressing this dual burden for India, considering that both diseases are targeted for elimination by 2030?**

In India, the TB burden is 26%. In my view, the burden of silicosis is highest compared to other occupational diseases in India. Under the Industrial and Mines Acts, if we identify the occupations related to silicosis, and based on that if we prepare guidelines, then we can know the exact burden of silicosis and we can further work on that.

**We found in our study that silico-TB patients are at 2.3 times higher odds of adverse treatment outcomes as compared to TB patients without silicosis. What according to you are the reasons for these higher odds?**

Yes, you are true. Symptoms of both diseases are similar. However, the death rate and ‘lost to follow-up’ are higher among silico-TB patients as compared to TB patients. For silico-TB patients, if we start the TB treatment then we know for sure that TB will be cured but the patient does not know this, for them the recovery of symptoms is important. Especially, if agate workers suffer from silicosis or silico-TB, the main complaint among them is breathlessness or low oxygen saturation. If we start treatment for these symptoms - recovery of oxygen saturation level and addition of bronchodilator in treatment part - along with TB treatment, then I feel the patient will take full treatment from us and we can improve the outcome. As per my experience if we add these treatments along with TB treatment then the patient will remain compliant with us till the end. In silicosis, silica dust enters in alveoli, we do not have management guidelines for that, so doctors also do not have any options other than symptomatic treatment.

**What can be done for improving the treatment outcomes of silico-TB patients?**

In my view, as per the ongoing National TB Elimination Program & National TB Control Program, we first controlled tuberculosis, and thereafter we went in the direction of elimination. In the same way for silicosis, if we can prepare guidelines, in which there should be an IEC part, that is, preparation of IEC based on the identification of occupations in which silica is associated. The first part is the IEC part where the public comes to know about occupations that are associated with silicosis, and what they have to follow for prevention. The second part is to prepare the diagnostic tool. The third part is the management part. If we prepare a guideline for this then in my view we can diagnose silicosis early. By keeping in mind the prevention and management part, we can improve outcomes for such patients.

**What is your opinion on collaborative bi-directional activities between silicosis and tuberculosis?**

Under the NTEP program, HIV-TB bidirectional as well as TB-diabetes bidirectional program is ongoing. HIV infection decreases immunity so the chances of TB infection are higher in those cases. When you diagnose TB patients, we understand that immunity is low, so there might be chances of HIV, in such cases, bidirectional activities are good in my view. But if we talk about silicosis and TB, among agate workers or those occupations associated with silicosis, there are high chances of TB infection. But if he is not associated with such occupations then there is no chance of silicosis. A bidirectional program can be implemented in only those identified areas where people are associated with such occupations, then only it will be beneficial.

**What would be the implementation mechanisms for bi-directional activities between silicosis and tuberculosis?**

The NTEP program for TB control has an IEC part, prevention part, diagnostic part, and management part. In the same way, we can prepare a guideline for silicosis with the IEC part, prevention part, diagnostic part, and management part. Under this guideline, if you can do the analysis of the work done and prepare a conclusion, such as, this is the burden of silicosis in this area and then you can prepare a work plan on that in the future. What I see until now is that one-time singular activities are being conducted. For example, using an X-ray van, we are doing the sputum examination and X-ray, and a diagnosis of silicosis is being done… But after that, regular follow-up and analysis of same after a review is not done till the date. If we want to reach toward silicosis control or elimination, then guidelines should be prepared in such a way that regular analysis of the data generated can be done, and regular health checkups of the people associated with such occupations should be done. Finally, such generated data should be analyzed, only then the burden of silicosis can be reduced soon.

**Bi-directional activities essentially would mean that every patient with silicosis gets tested-treated for TB and every TB patient would get tested-managed for silicosis. How exactly can this be done? What would be the tests we can use for diagnosis of each disease and how would they be managed under programmatic settings?**

If we talk about the NTEP program, we all know that for TB symptomatic patients, there is a facility for sputum microscopic analysis, culture, and X-ray diagnosis. But for silicosis, we have tools like X-ray and HRCT. If we do area-specific work like in Khambhat taluka there is a high burden of silicosis, diagnostic tools should be available in such areas e.g., digital X-ray, PFT, etc. All such diseases should be covered under Ayushman Bharat Yojana and one guideline should be prepared for such patients so that if a treatment facility is not available at the taluka or district level then the patient should be referred to so and so place where he can receive all the treatments at free of cost. Similar to a case of TB, if you go anywhere, then he/ she gets treatment at all levels free of cost, expensive treatments like MDR, and XDR can also be availed free of cost. In a real scenario patients with silicosis are fewer, if you prepare some guidelines where patients get diagnostic facilities like X-ray and PFT at taluka level and for management of such patients there should be a separate silicosis ward at CHC level, if not manageable at CHC then there should be a place where the patient can go for the further treatment at free of cost. If we do all these then we can get a better outcome.

**In your opinion is there a role of any sectors which can help in silicosis control and elimination?**

Yes, I think how the health department is working for this disease, in the same way, other departments like the industrial department can work. Under the Factories and Mines Act, there should be proper disposal of silica dust at places where the agate workers are working. I have heard that bricks are made from silica dust, so setting up some facilities for preparing bricks from silica dust would be helpful. Many agate workers are working at home so there is difficulty doing their health checkups twice a year. If we set up a place/ industry for agate workers instead of home-based work, like the diamonds workers working at Surat or Ahmedabad, then we can maintain the registration of agate workers & we can do their health checkups twice a year, then there might be the possibility of reduction of the silicosis cases in future. So the involvement of other departments through the District Collector is important.

**Is there anything else you would like to say that you feel you were not able to say during the interview?**

All the things are covered but I will surely say that there is a necessity for the involvement of other departments, along with that of the health department - the District Collector should help in such an integration. Initiation, implementation, and review of the silicosis control program, similar to the TB elimination program, would help decrease the burden of silicosis.

**In-depth interview 5: State-level TB Program Manager, 17 years of experience**

**India is one of the highest-burden countries for silicosis as well as tuberculosis. What is your opinion on the importance of addressing this dual burden for India, considering that both diseases are targeted for elimination by 2030?**

It is very important because the TB burden & infection prevalence is more in our country. There may be around 2.6 million patients with TB - accounting for one-fourth of cases in the world - in India and there are lots of missing cases also. If we talk about silicosis, there are high chances of silico-tuberculosis. In our TB control program, silicosis is considered a vulnerable population because lung capacity is reduced in silicosis, and TB infection prevalence is more so the PFT of silicosis patients is altered and there are more chances of infection due to lung issues among them. In the general population lifetime risk of TB is 10%, but among patients with silicosis, lifetime risk increases, so there is a high risk of TB infection among them. We need to focus on silicosis elimination as much as we are focusing on TB elimination. There need to be control strategies for silicosis elimination also. This thing is already included in our control program and work is also going on in that like we are doing CBNAAT testing among vulnerable populations.

**We found in our study that silico-TB patients are at 2.3 times higher odds of adverse treatment outcomes as compared to TB patients without silicosis. What according to you are the reasons for these higher odds?**

Yes, it’s true. As compared to the general patient, among comorbid patients (like silicosis, HIV, diabetes), there is less sputum conversion and we have not achieved the required success rate. There are chances of patient death due to comorbidity. If the symptoms do not subside then it may result in a ‘lost to follow-up’ of patients. So there are chances of less success rate, an increase in patient death, and ‘lost to follow-up’.

There are lots of reasons for unfavorable treatment outcomes like a patient suffering from silico-TB has decreased lung capacity and altered PFT. If the symptoms of patients with silicosis - coughing, and evening rise temperature - do not subside then it may result in the patient ‘lost to follow-up’, which may result in a low success rate. People work without personal protective equipment in the industries where chances of silicosis are higher. They may also have other comorbidities. Diabetes prevalence is higher in our country and Gujarat, so there is no sputum conversion among the patients. Most silicosis patients have the habit of alcohol and smoking due to stress which precipitates more.

**What can be done for improving the treatment outcomes of silico-TB patients?**

We can do two-three things in that. There is one thing called ‘differential TB care’ given in the guideline, in which when a patient is diagnosed with TB, his oxygen saturation, PFT, TC-DC, and respiratory rates are monitored, and accordingly, data is disaggregated. Patients get treatment according to that, if the patient is anemic then he/she gets anemia treatment, if oxygen saturation problem then oxygen therapy is given and if silicosis then necessary medicines should be given. So in my opinion ‘differential TB care’ can be included when a patient is diagnosed with TB. If there is a patient with silicosis then, the patient should get ‘differential silicosis care’ treatment similar to that of ‘differential TB care’ after identifying other underlying diseases. If we focus on that then the death rate can be reduced.

**What is your opinion on collaborative bi-directional activities between silicosis and tuberculosis?**

There are maximum cases of HIV and diabetes among vulnerable populations. Collaborative activities are already there in the program and its guideline is also available. In the same way, there is a need for guidelines in silicosis also like four symptoms complex screening of silicosis patient, and upfront CBNAAT after screening if symptoms are there. If there is a TB patient, then we can do an X-ray and see if silicosis is there or not. We can also identify the silicosis by taking the patient history like his area of work (industries where silica is generated, mines, construction, etc.). Working history should be included in the program. Pre-identification is important to minimize lung damage among such patients. Physiotherapists can be consulted to improve their lung capacity by doing some lung exercises to prevent further problems.

**What would be the implementation mechanisms for bi-directional activities between silicosis and tuberculosis?**

**Leading question: Bi-directional activities essentially would mean that every patient with silicosis gets tested-treated for TB and every TB patient would get tested-managed for silicosis. How exactly can this be done? What would be the tests we can use for diagnosis of each disease and how would they be managed under programmatic settings?**

As I said, for the HIV positive, diabetic, antenatal patients we are doing four symptom screening where we see cough for more than two weeks, for HIV positive any duration of cough, same way for silicosis if we find any duration of cough, fever, evening rise temperature and weight loss in patients then we can do screening and then CBNAAT. If CBNAAT turns negative, then we should do an X-ray. For silicosis patients, we can do such four symptoms screening. For TB patients, we must ask about their history of work. If the patient is working in an area where silica dust is generated, then we should suggest an X-ray as it is a screening tool in silicosis diagnosis. No doubt that there may be symptoms like cough, breathlessness, and hemostasis but an X-ray is a more sensitive tool as compared to symptomatic screening, so if there is history then we must do an X-ray.

**In your opinion how sensitive is an X-ray? Does simple X-ray work for silicosis diagnosis?**

According to national-level guidelines, digital X-ray does not have validity as conventional X-ray. If we see according to the guideline, then a conventional X-ray is good but in my experience, a digital X-ray is necessary. If we look one step further, then HRCT has better sensitivity. In X-ray, if there is bilateral silicosis and miliary tuberculosis then we see millets in both cases. At the grass-root level, the medical officer cannot differentiate between both. So, in my view, HRCT is a highly sensitive tool rather than an X-ray.

**We do have a well-established guideline for the management of TB patients but what can be done for silicosis? Please give your guidance.**

Govt. of India targeted to eliminate TB by 2025, if we want to eliminate silicosis then active search is necessary because when we talk about eliminating any disease like TB or malaria, active search is very important. In my view right now passive work is going on. Rather than passive work, we need to focus on active case finding & do field surveillance. There is a surveillance form that the health workers fill out in the field, we need to include the working history in that - is the worker working in silica dust-generating industries? - so that we can find silicosis cases from surveillance. We need to strengthen active search so that we get the cases that are hidden in the field and hence early detection is possible. Early detection helps in rehabilitation and we can isolate those cases from silica work and advise them to use suitable personal protective equipment. We get many such cases in the field with a late diagnosis that can’t be treated as once silicosis is diagnosed, there is no treatment. So, early detection of such cases is very important to rehabilitate them and provide the necessary care. If they suffer from TB symptoms like any duration of cough, then we can do their immediate counseling. An active search is a very important tool and can be helpful.

**You have earlier mentioned differential silicosis care. Can you explain about it in detail what exactly it is? Can we implement the bidirectional and collaborative activity in it?**

As I mentioned there is one guideline named differential TB care. As per that guideline, we can know earlier about the risk of the patient like whether it is a high-risk patient or not. For example, sometimes patients die due to late diagnosis or due to comorbidity, or maybe an underlying disease. During the initial diagnosis, the patient is identified and categorized into a high-risk or low-risk group. If a patient falls into a high-risk group, then he/she can be referred to the appropriate level.

Guidelines for such differential silicosis care do not exist. As per my thought, we can develop such guidelines for silicosis in which we can put certain criteria like oxygen level, PFT, breathlessness, lung-related tests, or any other symptomatic criteria in differential silicosis care. We can use it as a screening tool for those who are working in silica dust exposure areas for early diagnosis. If a patient can be diagnosed at the initial level then he/ she can be referred to the appropriate level and further problems can be prevented. Early detection helps us to refer them to a physiotherapist or any counselor. If we include it in the NCD program then we can also refer such patients to NCD counselors who can counsel such patients and prevent them from future problems. Counselors are available under the NCD program. As silicosis is also a non-communicable disease, ‘differential silicosis care’ can be included in the NCD program and some counseling tools can be given to the health care workers so that if patients come to them then active cases can be identified. There is a need to find high-risk areas in the state and then search for high-risk populations and refer them to a counselor so that early diagnosis of such cases is possible and awareness can also be generated among them.

**Is there anything else you would like to say that you feel you were not able to say during the interview? like unfavorable treatment outcomes, interventions to improve outcomes, collaborative bi-directional activities, etc.**

PFT machines should be available at all levels at least at the community health center level. For example, if we send the patients to the district level where all the facilities are available then the chances of missing the patients are higher & due to financial issues, patients will not go to the district level. If facilities like PFT machines for the identification or any other diagnostic tool or machine are provided at least at three levels i.e., community health center, district level, and sub-district level, the patient’s traveling time will reduce, and the maximum number of PFT will be possible for that patient which results in early detection. If HRCT is advised by the doctor for a patient then there is Rogi Kalyan Samiti at the PHC level. The state should provide funds/ provisions to Rogi Kalyan Samiti for HRCT purposes so that patient costs can be reduced. For example, if we have done an X-ray of any patient and if it is doubtful and HRCT is advised by the doctor then there should be a mechanism to bear its expense at the PHC level by Rogi Kalyan Samiti so that patient can be benefitted. If we want to link (refer) such patients with a physiotherapist then there is no such facility available. A physiotherapist is available at a community health center but they do not know which exercises need to be given to such patients, so they need training about the exercises/ therapy that needs to be given in such cases when they come to CHC. Staff/ human resources are available but they are not properly trained about how to give exercises to such cases of altered lung functions. They need training and awareness. So these two-three things are important i.e. PFT availability, trained physiotherapist at CHC level & development of tools for active search/ finding of such silicosis patients. When a patient is diagnosed, there is a need to prepare a document for differential silicosis care similar to the differential TB care and then the patient needs to be segregated into categories like high risk & can be referred to the appropriate level for further treatment.

**In-depth interview 6: Expert in silicosis, 31 years of experience**

**India is one of the highest-burden countries for silicosis as well as tuberculosis. What is your opinion on the importance of addressing this dual burden for India, considering that both diseases are targeted for elimination by 2030?**

I agree with you that both the disease silicosis and tuberculosis are dangerous for our community and we must take preventive measures to eliminate them and save our community. Now about the bidirectional study of these two diseases, I think it is also useful but I would suggest that wherever the workers are exposed to silica dust, there are certain industries where these workers are exposed to silica dust and there are certain areas where these industries are situated, this study should be undertaken in those areas. Otherwise, in the overall population, this study will not be useful, so this is most important. Another thing is that as far as silicosis tuberculosis is concerned, the outcome with the anti-tuberculosis drug is sometimes not good and patients do not get much relief with the AKT drugs. The reason could be the exposure to silica dust may be continued and the drugs are somehow resistant in those cases and that is why I think that the outcome is not good. What I think is that to improve the outcome among patients with silico-tuberculosis, the first thing should be stopping the dust exposure. If we can do that then I think that there may be an improvement in the outcome in those silico-tuberculosis patients but unless we study this, it is difficult to say.

**Can you suggest other interventions on the clinical side that we can implement or which might be helpful to improve the treatment outcomes of silico-TB patients apart from reducing silica dust exposure? Does anything come to your mind by which we can improve the outcomes among silico-TB patients?**

The most important thing about silicosis is the exposure to free silica dust. Now because we find the patients of silicosis it means that the free silica level of dust is high in the working environment and unless we reduce the dust level to the permissible level, the occurrence of silicosis will continue. So the most important thing in the elimination of silicosis is the reduction in the dust level to the level of permissible level or to stop the exposure of workers, changing them to other types of work where there is no free silica dust exposure. Unless we do this the elimination of silicosis is very difficult. What we do is that we try to diagnose silicosis not emphasizing the prevention part of the disease. Most important thing is that it is the dust that causes the disease so we have to stop the exposure to the causative agent.

**You mentioned that collaborative bi-directional activities between silicosis and tuberculosis would be helpful for the program as well as for the patient. What would be the implementation mechanisms for bi-directional activities between silicosis and tuberculosis?**

Yes, wherever you find the cases of silicosis in the industries or silica dust exposed areas you can also suspect patients for tuberculosis, and not only that but you can examine them by X-ray or sputum examination or the CBNAAT so that you can find out tuberculosis among silico-TB patients. So once you get a patient with silico-tuberculosis, you must isolate this patient and stop the exposure and then start the AKT treatment accordingly. Then you can see the outcome of those patients and how many of them are improved and how many of them do not improve that you can say, so that is required to be studied. Then about tuberculosis, again in the area where the silica dust exposure or industries are there, you can take the patients with tuberculosis and you can examine them for silicosis. To find out the silicosis the most important thing is the X-ray examination and if needed in doubt cases you can also undertake a CT scan of the thorax so these two will try to give you the idea of silicosis. Then, if silicosis is there you must take it seriously because these patients are many times resistant to anti-tuberculosis drugs. That is why they need immediate removal from the exposure and then treatment should be carried out. If the exposure continues then you are likely to have experienced failure of the treatment, so it is most important that we prevent the dust exposure along with the treatment.

**We do have well-established guidelines for the management of TB patients however when it comes to silicosis usually clinicians are confused as well as there are no management guidelines for patients with silicosis. Can you throw some light on how in a better way can we manage those patients or maybe improve them clinically in some manner when we diagnose silicosis among patients with tuberculosis?**

You see both diseases cause fibrosis in the lung. They are rather helping each other and that is why the condition of silico-tuberculosis is bad. Now it is also known that sometimes even after stopping the dust exposure of silica, the patient continues the disease, and sometimes it develops to the severe stage of the disease. It is not certain that we will be able to prevent the outcome of the silico-tuberculosis patient or tuberculosis treatment but to our knowledge, if we stop the dust exposure, probably there are more chances of improving the outcome of the silico-tuberculosis patient. We must emphasize for those patients (silico-tuberculosis patients) that we stop the silica dust exposure.

**Is there anything else you would like to say that you feel you were not able to say during the interview? Regarding any of the aspects that we just cover like the importance of bi-directional activities or how to implement those bi-directional activities or the treatment part or the diagnosis part how to diagnose TB among silicosis or how to diagnose silicosis among TB? If you feel that anything more to say you can say it now.**

Yes, definitely. See the most important thing is the diagnosis and other things are easy, to my mind. The most important thing is exposure. Now unless you stop the exposure to any toxic things or any material which is causing the disease, you cannot expect the disease to stop. When you use the word elimination, it means that elimination can only be achieved by reducing the exposure or stopping the exposure to the permissible level. So if we don’t achieve this thing, our all efforts will be a failure. It is not the treatment; it is stopping the exposure, which is to my mind more important than the treatment. Probably, those who are working on silicosis, always give more emphasis on testing and diagnosis and all those things but not on stopping the dust exposure which is to my mind the most important part of the elimination of silicosis.

**In-depth interview 7: Expert in silicosis, 22 years of experience**

**India is one of the highest-burden countries for silicosis as well as tuberculosis. What is your opinion on the importance of addressing this dual burden for India, considering that both diseases are targeted for elimination by 2030?**

Yes, definitely the silicosis cases and thereby secondarily affected tuberculosis like silico-tuberculosis cases, these both are important because the science says that those who are exposed to silica dust, are somewhere or other immunocompromised and then more susceptible for tuberculosis. Also, tuberculosis happening in these immunocompromised cases of silicosis usually remains dormant and so in the favorable condition they may come up as an overt disease so definitely when we want to target tuberculosis by 2030 or by 2025 as India has committed, we have to tackle silicosis cases also side by side. Otherwise, these cases and silicosis have a significant burden on our country because we have a lot of mining population, and one of the biggest employers is mining. We have many cases of silicosis though not reported it is there and then they may be harboring tubercle bacilli and then our all efforts of eliminating tuberculosis will go in vain if we don’t target these cases.

**We found in our study that silico-TB patients are at 2.3 times higher odds of adverse treatment outcomes as compared to TB patients without silicosis. What according to you are the reasons for these higher odds?**

I think you found 2.3 times higher odds but I say that it is somewhat of an underestimation because in our study we did in 2001-2002 we found almost 10 times higher risk in those who are exposed to silicosis. Even literature suggests 10 times of 13 times higher risk in those who are exposed to silicosis.

**Our study was finding out the risk of adverse treatment outcomes like treatment failure, loss to follow-up i.e. treatment interruptions, and death among silico TB patients. We found 2.3 times higher odds of all these three combined i.e. higher treatment failures, higher treatment interruption, and death were higher among silicosis patients as compared to TB patients without silicosis. So my specific question is what is your opinion on the higher odds of adverse treatment outcomes as compared to TB patients without silicosis? What according to you are the reasons for these higher odds?**

As I assume that the relapse was one of the adverse treatment outcomes. So as I said that in these cases usually what happens tubercle bacilli many times remain dormant. So under favorable conditions and because these are silico-TB patients, once you give the treatment and once they start feeling better they will again go back to their job and further expose themselves to the hazardous dust. So that makes favorable conditions and makes them relapse and so more frequently these scenarios are there. This will go into the MDR types of tuberculosis and so the adverse treatment outcomes will be there. The continuous exposure to that environment and improper treatment, because what our experience says that when these people because these are daily wagers, when they get benefited from the initial aggressive phase of anti-TB treatment, they usually return to their job and which is causing all these harms. Again vicious cycle goes on. So they are exposed, they are suffering from TB, then they go to any health care center for treatment to obtain initial relief and again go to work. Continuous exposure to dust is one of the causes of treatment failure in these cases.

**What can be done for improving the treatment outcomes of silico-TB patients?**

**Leading question: What interventions can be planned for silico-TB patients so that their overall care and management be improved?**

First thing is that, once you have established that it is silico-TB cases, this means there is some sort of exposure to silica dust, which is happening. The first and foremost thing is that you have to intervene at the exposure level so further exposure should be avoided and that will be beneficial for the treatment of these patients. Again when you say that treatment outcomes need to be improved so all other parameters which are associated with TB will be seen in these patients like nutrition will be affected, or other opportunistic infections will be there, so all these things need to be dealt with in a comprehensive package so that the outcome of treatment of TB in these patients can be positively affected.

**By comprehensive package do you mean something as similar on the lines of the TB elimination program? Do we have any silicosis control or silicosis elimination program? Does anything exist in India?**

It is started in 2009, the silicosis elimination program. It came up with a very good objective of attacking silicosis but the problem is that majority of these people who are exposed to this hazardous dust, fall beyond the purview of the law. They are usually in an unorganized sector and so they won’t avail any health care facility, they have to do of their own, neither the employer nor any other agency providing these people so definitely they are not having all those provisions which are there for their health & safety, which makes the scenario more complex. But then having said that like we have TB cases like those who are under the NTEP program, we are providing the complete package like we are providing some money for food, then we are providing free of cost drugs, we provide the regular follow-up & investigations and all those things which is not present in the cases of those who are working in silica dust occupations because largely many times these are migratory populations so registering these populations at one place, a lot of complex issues are there. These issues need to be tackled and they should be provided with all those provisions which are there for TB patients under the NTEP program. In addition to that at the workplace level also you have to do the management so you have to regularly screen these people for any adverse effect of either silica dust or opportunistic infection of tuberculosis.

**You mentioned that as soon as we come to know that the silico-TB patients are exposed to silica dust, we need to ensure that further exposure to silica dust should be reduced. Can you elaborate more on that how can we reduce the further exposure to silica dust among these patients?**

See the problem is that as I said these are daily wagers, these are migrant workers, and they have very limited choices for earning their livelihood. The first and foremost and most important and effective way of reducing exposure is you remove that person from further exposure, by way of a change of job. Now usually what happens is that all these people are self-employed, nobody is deciding on their behalf of them. So when you say that person to get removed from that silica dust occupation then we have to provide some other alternative mechanisms of earning livelihood where such exposure is not there and a person can earn. Because what will happen when you remove that person from such exposure if he has not had the source of earning or livelihood then the person may go into under nutrition and we all know that under nutrition is again a risk factor for tuberculosis. So the first thing is that you remove that person from the job and search for some alternative job for that particular person. Now, if that is not possible because of any reason, then at the workplace they have to implement all the control measures at a personal level and the workplace level. All those methods are well known like industrial hygiene methods like the exhaust mechanism should be there, PPE should be there, the materials should be generating lesser dust and then dust-generating mechanisms should be there, the enclosure should be there, fewer numbers of people should be there in the hazardous process so all the standard methods which are there for preventing exposure needs to be implemented at the workplace.

**What is your opinion on collaborative bi-directional activities between silicosis and tuberculosis?**

It is bidirectional because those who are affected by silicosis, are susceptible to tuberculosis and similarly those who are having tuberculosis, will be having more effects from smaller concentrations of dust also. Having said that what is happening actually at the ground level is that those who are affected by silicosis or silicosis-like symptoms while working in dusty occupations, usually the first point of choice for them is any DTC nearby, so it is assumed that they are having cough for a longer period. Whatever is given in the textbook, like 15 days or more cough, so usually they approach the DTC nearby and there without asking their occupation which is the cause, which is precipitating all those symptoms they are directly put on the anti-tuberculosis drugs. And so what happens, as I said once you give the aggressive phase of tuberculosis treatment, the person will start feeling better may be by one month or two months, and then occurs the defaulting and person again goes back to his occupation. So what is said is that whenever a person and particularly in the pockets where we know that there is mining activity which is going on or other activity which is causing silica dust exposure. So wherever these types of patients are approaching tuberculosis treatment care center, the occupation should be asked and if it is found that the person is working in such occupations, no doubt tuberculosis treatment needs to be done but at the same time, the confirmation for the exposure as well as any symptoms of silicosis should be taken care and immediately at that point of time itself the further exposure should be curtailed. So that whatever treatment, because in our experience we have seen in the past that people have received one and half year treatment without any effect and this is because they were treated for tuberculosis but the underlying thing was silicosis or silico-TB. So the person was taking treatment, going back to the job, and getting again treatment of tuberculosis, so this was going on. So those have to be targeted at the same time if you want to have an effective treatment for these groups of patients.

**You mentioned the diagnosis of TB among silicosis patients. What is your opinion on diagnosing silicosis among all TB patients? Because essentially when we talk about bidirectional activities, it would mean diagnosing silicosis among all TB patients and diagnosing TB among all silicosis patients. So I mean logically we feel that yes since silica exposure leads to silicosis as well as TB independently, it makes sense to diagnose TB among silicosis patients, but what about diagnosing silicosis among all TB patients?**

I don’t agree with that, there are two-three reasons. First thing is that if the person is having tuberculosis, which may not necessarily be working in any occupation where silica exposure is there. Another thing is that suppose if we make a policy decision that all the tuberculosis patients will be diagnosed with silicosis then the serious issue will be there, do we have the manpower to diagnose silicosis in each tuberculosis case? So that policy may, or may not work because we lack resources for diagnosing silicosis. Based on the occupation itself, you will get a hint that whether the person may be having silicosis or not. So maybe occupational history is sufficient to give you a clue. So diagnosing tuberculosis in a silicosis case is important but diagnosing silicosis in each tuberculosis case has lots of logistic constraints.

**We understand that there are pockets mostly in all states e.g. Khambhat in Gujarat, Vidisha in Madhya Pradesh, and Jodhpur in Rajasthan, these are high-burden silicosis areas. What is your opinion on actual bidirectional activities for silicosis and tuberculosis in those high-burden districts maybe?**

If you see these high pockets areas and if you study the profiles of the workers who are there, usually they are migrant workers so they are floating workers, today they will be in one workplace tomorrow they will be at another, so one thing is that there should be the mechanism of registering all these peoples. So that you have a track of all these people because even if you are going to give treatment for tuberculosis or even if you want to prevent them from dust exposure, it has to be for a certain period and these people usually change their job. The first is that you have to have a central repository of all these workers who are working in these occupations. Then the people who are working in the unorganized sectors should be regularly screened for silicosis and tuberculosis. These are two important things that can help you in finding more and more susceptible populations and then we can go for the effective treatment.

**Under the NTEP guidelines, we have definite algorithms for treatments for drug-susceptible as well as drug-resistant TB. However, when it comes to silicosis we do not have any specific guidelines for how to manage patients with silicosis. Based on your experience, can we come up with some guidelines may be on how a patient with silicosis should be managed, or what is your experience on that?**

The foremost guideline and which we also have experience with and textbook also say that the first and foremost thing is that you have to stop the further exposure to dust because it is a progressive disease even if it is initiated, it is going to progress but then if you are curtailing the exposure, at least the rate with which it will progress can be hampered. So first and foremost thing is that silica exposure should be immediately curtailed. Now having said that it is not very easy also because as I said that you have to come up with some alternate way of earning a livelihood. Another thing is that as we do in many of the organized sectors, there is a mechanism of a periodical checkup or periodically screening of these people. So some mechanism should be there either aligned to some TB center nearby or some health care facility. All these workers who are working there should be subjected to some periodical checking so that we can detect the silicosis at the earlier stage or TB at an earlier stage and stop it there only. So these are some of the ways through which we can act. But then frankly speaking because of many of the logistic and administrative issues, we also tried but it doesn’t become feasible. We have worked in the quartz industries again the same problem is there. Agate, we have worked; we have asked people and could not find alternate occupations there.

Then those people who are treating these workers, need to be sensitized. As I said that there will definitely be a lack of manpower for occupational health so but then in these high-burden areas where there are pockets and there are likely chances of suffering from silicosis, at least all those people who are dealing with these types of cases, they should be sensitized that whenever a case of cough with sputum production or hemoptysis comes to you, you should also suspect silicosis in them. You are suspecting tuberculosis and give anti-Koch’s treatment but you should also suspect silicosis in them and then all measures should be taken to prevent further exposure and thereby arrest silicosis there. So that also needs to be done.

**Any comments on the treatment or supportive treatment, I would say, for silicosis patients?**

Supportive treatment, usually because of the breathlessness they get supportive treatment but then many times these supportive treatments will be misleading because if you are giving supportive treatment and the patient feels better and again he will go to the job and get himself exposed. So I think the supportive treatment has very little role unless or until you have diagnosed that this is a case of silicosis. Once the silicosis case is diagnosed, definitely you will not allow the person to go to the same job, at that time you can give some supportive treatment but then on day to day basis if you are giving supportive treatment the person will feel better and again he will go to the same workplace.

**We talked a lot about the management aspect, can you throw some light on if at all we want to establish such bidirectional activity, and what investigative measures/ diagnostic tools can be used for diagnosing TB among silicosis and vice versa (silicosis among TB patients)?**

Diagnostic tools like sputum AFB and chest X-ray are two important investigations, which can solve a lot of issues for diagnosis. There are other biomarkers also but the first thing is that these are not usually done so the chances of getting this test done in a nearby pathology lab or other labs are very cumbersome. And again many of these are non-specific so if you go for that it will unnecessary burden to the patient and system, and if you are going and saying to the employer most of the time if the cost is on the higher side then they will prefer not to do it rather than to do it. So very gold standard mechanism is the X-ray, which will give you a lot of hints and for tuberculosis, we all know that sputum AFB is the gold standard and it will help a lot for us.

**Is there anything else you would like to say that you feel you were not able to say during the interview? Any other messages or any other comments based on whatever discussed until now?**

I think whatever had been related to this thing is being done. My only thing is that this effort is very good. We should attack silicosis in an attempt to eliminate tuberculosis by 2030 or 2025 whatever the date is there. Like we did in HIV, we aggressively followed all those cases of HIV positive for tuberculosis and tuberculosis for HIV, so we succeeded in that. So maybe silicosis is an important problem and then very close association with tuberculosis and so it should be tackled as a priority otherwise we may not be succeeding because these pockets will remain and as I said mining employees lot of considerable population in that occupation. So we will be leaving a greater chunk of tuberculosis unattended so maybe that is to be addressed as a priority.

**In-depth interview 8: Expert in silicosis, 25 years of experience**

**India is one of the highest-burden countries for silicosis as well as tuberculosis. What is your opinion on the importance of addressing this dual burden for India, considering that both diseases are targeted for elimination by the year 2030?**

I think silicosis and tuberculosis always go in hand in hand. The reason is that silicosis predisposes to the development of tuberculosis and once the tuberculosis sets in the morbidity and mortality in silico-tuberculosis patients increase dramatically so this is important. If you see… in 1932, I think if I remember correctly, Gardner performed the autopsy of patients who died of silicosis, at that time even in the west, silicosis was fairly common and tuberculosis also and he found that almost 100% of the patients on autopsy he could culture the tubercle bacilli, so that is in advance silicosis almost 100% of the patients will get tuberculosis. It may or may not be revealed during the lifetime.

**We found in our study that silico-TB patients are at 2.3 times higher odds of adverse treatment outcomes as compared to TB patients without silicosis. What according to you are the reasons for these higher odds?**

The most important reason is that silico-tuberculosis patients are suffering from two diseases. Treat a patient for tuberculosis, you are curing only one aspect of that you are not curing the silicotic aspect. What will happen is that these symptoms will not go away fully. Second thing is that in tuberculosis the immunity is also reduced among patients with silicosis because basically, the primary clearing of tubercle bacilli is through the macrophages, and in case of this in presence of silica particles or quartz particles the macrophages cannot effectively clear tuberculosis so the susceptibility is increased. Third thing is that if you are treating a patient, I know this about the silicotic aspect… if you treat the patient, if you know the pathology of the disease, you know that there is dense fibrosis in silicosis. Now in fibrotic tissue, you know that the blood supply is very poor. When a drug is given orally or by injection it has to penetrate those, it has to reach the tissues where the tubercle bacilli are going but the drug may not reach there so the concentration of the drug is not effective and so the drug treatment is not effective. Now second thing is that in many of the studies they have found that in silicosis the presence of not only the tubercle bacilli but also the non-tubercle bacilli. You see e.g. mycobacterium kansasii or avium you know that they are not actually pathogenic but they also have been found to multiply freely in presence of silicosis and usually, they are resistant to most of the drugs so this leads to drug-resistant. Drug resistance is caused by the inadequate reaching of the drug to the site of the tubercle bacilli. Second thing is that tubercle bacilli are becoming resistant to the presence of the quartz dust and third thing is that the patient also does not comply because his all symptoms are not going away.

**What can be done for improving the treatment outcomes of silico-TB patients? What interventions can be planned for silico-TB patients so that their overall care and management be improved?**

The most important thing is that it starts with bringing all the stakeholders together. You see the stakeholders are the laborers, the owners, the government inspectors, the labor inspectors, and the people who are treated. You see all these people should have awareness about what is the disease and how it is clearly leading to tuberculosis development and how that specific treatment should be there. The first and foremost thing is that as soon as the patient with silicosis is there, further exposure should not be there, exposure to the silica particles, otherwise your treatment will fail. Second thing is that early diagnosis. Now early diagnosis of tuberculosis, early diagnosis is a very important thing but here you see the diagnosis can be done in two ways. You know the standard method is an X-ray chest otherwise a sputum examination you see for AFB, Acid-fast bacillus. Now, these two tests you see the standards are a little tricky here, normally you see tuberculosis usually in India any opacity on the lung you see chest X-ray not explained by any other thing will be treated as tuberculosis. But here you see it is very difficult that this may be masked. You see the X-ray picture is masked by the presence of silicosis so this X-ray diagnosis may not be definite. The thing is that since I have told you the regions are usually surrounded by dense fibrous tissues so there are no free communications with the bronchus. If there is no free communication with the bronchus then the bacilli will not come into the sputum and so it will be false negative. So this is the thing, diagnostic difficulty. I think there is one test and I suppose NIOH is also going to do it and that is a quantiferon TB gold test. Now, this test is very promising because another test is TST, I don’t know whether nowadays the demand for that test is there or not over time but in India, it is considered useless because most people are likely to be injected with BCG. BCG vaccination will be there so there will be false-positive tests. But this particular test I will tell you to see quantiferon TB gold test, I think is quite promising because it is not interfered with by the BCG vaccination.

**Can you suggest something on the treatments aspect, I mean how should silico-TB patients be managed clinically to improve treatment outcomes?**

Well, I think it doesn’t change much. One has to keep in mind the points I have told you earlier about the compliances, not their diagnosis. Why the patients are becoming resistant to the drugs? These points need to be kept in mind. One of the things for the prevention may be, I don’t know how far the people will agree but the preventive aspects of this thing are that you give the prophylactic anti-tubercular drug, these may be tried, I don’t know anybody has tried it or not. Particularly for high-risk patients e.g. somebody suffering from tuberculosis in the family or the surrounding like that he is a high-risk patient. The second is early diagnosis, I have already told you about that. Otherwise don’t think the silico-tuberculosis treatment will differ from the other tuberculosis patients. It will take a long time because the drug does not reach the site of the region and the likelihood of resistance is there. So I think multidrug treatment should be vigorously followed because you need more than standard treatment or not, I think that is the subject of research.

**What is your opinion on collaborative bi-directional activities between silicosis and tuberculosis?**

I think this is a good question. Tuberculosis, particularly you see silicosis is a disease that occurs in the particularly identified group is always there, you see this advantage. You go to the mines, and a large number of people you see in the mines are likely to suffer from silicosis and silico-tuberculosis. The other thing is that there is a high risk of this thing, you have already done surveys in the ceramics, agate industry, stone crushing, and stone grinding industries. All these industries wherever they are, there should be some collaboration, you see the people who are treating them, the occupational physician who are treating them, or the ESI doctors who treating them should be aware of that and they should have a sort of alliance with the national tuberculosis work group.

**Can you more specifically elaborate on what kind of alliance are we talking about, and how should they be communicating with the TB program?**

Well, I am very sorry but I don’t know whether I can answer this particular question because this has to be done at the highest level. The govt. policies come on the tuberculosis program, then the national tuberculosis program should include at least e.g. you have got 1.7 million people who are employed in various mines. Now mining industry goes with health departments so I am talking about higher level, lower level things that do not work. The inspector has got his work. The ESI doctor has got his work. So he doesn’t want to take extra botheration unless they are forced by the higher authorities and govt. officials.

**What would be the implementation mechanisms for bi-directional activities between silicosis and tuberculosis? Bi-directional activities essentially would mean that every patient with silicosis gets tested-treated for TB and every TB patient would get tested-managed for silicosis. How exactly can this be done? What would be the tests we can use for diagnosis of each disease and how would they be managed under programmatic settings?**

I don’t think every patient suffering from TB should be tested only history taking should be there and should not go beyond that because most of the tuberculosis patients you know in epidemiology what we call the attributable risk, the more likely patient you see than the other, attribute to this particular cause e.g. you see if you want to study say asbestosis you see the asbestos has got an effect on two types of cancer it causes you to know. One is it causes lung cancer and another one is pleural mesothelioma. Now in lung cancer, you see that cases are much larger but the attributable risk is much lower because most of the people who are suffering from lung cancer may not be exposed to or may not have any relevance with asbestosis. But in the case of rarer disease which we call it you see pleural mesothelioma, now in pleural mesothelioma, the attributable risk is more than half, maybe 0.5, which means more than half of the patients who are suffering from pleural mesothelioma are likely to be exposed to asbestos occupationally. So that is the difference. Here you see for tuberculosis, silica is not a significant attributable risk but for the silicotic patient, tuberculosis is an attributable risk. So every patient suffering from silicosis should be referred to tuberculosis but not vice versa, only history may be sufficient.

**What about high-burden silicosis areas e.g. we have pockets mostly in all states e.g. Khambhat in Gujarat, Vidisha in Madhya Pradesh, and Kolar mines in Karnataka, so we have these kinds of pockets in every state which are considered high-burden silicosis areas. would such bidirectional activities, I would say that testing every TB patient for silicosis make sense for these high-burden areas?**

Correct you here. When you say testing for silicosis, what test you are going to do? Every patient suffering from pulmonary tuberculosis is going to be X-rayed, there is nothing like any test which would say that he is suffering from silicosis. You did some tests but I am sorry but I am not convinced that those Clara tests or Clara cell tests and other things have real importance on that. You see which will tell you, yes there is a blood test when you do this particular blood test and it tells you he is suffering from silicosis.

**Dose the conventional X-ray which we use for the diagnosis of tuberculosis would also suffice for the diagnosis of silicosis?**

Every patient suffering from pulmonary tuberculosis is going to be X-rayed, there is nothing like any test which would say that he is suffering from silicosis. Yes, if combined with the history of exposure because in any occupational disease like silicosis you see an X-ray, you know is a two-dimensional picture, it’s just the shadow. The shadow can never be pathognomonic. So it has to be combined with the history of silica exposure, it is the most important thing for any occupational disease.

**After the diagnosis of silicosis, any suggestions on how they should be managed, or let’s say how they should be rehabilitated?**

I think very fundamental thought of a thing in occupational health you see, diagnose any disease, the first and foremost thing is that removing the person from further exposure, see that all other peoples who are his colleagues any one of them is having, measure the work environment and also have a policy of prevention, so all those are general guidelines specific for silicosis.

**Any experience of yours with supportive treatment like bronchodilators or oxygen therapy or steroids for patients?**

I don’t have any experience with that.

**Is there anything else you would like to say that you feel you were not able to say during the interview?**

Very exhaustive and congratulation on very nice questions I enjoyed talking to you.

**In-depth interview 9: Senior Professor of Pulmonary Medicine, 24 years of experience**

**India is one of the highest-burden countries for silicosis as well as tuberculosis. What is your opinion on the importance of addressing this dual burden for India, considering that both diseases are targeted for elimination by the year 2030?**

Yes, see in cure of tuberculosis only chemotherapy or only diet is not going to help, that is a well-known fact mentioned in all the standard textbooks and all studies. So now we need there is a need of the hour if you want to eliminate these diseases, to address these special groups of people having exposure to silicosis as a part of their occupation. See, we have to understand the situation that how and why these people are exposed to silicosis because they are going to silica-exposing industries for earning their bread and butter. So it is their occupational exposure occurring due to silica dust and ultimately they are suffering from silicosis. There are pockets in Gujarat and across India also where such kinds of industries are established, they are flourishing and they employ this kind of people. So we need to, first of all, find out from these particular pockets in Gujarat where these industries are flourishing and people go there to earn their bread and butter. So before implementing across Gujarat, I opine that we need to find out geographically the districts or the sub-district level where these kinds of clusters are existing and then we can move ahead. But definitely, we need to address these situations for the elimination of tuberculosis because if we do not address this then the purpose is not going to be served.

**We found in our study that silico-TB patients are at 2.3 times higher odds of adverse treatment outcomes as compared to TB patients without silicosis. What according to you are the reasons for these higher odds?**

Two things are coming to mind to this question, one is the person for whom is default or regular treatment failure because as I was telling they are working in such silica dust exposing industries, they are working in odd hours and that AKT is to be taken at a particular time once in 24 hours after breakfast and all that thing. Because they are laborers, they are concentrating on their work rather than the disease part or the therapy part so this is one opinion for that reason maybe they are not taking the treatment regularly as required as per guideline. So somehow their odd working hours or they are not able to maintain their 24-hour cycle of taking the AKT or the TB treatment I mean to say regularly. Then there can be transportation issues also because they are in one area and our national TB elimination program, we have DOT provider and all other assistants which are not nearby to their place, they are in remote areas and our health care centers, PHC and community health centers I mean secondary health care centers under our government are maybe far away so they are not able to approach them if there is any adverse reaction or hemoptysis they will stop AKT and ultimately it leads to an adverse outcome, this is the second thing. The third thing is tobacco because they all are laborers, they want to work for 12 hours, 15 hours, and 18 hours and for that, they want to keep themselves awake, for these, they are consuming lots of tobacco and caffeine-containing products. They abuse all these things tobacco and tobacco-related products, so maybe that is also one of the reasons. This is the third reason for the adverse outcome of the NTEP program.

**What can be done for improving the treatment outcomes of silico-TB patients? What interventions can be planned for silico-TB patients so that their overall care and management be improved?**

So as I mentioned the reasons, the answer to these reasons is that when we have to identify these pockets and then in our national program we know we have a system of DOT provider and everything but in pockets where factories are situated, we need to have like more patient-provider ratio, if there is one provider in thousand population, maybe we can have one provider for hundred population to take care of the regular supply of the drug and counseling, and for addressing the adverse drug reactions. See these adverse reactions are the most important point to be answered because anyways they are having the adverse reaction due to silicosis, silicosis makes them breathless, silicosis makes them have hemoptysis but they will think that this is because of AKT and they stop the drug. So the counseling and preparing of adverse drug reaction, addressing that at the nearest center and maybe where these industries are set up we can go to the industrial, admin part of the industries to have like a special concentration on these group regarding monthly or bimonthly checkup. We get symptomatic patients also, which can be screened, this is the second thing I can suggest. Maybe we can pick up them early and then give them early treatment. So maybe monthly or bi-monthly screening of all these persons who are exposed to silica dust can be done.

**Madam, you must be managing patients with silicosis and silico tuberculosis, we know that there are set guidelines for managing patients with tuberculosis. However, when it comes to silicosis, most of the PHC medical officers as well as the physicians don’t have any proper cure or maybe any other treatment modalities for silicosis. What is your experience with treating silicosis patients and how can we design a silicosis management guideline?**

To be very frank in the Bhavnagar district there are not many industries that expose persons to silica. So particularly in the Bhavnagar district, there are not many patients with silico-tuberculosis one thing. Whatever cases I see in the guidelines, which is again the same repetition of what I have told. See the treatment of tuberculosis is the same, AKT, the duration of the AKT, numbers of drugs we give as part of DSTB or DRTB regimen the drugs are same, the dosages are same, only we need to take care of additional symptoms caused by silicosis. Because silicosis will cause fibrosis, will cause hemoptysis, and all. As such there is no cure for silicosis, we have to stop the exposure and whatever damage to parenchyma is done due to the exposure leading to hemoptysis. So we need to give and form a guideline regarding the management of symptoms related to exposure to silica dust. Different guidelines and symptomatic management are the most important part when we manage a case of silico-tuberculosis. Sometimes most of the patients need oxygen supplementation so maybe there can be some portable van can be set up in these areas, if anybody becomes breathless or has [hemoptysis](https://www.google.com/search?biw=1536&bih=722&sxsrf=ALiCzsYssd-94oJIFUk7zitivb8W7Io4VQ:1655284439978&q=hemoptysis&spell=1&sa=X&ved=2ahUKEwjb-q-Ij6_4AhUwSmwGHRPACPYQkeECKAB6BAgCEDI). A van can just go rather than shifting the patient or calling the ambulance. Maybe there can be a mobile van ready available to address any of the symptoms occurring due to tuberculosis or silicosis and to treat them so that they have faith in the treatment because see now what is happening some patients having symptoms of breathlessness due to silicosis but what he or she will be seen that because I am taking treatment for TB, AKT and feeling breathlessness so I will not take AKT, so he would run away from therapy or regimen. So then maybe a mobile van can be set up in these areas for close monitoring and immediate answer to the adverse reaction. Patients do not have a problem in taking treatment, patient have a problem when their adverse reactions are not addressed properly and appropriately. So maybe some special van/ mobile unit can be set up to address these situations. This is feasible and not a very expensive solution in my opinion.

**What is your opinion on collaborative bi-directional activities between silicosis and tuberculosis?**

Some guidelines need to be sincerely followed. There are few guidelines in our program but as I told you, screening in these pockets where persons are exposed to silica is the only answer. Bidirectional as we do in anything, each patient with TB should be sent to X-ray for diagnosis of silicosis and each patient with silica exposure should be sent to tuberculosis because the symptoms are the same, X-ray findings are same, everything is same. Line listing as we do in HIV, every patient exposed to silica dust needs to be screened for tuberculosis. Unless you diagnose early, how will you… we will not treat. The infection will continue in the community.

**What would be the implementation mechanisms for bi-directional activities between silicosis and tuberculosis? Bi-directional activities essentially would mean that every patient with silicosis gets tested-treated for TB and every TB patient would get tested-managed for silicosis. How exactly can this be done? What would be the tests we can use for diagnosis of each disease and how would they be managed under programmatic settings?**

Not every patient of TB because the numbers of TB patients are huge in our state or in across India. Not all patients of TB are screened for silicosis because it is very difficult practically and very expensive thing. Because as I was telling… Bhavnagar district does not have any industry which is going to expose anybody to silica. There are pockets, particular industries in particular areas of Gujarat, where these silica factories or industries are there, where there is silica exposure occurring, so in these pockets only. Otherwise, if all patients with tuberculosis refer for silicosis then it will be a marathon and is very expensive also. Another way we can do but all TB patients cannot be screened for silicosis. Only in the exposed area, those patients who are exposed to silica can be then screened for tuberculosis because it is a predisposing factor.

**If we want to implement in high silicosis burden areas as you said, then what exactly are the tests which can use for the vice versa diagnosis of each disease?**

For pulmonary tuberculosis, 2 sputum with acid-fast bacilli sputum examination with CBNAAT whatever available and whatever feasible, so first and foremost is, of course, sputum examination and X-ray chest, will help in both.

**Is there anything else you would like to say that you feel you were not able to say during the interview?**

One thing I think I need to emphasize is regarding the, I mean this is very much technical but when you have asked this, I will mention, see when we treat tuberculosis, what is happening right now that when we treat tuberculosis, there will be a radiological improvement, of course, microbiological improvement will also occur but in silica what will happen that they will, in silico-tuberculosis the radiological improvement will not occur because silica will cause fibrosis, TB will also cause fibrosis, so there is extensive fibrosis even if you do that the radiological improvement will not occur. It is very difficult for a clinician to diagnose whether the fibrosis is due to tuberculosis or silicosis. Sometimes a patient will have MDR tuberculosis and we think that this is silicosis and we stop the AKT. So both the diseases give the same radiological pattern at least on X-ray. Of course, on CT and high-resolution images we can differentiate, if we talk about different level activities so these, I mean the differentiation would be difficult. So maybe a CT scan is a very costly affair, very difficult I know but if you ask for differentiation then it is always good to do a CT scan for diagnosis and follow-up also. Which routinely we don’t do under NTEP, we only do a follow-up at the end - only sputum and we prefer X-ray. Otherwise, we just end the treatment with some examination only. But if you want to do it for all silico-tuberculosis diagnoses, we have to do a follow-up with a CT scan. Because sometimes what is happening TB will be cured but silicosis will continue. Patients with silicosis will continue and patients’ lungs will be fibrosed due to silicosis, so we need to have close and stringent monitoring.

**Since they have silicosis, probably they would continue to have the radiological pattern that we normally see in a patient with silicosis, so in their follow-up are we looking at any clinical improvements while we follow up silico-TB patients? I mean since the patients would be having silicosis, the radiological findings may not alter much. So how do we manage their follow-up or do we look for clinical improvement during their follow-up?**

Yes, we look for clinical improvement, and then as I told you that we can do a CT scan, HRCT high-resolution CT scans can be done, which will differentiate radiological patterns between, radiological deformities caused by tuberculosis and silicosis.

**In-depth interview 10: Expert in silicosis, 33 years of experience**

**India is one of the highest-burden countries for silicosis as well as tuberculosis. What is your opinion on the importance of addressing this dual burden for India, considering that both diseases are targeted for elimination by the year 2030?**

See my impression of this thing is that these are two separate identities, silicosis separate identity and tuberculosis separate identity. Now they are associated with each other in a way that people who are working in silica exposed or any dust-exposed industry, are more prone to develop tuberculosis. In our experience also of conducting, directly dealing with the health surveillance of the people who are engaged in labor department or engaged with you know the construction industries and mining, we have in my opinion though I won’t able give exact figure there is almost people who are suffering from silicosis, more than 25 to 30% people have the associated radiological changes suggestive of tuberculosis, which may be understatement also but I would say a large number of people are having associated tuberculosis with the silicosis. Now despite being different entities altogether, silicosis predisposes to tuberculosis which is all known because silica being a very toxic material damages the pneumocytes and causes the predisposition to tuberculosis. The problem with tuberculosis, when it is associated with silicosis is that it has the worst prognosis both ways. One is it becomes much more severe, number two the treatment failure as you are also pointing out is much more and in my opinion, the reason is that the six months is not an adequate period for the treatment which is the standard for tuberculosis in case of person has associated silicosis or silicosis associated tuberculosis. This has been a general impression of people who are treating tuberculosis in silicotic patients that like earlier we had this 9 months or 10 months course which has now been reduced to 6 months. Simple tuberculosis patients will improve within 9 months or it becomes clear under the RNTCP program. People who have associated silicosis, took almost 2 to 2.5 years before they become negative, so that may be one of the reasons why we are observing the treatment failure in the case of people who have associated tuberculosis along with silicosis. The reason we generally give is that silicosis already is associated with fibrosis, the drugs have a delayed tendency of reaching the micro bacteria, or it doesn’t reach or doesn’t… you know kind of reaches late or whatever maybe the reason, doesn’t reach adequately, which is probably responsible for failure of treatment in these cases. I also agree and this is what the impression of people who are dealing with the RNTCP program is also because we have some people who are associated with the RNTCP program as well as with silicosis. This is my impression of the whole treatment failure cases. Another thing, one of the parameters which you have chosen is the death of the patient… now people who already have tuberculosis, established tuberculosis, established silicosis, have a greater tendency to go into the recurrent pneumothorax which is invariably in case of silicosis is the terminal complication. So these people do not respond to the treatment and die much earlier so if you have a patient which has started tuberculosis treatment late when he has already advanced silicosis and developed tuberculosis then the chances of successful treatment much less because they probably die not of tuberculosis but silicosis or complication of silicosis because they also have associated progressive massive fibrosis invariably treated who already have a very high category of silicosis. Already going to progressive massive fibrosis, then getting associated with tuberculosis or superadded infection of tuberculosis, so everything become so complicated, the chances of a person surviving are much less. So that is my impression of the whole, especially for the last six years I am dealing with silico-TB individuals. So this is what my impression would be in this regard.

**What can be done for improving the treatment outcomes of silico-TB patients? What interventions can be planned for silico-TB patients so that their overall care and management be improved?**

See, I think they need a closer follow-up, regular follow-up, as compared with patients with tuberculosis because they have more symptoms. They tend to switch over from one place to another place for treatment and because of symptoms associated with silicosis, they tend to flip from one doctor to another doctor which probably leads to the failure of the treatment. So much more convincing reason to be given to these patients like when I am dealing with these people of silicosis, especially I deal with the medical boards of silicosis, I tell them very clearly your silicosis part is never going to go and you will always have symptoms but the treatment part because of tuberculosis if you take a full treatment your symptoms will improve. So that convincing, you know the doctor has to convince the patient that the silicosis part is going to be there, nobody can help you out in that part, but the treatment of tuberculosis is important and he needs to take regular treatment because he will get some relief out of it. So that is I think one important in every treating physician has to emphasize to the patient. Much closer follow-up of these patients and longer follow-up, you see out of six months I would expect, you have already observed, a lot of people will fail treatment because of various factors, internal as well as external factors, so you require a longer follow-up, longer treatment. Then another thing that comes to my mind is that a lot of these patients continue to work in dust industries, unfortunately, they don’t have any option, so we have to convince them that they must come out of these kinds of industries, they must reduce the kind of exposure of silica dust. So these are some of the factors which we have to take into account while treating silico-tuberculosis patients.

**What is your opinion on collaborative bi-directional activities between silicosis and tuberculosis?**

You need a collaborative program for silicosis. I may not agree with every tuberculosis patient to be treated or be referred for an HIV test. I also do not agree with every tuberculosis patient needs to be evaluated for silicosis. But every case of treatment failure in tuberculosis must be investigated for silicosis because my impression is a large number of MDR may be having associated silicosis which prevents the treatment to be successful or which contributes to treatment failure. I would say every case of silicosis must be investigated for tuberculosis, that must be compulsory and every treatment failure of tuberculosis cases must be investigated for silicosis. Bidirectional would be important in these cases. It may not be that in every case of tuberculosis you need to investigate for silicosis. That could be important it needs evaluation.

**What would be the implementation mechanisms for bi-directional activities between silicosis and tuberculosis? Bi-directional activities essentially would mean that every patient with silicosis gets tested-treated for TB and every TB patient would get tested-managed for silicosis. How exactly can this be done? What would be the tests we can use for diagnosis of each disease and how would they be managed under programmatic settings?**

For every patient with silicosis who needs to be investigated for tuberculosis, the standards we already have for the test of tuberculosis must be conducted. Maybe every silicotic patient must have it because he is predisposed to tuberculosis. There may be hidden tuberculosis in silicosis but for every patient who is dealing with the, who already has tuberculosis when you wish to investigate for silicosis, I think in addition to a chest X-ray you must have the… because you sometimes tend to miss the early part of silicosis in cases with tuberculosis so maybe CT would be helpful. Need not have CT for every patient but for people with treatment failure or when you suspect but there is no evidence as such. X-ray because you would agree that many X-rays which we have in the field are not of very good quality, in fact very poor quality and they are not fit for evaluation as far as silicosis is concerned because of poor quality X-ray. So get a good quality X-ray done and then when you cannot find it or when you want to rule out further then maybe CT as required but I will not suggest that for every patient you must do a CT. That I don’t agree with. CT has to be used to exclude things, not for diagnostic purposes, which we normally recommend.

**From the field, we usually hear especially from CHC medical officers or district-level doctors that there are no set guidelines for managing silicosis patients. Any experience of yours on this?**

I completely agree with that issue. You see we have to, that’s what I have been suggesting to Rajasthan Govt. health department, we must have management guidelines for silicosis, which are just like you know what we have guidelines for tuberculosis. There must be, if no intervention, at the state level at least, but unfortunately the medical department does not take that thing very seriously despite that we have a very large number of cases of silicosis in Rajasthan and then we find that we still do not have the guidelines for standardizing certification, we do not have guidelines for standardizing diagnosis, we do not have guidelines for the standards of treatment. So every medical board in Rajasthan or every physician is left to himself how he decides, which is not preferable. So we must have standardized guidelines considering every stage of silicosis. When he has an early stage of silicosis when he has associated tuberculosis when he has higher categories of silicosis, when he develops progressive massive fibrosis, once he develops pneumothorax or recurrent pneumothorax and then sees somebody who is going into respiratory failure. So all these guidelines have to be prepared in detail and then circulated to every member, every person who is in the RNTCP program at least at the district level. So that would be my suggestion, definitely very much needed yes.

**We know that as such there is no definitive cure for patients with silicosis. What exactly do you suggest for managing patients with silicosis?**

I think educating the patient is important, especially in the early part, when he just has initiated but unfortunately, most of these people come to us when they have already in the advanced stage of tuberculosis, silicosis may be associated with tuberculosis. Using health surveillance, cases… you know… we can detect at earlier stages. I mean treatment guidelines, probably I am not the right person, I do not have direct clinical experience in dealing with the cases of silicosis but maybe one thing which is required very much is their rehabilitation with physiotherapy and occupation therapy part when they develop this dyspnea so that is because we have research projects also which we have ordered SMS medical college regarding the pulmonary aspect of the physiotherapy in patients who already have going repeated concurrent pneumothorax or advance silicosis. Exactly what kind of treatment will be required I am not the right person I would say.

**What is your opinion on dust control measures as far as improvement of outcomes of silico-TB patients is concerned?**

We all know one thing silicosis is caused by the excess amount of dust exposure and the occurrence of silicosis is directly related to the amount of dust inhaled. Now the problem is dust control, so the basic approach remains that the dust must be controlled at the workplace, to reduce exposure. The problem with the present setup which we are having is a majority of… as per our experience in Rajasthan… majority of cases of silicosis which are occurring are coming from small scale industries or small scale mines or mineral industry or stone quarry industry or you know that group. Small mines/ small industrialists are the major culprits in silicosis. Now the problem with these units is that they employ a very small amount of people maybe 5 people, 10 people, or 20 people which are outside the jurisdiction of any statutory authority. The amendment of the labor code now which you may be aware of, now the labor code or most of the laws will be applicable only when 50 or more persons are employed, now that you know… this kind of takes out nearly 95% of the units. So the problem is not that much in the case of the large units, it is small units that are the main culprit because they are outside the jurisdiction of most of the statutory agencies and even if they are within the jurisdiction of the statutory agencies, statutory tend to ignore them because that’s not their primary focus. So, like DGMS focuses mostly on the large mines… unless there is something major disaster or the same thing in factories. So most of these units are ignored as far as the enforcement of the law is concerned. Number two is that people at present because they are not covered in the jurisdiction of the Factories Act, they are not covered under the jurisdiction of the Mines Act., Now, who is going to enforce the safety and health of these people, nobody knows. Nobody knows in the state government. You go to the factory inspectorate; they say that this is not our jurisdiction, to whom do we catch? If you go to mines act, they say that the mine is not registered with me so I can’t do anything. Now, who is supposed to look after this? Most of the states, at least Rajasthan I know, don’t have any statutory cover for these aspects. So there have to be enforcement areas that will respond, who will enforce the provisions of the Mines Act, the provisions of the Factories Act, the provisions of the BOCW [Building and Other Construction Workers] Act, in these small-small units, which are at present totally out of any jurisdiction and that is where the majority of cases we have seen of silicosis are occurring. So that is another important thing, technology is very well available, peoples say let’s develop technology, sustainable development this and that but I do not agree with that because every technology is available, there is nothing great that needs to be done, the problem is the enforcement. Can we enforce people or people at workplaces to adopt technology, which may not be expensive at all, it is just the attitude of employer and employee, I do not fault the employer only, the employee is also equally at fault. So enforcement is a major part of and then enforcement of the people who are suffering from dust diseases, so, the law provides everything in that, there is no health surveillance of the people who are engaged, they have not registered also, even if they are registered with BOCW. Now we have constituted the mines welfare board in Rajasthan for the mine workers also. You see the health surveillance part is not an important part. Giving an example of the Rajasthan… Govt. has paid almost more than 500 crores as silicosis benefits to the people who are suffering from silicosis and thus we are giving out 100 crores every year in the pension and the benefits. But nobody is prepared to take up the health surveillance part… that health surveillance of people who are at least registered with us, which will hardly cost 5-10 crore rupees. So the health surveillance part is important and we are not focusing on that. That is, I suppose important. These are some of the factors in my experience I feel.

**Is there anything else you would like to say that you feel you were not able to say during the interview?**

I would like to point out a few things. In Rajasthan, we now have a state policy on the detection, prevention, relief, and rehabilitation of silico-TB patients which is very comprehensive and I would say well designed, and it is already implemented at the grass root level. We have an online registration system, online patient registration, online certification, and online benefit reimbursement, and the policy implementation is monitored at the highest level at the Chief Secretary level, at every three months there is a meeting, we have also given 5 crore rupees for the research to the various institutions now. So similar kind of approach or a similar kind of model must be developed by states, which have a high prevalence of silicosis such as Haryana, Rajasthan, Jharkhand, and West Bengal, these are four states already notified a policy. But the policy of West Bengal, Jharkhand, and Haryana is not as comprehensive as compared to the Rajasthan policy. So maybe they should adopt a similar kind of fully comprehensive approach with the implementation, which is monitored at the highest level. All these states are on the list, there are seven states which are on the list of silicosis considered endemic by the national human rights commission. The three I have already omitted are Gujarat, Madhya Pradesh and probably Andhra Pradesh they come into that group. In addition to that, you must have a, because this is not a problem of a few states, this is a national problem because there are many other states which have not taken any action, and silicosis cases are not detected and not reported because they have not done anything. See we have in Rajasthan, we have detected almost thirty thousand cases of silicosis because you have constituted a mechanism but the same may be true for all other states so you need to have a national program not only for silicosis but general pneumoconiosis program which includes likes coal mines are ignored all over like Jharkhand, Orissa, West Bengal, Chhattisgarh, MP there are very very large coal mines and their many people are suffering from coal worker’s pneumoconiosis. So you must have a national program that may be on similar lines as that of the tuberculosis elimination program then only some action will be able to take. Just throwing the… say that we have already created enforcement agencies like DGMS or we already have enforcement at the level of the chief inspectorate of factories, it doesn’t help at all. Silicosis is under a notifiable disease since the 1950s but look what has happened. Nothing. So a similar program is required, initially, you can have it as a part of the RNTCP but you must have a separate cell for silicosis. You see the general complaint that we are having in Rajasthan is that it is part of, the silicosis program so much money invested, so much, is still an adjunct to the tuberculosis program. Now tuberculosis people say that this is not our responsibility, we are just trying to help you out. So if you have a separate program, and separate funding then this would be an effective program and as far as eliminating silicosis in 2030, I think this is a typical dream, please forget about it, even the WHO and ILO have forgotten the whole exercise, they have done nothing in last 20 years, I can be very sure of it. Initially, the initiative was done by ILO, WHO, but nobody talks in ILO, WHO nowadays. So I don’t think it is possible. I know about tuberculosis but silicosis we don’t foresee as being eliminated, not in India, not in Rajasthan at least.

**In-depth interview 11: Regulatory agency official, 4 years of experience**

**India is one of the highest-burden countries for silicosis as well as tuberculosis. What is your opinion on the importance of addressing this dual burden for India, considering that both diseases are targeted for elimination by the year 2030?**

So because we have to address both the issues together because India is right now in the transition zone, transition zone in the sense that most of the people from the agriculture industries and some of the people are working in different industries also and definitely if we talk about the different industry so comparing to the work environment procedures, comparing to other areas or other countries, the dust or dust-related problems in India is comparatively much more. Those who are working in the industry are exposed to a greater amount of particles, most respirable particles in terms of their exposure. So definitely and along with those people are also living in some situations and due to the work-related policies, due to their working conditions they are also exposed to tuberculosis in their environment or their living environment so they are in both ways exposed to silica dust particle and along with tuberculosis due to the poor work environment and in their living situation also they are again exposed to tuberculosis. So they are in both ways, they have always been exposed to dust particle silica and along with tuberculosis so infective and non-infective risk factors are present in the working population and most of the working population are definitely in the age group between 20 to 50-60. So they are economically earning people/members in their family and they are the much more sufferer. So to increase the economic situation, and increase their industrial group, we have to address both issues together.

**We found in our study that silico-TB patients are at 2.3 times higher odds of adverse treatment outcomes as compared to TB patients without silicosis. What according to you are the reasons for these higher odds?**

One reason may be I suppose that is the main biological factor, that is because whenever a person is exposed to dust and the macrophages present in our alveoli they are much more working mainly to remove dust particles from the lungs so they are already overburdened, the macrophages are already in compromise stage to deal with the dust particles. So now whenever there is tuberculosis or they are infected with tuberculosis, their macrophage cannot effectively remove or effectively deal with the incoming bacteria so that’s why there may be adverse treatment outcomes compared to those who are not exposed to the dust particles. So those who are exposed to dust particles may have much higher odds of complications related to tuberculosis like different treatment failures and they have terminal outcomes also. This is the first one and the second one is the migratory nature of the persons who are much more exposed to silica. They are working mainly if you study the demographic profile of those workers, they usually stay in one state and for different silica-related work, slate industries, or quarry industries they migrate to another place for some specific period, work there and they again come back. So the compliance related to tuberculosis, if they have been detected there is one thing - late detection of the disease, both tuberculosis, and silicosis and along with that, another one is the continuity of their treatment. Whenever they are diagnosed due to the break in linkage so compliance is not so effective compared to those who are not exposed to silica and those who are not migratory people.

**What can be done for improving the treatment outcomes of silico-TB patients? What interventions can be planned for silico-TB patients so that their overall care and management be improved?**

One thing can be done that is the tracking system and specifically, we have to focus on the migratory workers, those who are working in that industry, and a robust tracking system is extremely important. So whenever a person is treated with tuberculosis and the suggestive of the working situation by occupation he is working in any kind of dusty environment. So from the very beginning very first day, he should be clear and a very efficient surveillance system must be, so only if there is any break in the linkage that should be addressed promptly. So that should not wait for some time, we should wait for another month and see if they come and give their report to another state or anything else so this type of thing should not happen. A clear-cut link mechanism, surveillance system, and robust monitoring system should be there.

**Any suggestions on how such a system can be established for tracking?**

Yes, one such system as per my experience I have seen that there is a very good system for tracking and monitoring the per se tuberculosis and the NTEP but the problem is whenever that person is coming in a factory, or in a factory which is even not registered. So due to problem-related with compensation and this purpose they sometimes move from that tracking system and they are in an isolated manner whenever they are suffering from any disease or they are isolated manner consult with different physicians or not in surveillance mechanism under NTEP. So the effective integration between the occupational health surveillance system and the NTEP should be there otherwise it is very difficult to track the patients specifically the silico tuberculosis patients. So a surveillance system, a robust surveillance system in occupational health and, occupational doesn’t mean only the registered factories that should include registered, unregistered factories. So a surveillance system and the NTEP surveillance system should be merged.

**What is your opinion on collaborative bi-directional activities between silicosis and tuberculosis?**

Yes, so that is the main issue. So we have to be approached in different ways and ultimately that should be merged to a fixed point. So like HIV and tuberculosis conjoint surveillance and the same way silicosis surveillance mechanism, what will happen silicosis surveillance mechanism is done by the ministry of labor or in the respective states it is done by the department of labor, and the tuberculosis NTEP is done by the department of health so that inter-departmental collaboration is extremely important, otherwise, it will not give us the optimal outcome. So the ministerial decision and ministerial collaboration and along with the implementation level also those who are implementing that program are in two different ministries that must be on a single platform.

**Which diagnostic techniques should be used when we diagnose TB among silicosis patients or silicosis among TB patients?**

Conventionally, to do the diagnosis of tuberculosis patients by sputum microscopy and polymerase chain reaction, CBNAAT could help and along with it… to diagnose silica patients… so one diagnostic approach is usually a confirmatory method usually advised for CT scan. So the first basic line will be the same as chest X-ray, sputum microscopy, and CBNAAT, definitely along with confirming or giving more accurate diagnostic results for silicosis, so those who are highly suspected of silicosis from the chest X-ray according to ILO classification so we can assume their gradings and their classifications. So those who have a high probability of silicosis along with the working history and the work environment data, so for that, we can go for HRCT.

**We understand that silica dust independently makes the person prone to the development of silicosis as well as independently makes them prone to the development of tuberculosis. Silicosis also has a higher probability of the development of tuberculosis. People say that it might make sense to diagnose every silicosis patient for tuberculosis and but the vice-vasa may not be exactly true for pan India level because when we talk about this kind of collaborative activities these are like the binding of all the districts, all the talukas or all the PHCs. So what is your opinion on, I mean should we diagnose silicosis among all TB patients or should there be any differentiation mechanism?**

In that situation for a cost-effective approach, we can give a targeted high-risk group approach, we can also do that thing. So we have to classify the tuberculosis patient according to their work profile. History and their working history are extremely important. So those who are working in a clean environment, not in a dusty environment, there are chances of, because dust usually silica is from crystalline silica that is the very important factor. We already know which industries are much more prone to develop that silicosis. The history only by a simple mechanism, taking appropriate history is extremely helpful to differentiate those who are at high risk of developing silico tuberculosis. So after taking the history if we can prioritize that work and then we can go for, in the next step, we can go for HRCT of them so then we can minimize the burden of that program.

**There were also talks about you know the small units, especially in the unorganized sectors, I mean people say that there are almost 90% of workers are in the unorganized sector and they are not under any factories act or any other statutory or regulatory body. What exactly can be done can you know some intervention which can be binding to these small units as well or mechanism to include them so that they reduce the dust exposure for the workers at small units as well, which we conventionally called as an unorganized sector?**

Yes, ok that is one issue, we have a definite schedule in the Factories Act. The upcoming occupational safety and welfare code, these are specifically addressing the problems in the organized sectors but yes in India many sectors, many establishments are in unorganized sectors. If it does not come under any specific statutes but definitely as per the state governments registration acts, they have to be registered under different activity, different acts, at least they come to know by the state government that… yes this is one factor which is not as a classical factory but it is an establishment where this type of activities is going on. So to track that thing, a tracking mechanism can be developed by the state government. These are the unorganized sector and these are the industries where tuberculosis and silicosis are occurring or single silicosis patients or developing the diseases much higher. By that mechanism, we can get at least baseline information, which are industries and their clusters and their locations so that we can prioritize or we can give emphasis on that particular district or particular talukas where such kinds of clusters are present because usually that silicosis or dusty environment where the chances of silicosis are much more are situated in cluster basis. So if we can identify the demographics of that special distribution of that clusters, we can easily track them.

**Which departments under state government are you suggesting should be given such responsibility or should take up this responsibility?**

One the department of labor and employment they have data related to that workers, migratory workers, employment, and unemployment, so they are the department, that should much more work on identifying those clusters and definitely along with the collaboration of your health department and another important department that should also help you is the department of environment because they have the data because as per the work environment monitoring you can get, they have the data where the dust exposure is much higher so you can also try to make a correlation between that three things and get a comprehensive result. So the three departments interlink between your environmental department, department of labor and department of health is extremely important.

**Do we have state-level bodies under the labor department to take into consideration all these?**

Already state-level departments usually sometimes depending whenever silicosis type of things come to their notice, usually notified by different NGOs, they are working in that field. Whenever the notice is sent to the state department, that complains goes to the ministry of labor, department of labor they usually form a committee to diagnose the case or to give a report on that. So apart from the ministry of health or the department of health in the state government, the department of labor also has some data related to silicosis and they also come to know where that type of silicosis patients are living or where the problem is much more higher.

**Any suggestions on how important dust control measures are as far as we are looking to eliminate silicosis from India?**

So definitely that occupational medicine or occupational health is essentially preventive medicine. Silicosis there is no such cure and yes the treatment is there but there is no such cure. But the only effective way to eliminate silicosis and silico-tuberculosis is through prevention. If we can minimize or clean the air from the dust so definitely that is the most effective way for the prevention of silicosis. So along with the dust and whenever that is not effectively possible so we have to use the other, so like usually called the hierarchy of controls so, elimination, substitution, engineering control, administrative control, and last but not the least is the PPEs. If we can enforce that hierarchy of controls in a very effective manner so we can almost, with certainty can tell that silicosis will not occur in the population.

**You mentioned certain management treatment guidelines for silicosis, we understand that there is no cure for silicosis but in general whenever we talk about PHC medical officers or even tertiary care level physicians they complain that there are no set guidelines as to how to manage patients with silicosis, so any ideas how can we come up with the such a silicosis management guidelines or any suggestions on treatment aspects of patients with silicosis?**

As such silicosis it is, I have already told that it is extremely, it is essentially preventive part but whenever that silicosis happens, so there are two to three things we have to keep in our mind that is the aggressive treatment for any infection so we usually in normal patients if there is an upper respiratory infection we usually do not prescribe antibiotics in first few days depending upon the situation, depending up on the progress of the disease, we usually take our decision but compare to that in case of silicosis patients we have to be very much aggressive from the treatment part because any infection that has their macrophages already compromised, so definitely we have to initiation of early initiation of antibiotics for treatment and definitely to give them relief so bronchodilator depending on their situation and yes steroid sometimes may be useful for the temporary relief of their problems. But yes there is no such, to date, there is no such definite treatment or definite medicine for the disease. So to be symptomatic and we have to try as much as possible the relief the patients and make their life as comfortable as possible and improve their physical quality of life index.

**Is there anything else you would like to say that you feel you were not able to say during the interview?**

Not such specific but yes I only one thing feel that there must be a way at the central level also and state level also because also there should be an integrated surveillance system like in our IDSP which is already present there. But there is no such integrated surveillance system in the case of occupational diseases. So we must have an integrated surveillance system so we should not be looking into the matter in a specific isolated manner like communicable, non-communicable, occupational, and non-occupational. So we have an integrated surveillance system so that we can encompass all that disease in a single platform. That is my last option or last opinion if I could help in that matter to develop the thing and to implement that thing in very near future, so I will be very happy to that.

**In-depth interview 12: Regulatory agency official (retired), 5 years of experience**

**India is one of the highest-burden countries for silicosis as well as tuberculosis. What is your opinion on the importance of addressing this dual burden for India, considering that both diseases are targeted for elimination by the year 2030?**

In my opinion, awareness of silicosis is very important. Awareness among the medical officers in that particular area as well as workers is very important and for that, we need specific training for medical officers in that area and also some campaigns for awareness among these workers also. It is very important. You see tuberculosis is also very common in India and because of silicosis, tuberculosis is very common because patients or workers who suffer from silicosis, are very susceptible to TB so it is very important to include both silicosis as well as tuberculosis simultaneously in the same category, silico-tuberculosis.

**We found in our study that silico-TB patients are at 2.3 times higher odds of adverse treatment outcomes as compared to TB patients without silicosis. What according to you are the reasons for these higher odds?**

As I told you that there is a lack of awareness among medical officers, particularly doctors who are working in that particular area. Second thing, because of lack of awareness, silico-tuberculosis patients are usually diagnosed with tuberculosis and because there is no response to the treatment, they are labeled as multi-drug resistant cases. Instead of silico-tuberculosis, they are usually diagnosed as multi-drug resistant tuberculosis only. So it is very important that the medical officers, workers, and staff at that point, they must be able to diagnose this as silico-tuberculosis, and not as TB alone.

**You mentioned that generating awareness would be one of the important interventions. Can you suggest any other interventions which can be planned for silico-TB patients so that overall care and management and treatment outcomes can be improved?**

As I told you, I was serving as the Director of Industrial Safety and Health, in this department, the medical officer in charge is usually posted on deputation from the health department and because they are posted from the health department they usually do not know about the causes of occupational diseases and because of these, they fail to diagnose silicosis in particular. So it is very important that the medical officers who are posted on deputation to this department, must be trained specifically for silicosis, only then they will be able to diagnose these cases. It is very important particularly for the medical officers of DISH as well as medical officers of ESIS, Employees State Insurance Scheme because ESIS is there in Anand, it is also there in Dahod and Godhra. So the medical officers of the ESIS will be able to diagnose these cases of silicosis. So the medical officers must be trained in the diagnosis of silicosis.

**What is your opinion on collaborative bi-directional activities between silicosis and tuberculosis? Could it be helpful for the final purpose of elimination of silicosis?**

Yes, it will be very much helpful. The collaborative study will be very much helpful.

**What would be the implementation mechanisms for bi-directional activities between silicosis and tuberculosis? Leading question: Bi-directional activities essentially would mean that every patient with silicosis gets tested-treated for TB and every TB patient would get tested-managed for silicosis. How exactly can this be done? What would be the tests we can use for diagnosis of each disease and how would they be managed under programmatic settings?**

Usually, the workers who are exposed to silica must be examined periodically. As per the Indians Factory Act 1948 also it is the statutory requirement for medical examination of workers who are exposed to silica. So it is very important, that every six months, these workers must be examined thoroughly, and if needed X-ray can be taken. If the medical officer feels that an X-ray is required, then an X-ray can be taken. But it is a statutory requirement.

**We say that nearly 70 to 80% of the entire workforce in India, are working in the unorganized sector and in the unorganized sector usually, we see that they may not undergo the statutory regulations of periodic medical examination every six-month, so what are your suggestions I would say to somehow include or cover these small units as well for the implementation of silicosis control as well as managing these patients through periodical medical examination?**

Even if these are small units, they are using quartz crushing stone and they are also using stones that contains more than 95% of silica, the Government of Gujarat by specific notification has included all these industries under the Indian Factories Act 1948, which is a specific provision for the government to include those industries where the hazardous processes are there, they can include under this Indians Factory Act, and accordingly, all implementations must be done.

**When we see its implementation, its implementation may not be up to the mark, I mean even in the organized sector we see that there may be some lapses in these periodical medical examinations. So what is your experience with both organized as well as unorganized sectors as far as the periodic medical examinations and the silica dust control measures are concerned?**

Yes, the organized industries usually they do carry out the implementation for the controls of the hazards particularly silica dust because it is a statutory requirement, so whenever the inspecting authority will visit that industry, they will say whether workers are exposed to silica or not, dust monitoring will also be done. So as per the Indian Factories Act, the dust in the environment must be measured particularly in organized industries. But in the unorganized industries, there is no such provision so it must be included under the Indian Factories Act and there also control of this dust or monitoring of the dust level must be done.

**What steps can be taken so that the unorganized sector also has to implement these provisions?**

Creating awareness among the workers as well as the owners of the industries because you see if the workers are healthy then all industries can produce more materials. So the workers must be healthy and for that purpose, the periodical medical examination and control of silica dust in the atmosphere is very important.

**Do you have any suggestions on how or what industries should do to control the level of silica dust in day-to-day operations?**

This is the subject of hygiene but you NIOH institute also has some machines for controlling the dust, particularly at Khambhat so that machine should be used. They are reasoning, they excuse that there is a power failure and some other reasons also but they must be explained how to use these machines particularly exhaust to control the dust level in the atmosphere.

**What according to you is the employer’s role, the factory owner’s role should be in silicosis control or elimination?**

The employer's role is to just do a periodical medical checkup, to motivate the workers for a medical checkup, and also at the same time control the dust exposure and provide effective PPEs, particularly for the silica dust/ respirable dust.

**We say that The Factory Act was established in 1948 but its almost so many years since The Factory Act has been established still we see that the burden of silicosis is more or less the same or has not come down to that level that we expect, even the silica dust level if we measure in factories they are way beyond the permissible level. So what according to you, I mean what is lacking actually?**

Implementation of the Act is very important and unawareness of silicosis is particularly. You see before many years silicosis was there in the Saurashtra region, particularly in Chotila and Savarkundla and other areas, at that time silicosis was diagnosed as TB because of unawareness, the same condition is here also because some of the medical officers do not know about silicosis, some private practitioner also does not know about the silicosis and usually, these silicosis patients are diagnosed as tuberculosis and because of that, they do not respond to treatment. They are diagnosed as multi-drug resistant cases.

**How this implementation through regulatory authorities be enforced and what are the other centers which come into play when we talk about silicosis control?**

For major action, you can arrange for mobile diagnostic measures, particularly a mobile van for the medical survey of the particular dense area where the silicosis is maximum. So periodically camps can be done for the diagnosis, diagnostic camps can be arranged, and because of that silicosis patients can be diagnosed very easily. Usually, workers, do not go for a medical checkup so workers must be motivated for medical checkups. Because of the unemployment also workers are in fear that if TB will be diagnosed or if silicosis will be diagnosed then they will be thrown out of the factory, so usually for that reason, the workers must be motivated for medical checkups.

**What should be the role of regulatory bodies like The Director of Industrial Safety & Health (DISH) or Hygiene or DGMS (Directorate General of Mines Safety), what should be their role, and how can they help in further better implementation of silicosis control?**

First of all, the doctors of the government ESIS, the Director of Industrial Safety & Health, and also the community health centers, must be trained for the diagnosis of silicosis, particularly silicosis and silico-tuberculosis. After that, they will be sent to the particular area where there are patients with silicosis or silico-tuberculosis, so it is very important the training the doctors also.

**Is there anything else you would like to say that you feel you were not able to say during the interview?**

If the workers are diagnosed with silico-tuberculosis, they must be given a specific health card, in this health card there will be all reports of the X-ray, PFT, and other things, also the treatment regimen, which type of treatment is to be given and date of the next visit. So they must be given a particular health card so that they can come to the center whenever they are called periodically. So health cards must be issued to all these workers who are diagnosed with silico-tuberculosis and they must be asked to visit the center on the next visit on a specific date for follow-ups.

**In-depth interview 13: State-level TB Program Manager, 7 years of experience**

**India is one of the highest-burden countries for silicosis as well as tuberculosis. What is your opinion on the importance of addressing this dual burden for India, considering that both diseases are targeted for elimination by the year 2030?**

Yes, our country has the highest burden of tuberculosis according to the latest national survey and previous surveys also. In that, it was found that Rajasthan is in second place for the highest TB-burden state in the country, but Gujarat is at the bottom place in this matter, which I have read in the study. The studies conducted for TB along with other comorbidities are mostly with other diseases like TB with diabetes, and TB with HIV, still, more focus is on such studies. More emphasis is given to malnutrition and pregnancy, but the occupational health part was untouched and was not focused more until this program and silicosis policy not implemented however some clinicians and experts were aware of this at their level or medical college level but the program part was not made. Such cases usually come with a TB burden but they did not get separate recognition like TB with silicosis. Luckily, I think some states of the country have done it mandatory to take the occupational history of TB patients under the NTEP program, the first time this has happened. By mentioning occupational health, even one column was inserted in the TB treatment card, and emphasis/ stress was given to mention the occupational history part like silica-exposed workers or mine workers, etc. and by this silicosis cases start to come out. Wherever such block exists like mining blocks or stone quarry industries, TB prevalence was found to be higher in such blocks as compared to other general populations. Such data found a higher prevalence of TB in such blocks because they have more TB due to silicosis.

**We found in our study that silico-TB patients are at 2.3 times higher odds of adverse treatment outcomes as compared to TB patients without silicosis. What according to you are the reasons for these higher odds?**

Yes, definitely people who are suffering from silicosis have higher chances of TB as compared to the general population, even it is found according to you also, I cannot mention exact data but it is there 2 to 3 times higher, even somewhere it may be 5 times higher also. The reasons are due to their lower literacy rate, they do not follow the health & safety measures properly, the major reason is that even if we teach them, awareness among them will not come, they even think that this disease will not occur to us, their health-seeking behavior is not good, they do not approach or visit nearby PHCs on time, taking self-medication. One more reason is that they presume the symptoms of silicosis as those of TB. I am telling both the reasons why there is a late diagnosis of silicosis and why there is a late diagnosis of TB. During the silicosis symptoms, they thought that these might be TB symptoms and they were diagnosed with TB. After the development of silicosis in these people, recurrent TB occurs among them because the lesions permanently stay on the X-ray and do not go away. If they will not go to experts then they will be repeatedly treated for TB each time they visit. So they and the people surrounding them think that it is TB and are inadequately treated. There is more self-medication among them because they think that the same medicines are prescribed each time so they and their colleagues also take the same medicine without consulting. So later TB will become resistant among them and they suffer from serious TB symptoms. These are some reasons and one more reason is if one of their colleagues suffers from TB or silico-TB and they are sitting together and since they are not using any mask or any etiquettes or TB infection control measures, they neither adopt them at the workplace nor at home. So they pass on the TB infection to each other at the workplace. We found that if one suffers from TB then many others will also be diagnosed at the workplace or his brother or other family members will also be diagnosed and this will continuously exist among them or this cycle will go on. These are some reasons, other established reasons are already there like malnutrition and smoking these are add-on factors among laborers of silicosis victims.

**What can be done for improving the treatment outcomes of silico-TB patients?**

General common things as we suggest to all like first and foremost is awareness, there are guidelines at international level also, as TB disease is since centuries, first of all, they have to think about does these symptoms are of TB? They should be aware of the symptoms of TB and they should know where to consult or test if such symptoms happen to them, so the first thing is thinking /knowing about the symptoms. If he is aware of this, then only he will think about it. They will learn/know about the symptoms of TB from IEC, awareness programs, community participation, everywhere some display about silico-TB awareness, information through miking system or any other IEC medium. So they will think/know about the symptoms of TB and it will occur more in silicosis disease. So first, they will think about it, if such symptoms happen then what needs to be done further, which tests needs to be done for TB, and where this test should be done, these tests can be done free of cost at govt. centers, TB tests, the test of cough and sputum, about other tests like CBNAAT. So they should at least know that they have to do a cough/sputum test at a nearby govt. center free of cost. Once he consults a medical officer then his future path of treatment will be easier. Thirdly, if he suffers from this then he will get proper treatment. Treatment is available at all places through the DOT medium. As CBNAAT is mandatory now and it is facilitated by govt., silicosis patients with TB should be tested earlier with CBNAAT, as sometimes it happens that sputum turns negative or fewer bacilli discharge from lesions present in the lungs so they should get early benefits from CBNAAT due to fewer bacilli burden. Now program officers or medical officers working at the community level should send the samples for CBNAAT to make the diagnosis early and easier. Once they reach the medical officer then all treatments will be completed. Treatment adherence is much required because the lungs of these people are weaker. To prevent default [treatment interruptions], the first four weeks of patient treatment is very important, during such a period, our selected health workers at health centers or urban areas where he resides or at DOT center must be sensitized that this is not a simple TB patient but this is silico-TB patient, such patients will have more problems, they will have breathing problems and many other problems as their lungs are damaged, so they should not stop medicine of TB, so frequent counseling of such patients will be required. The health worker needs to be in contact with the medical officer and patient and should act as a link between them, so that patient will take all the prescribed medicines and complete the treatment course.

**What is your opinion on collaborative bi-directional activities between silicosis and tuberculosis?**

Currently, I am working in Rajasthan, the bi-directional activities are going very well here. One department is looking at both things; the TB department is looking after both the diseases, this is benefited more, and linkage is established. The benefits started when we started to take the occupational health history. This point is discussed in every review meeting. Due to this awareness, more concentration is being given to silicosis patients. Wherever silicosis activity is going on, we also provide information about TB and wherever TB activity is going on, we provide information about comorbid conditions and high-risk silicosis groups and establish linkages. So I think here in Rajasthan we have taken up this program in a better way and we have implemented silicosis policy/ guideline well and bidirectional activity is going on very well.

**Do you have a silicosis policy there?**

Yes, luckily I think Rajasthan might be the first state in the country where a silicosis policy is implemented. The silicosis policy was implemented here in October 2019. Before that also we do have other programs for silicosis, since 2015. Pneumoconiosis medical board was established here, wherein silicosis diagnosis program and training were there, emphasis given on early detection of silicosis so that something can be done for rehabilitation. Then after that, the silicosis policy was implemented.

**What would be the implementation mechanisms for bi-directional activities between silicosis and tuberculosis? Leading question: Bi-directional activities essentially would mean that every patient with silicosis gets tested-treated for TB and every TB patient would get tested-managed for silicosis. How exactly can this be done? What would be the tests we can use for diagnosis of each disease and how would they be managed under programmatic settings?**

As I have told you, by taking occupational history everything will become clear. If the occupation of TB patients does not correspond to silica dust-generating work or the mining industry, then it is ruled out. If there is no occupational history then it rules out there itself.

**What will be a diagnostic test for silicosis in TB patients?**

For diagnosis of silicosis or silico-TB, we are using a chest X-ray. If there is occupational history then we send that patient for a chest X-ray. Training for ILO grading is provided here to medical officers. All medical officers are aware of ILO grading, it’s not like every time they are doing ILO grading but after two to four times go through of that they are sensitized enough to identify that these might be suspect cases of silicosis, then they send it to experts - MD medicine or radiologist - then expert confirms the cases of silicosis. These all are part of the silicosis policy. The confirmation is done by the medical board, where three people were there. Recently Govt. of Rajasthan made some changes, the whole program is made online, and an online database is also created. If doctors at PHC or CHC level do not understand the X-ray then they upload it online, it reaches experts online, and an expert will provide their comments. Such type of program recently came here in Rajasthan. This remains a diagnostic tool, even if there is doubt and if the patient is symptomatic & TB is ruled out with all the tests that there is no TB, then in such cases with experts opinion we also do patients CT scan & HRCT thorax.

**If we diagnose silicosis, then how can we manage those patients? I mean what can be done about treatments or pulmonary rehabilitation part?**

There is no definitive treatment for such patients but we do the symptomatic treatment based on the condition of the patients like their respiratory difficulties, breathlessness, and general complaints. We always do their sputum test every three months or six months or whenever there are TB symptoms as it is a must to rule out the TB because they are more prone to develop TB. Otherwise, treatment will be based on their symptoms only. Sometimes they require more oxygen & every symptomatic treatment is followed. Until now there was nothing done for rehabilitation because rehabilitation centers were not available everywhere except for some selected places, but recently Govt. of Rajasthan has put this point in silicosis policy for rehabilitation. So medical college level or center of excellence are going to be established at selected three or four places of Rajasthan where such types of problems are more, at those places, such types of rehabilitation centers will be established, where patient’s rehabilitation get completed through multi-system approach by whatever means like yoga, physiotherapy, oxygen therapy, etc.

**Is there anything else you would like to say that you feel you were not able to say during the interview?**

Yes, one thing is there, under the rehabilitation part government has started giving them the pension facility here because the government came to know that most of such people will not able to do any type of work in future so they started a pension scheme for these people, right now there is fix pension of approx. Rs 2500/- to Rs 3000/-. The second thing I know is that people connected with ESIC will also get a pension based on disability percentage under the disablement category from ESIC. This is for rehabilitation. The other thing I want to mention here is that the emphasis should be given to widespread prevention activity so that these people can understand it, because these people do not understand this so NGOs or govt. should go to these communities and make them aware to start the preventive activity. As these people are suffered from silicosis within 5 to 10 years and their life end in 15 years, as the life span of these people is shorter, you at NIOH have also seen this in retrospective studies, so we have to stop this because after illness we can’t do anything. Whatever investment needs to be done in rehabilitation or other things, part of the investment should be done in prevention activity so people comfortably continue their life cycle, able to continue their livelihood, and also be able to avoid disease. This is what I want to say.

**In-depth interview 14: Industrial hygienist, 30 years of experience**

**As this study participant had expertise in industrial hygiene, excerpts from the interview include only responses on his expertise. He did not have any comment on the rest of the questions.**

My specialization is in industrial hygiene. I had worked on the industrial hygiene part of silicosis. The medical part was looked at by other scientists & industrial hygiene part was done by me, then we correlate the medical findings and the industrial hygiene findings.

I usually look into the exposure level of free silica particles, how many times it is more than TLV, and if it is higher, then how can it be controlled? For that, we have studied the exhaust system setup and all other measures.

The thought behind the study was that there is no treatment for silicosis so we aimed to minimize the exposure level at the source and bring it down to less than the permissible limit so that workers would not have health risks. For that, we have developed the local exhaust system and installed it at 15 to 20 industries and done the experiments and we found a 90% reduction, such type of work we have done. Silicosis prevalence comes down. That was a dry process.

But in Jodhpur/Jaipur there was the wet process. If you work in the wet process, whatever you want to work upon, then that will be important because there you will see very less prevalence as compared to what you will see here at Agate industries in Khambhat. Because in Khambhat, it is a dry process, it is in direct inhalation. But in Jodhpur/Jaipur there is no direct inhalation because of the wet process, most of the dust which is coming out would be settled at the source itself.

We tried to implement such a wet process in Khambhat but some type of specific work is not possible with this wet process so these people have to go with the dry process and because of that the morbidity was much higher over here as compared to the Jodhpur/Jaipur area.

There were two solutions at which we were working, one is to go at the source level, like these people making pearls & other things from these stones. First, we tried to eliminate this whole process, particularly this stonework, and tried to provide them with other alternative processes but we could not do it because such types of stones are very cheaply available in our places, also available cheaply at mines. We tried a lot but could not find the alternative composition, we tried a lot from an R&D point of view, but we could not get it. So we have thought to reduce the exposure at the source level, we tried to pick the dust aerosols and get them out through the exhaust system so that it does not come into the inhalation process when the worker works. We have done such types of works which are also available at institutes library.

We used to measure the dust level at any such place like, what is the level of dust, how much it is more than TLV, and what changes can be done to reduce the dust exposure because unfortunately, we don’t have treatment part for it all over the world, so the engineering control is the only option. Industrial hygiene is a very important portion of occupational health, as it tries to minimize the exposure at the source level or the air path level, PPEs come afterward. The less you do such a part, the less will be the health risk impact part. So we do such type of control technology work.

We do a lot of work on this but unfortunately, we do not work more on the legal aspect, as the factory inspector takes a bribe and subsides this disease, he has to notify this disease, this was a major problem we noticed in this country. When such a worker dies due to silicosis, they should get compensation but at that time they did not get it, but now they get some compensation due to the involvement of NGOs.

Another difficulty we found is that most young people are engaged in such occupations. They are only concerned about their bread and butter, not their lives. Our priority is life first but their priorities are opposite. They work on a per piece basis, so the more they do more they earn, and they spent this money on addiction & other wrong practices. We did a lot of work but R&D has no meaning unless it gets implemented. In foreign countries, they ban such activity immediately. But if we ban them here, then what about their job? Where do you substitute such people?

We have suggested one idea also to set up industrial estate near these villages where all such small units can be set up and we tried to set up one exhaust system in the middle of all these units so that all exhaust material comes at the middle, then this solid material can be used in ceramic wares for making cup and saucers, for this, we also have done the experiment and we were very successful. The collected dust can be used in pottery where it can be used as raw material for making cups and saucers. You see we have given a full solution to that.

There are multiple quartz crushing places in Godhra also, where silicosis cases are there but reduced compared to earlier times because they implemented different things like exhaust systems developed there. Installing a local exhaust ventilation system at every place to reduce dust exposure.

There in Jaipur/Jodhpur, people make beautiful pearls and all other things, but there is a wet process, so less rate of silicosis. Rajasthan is a stone state but the exposure is less. But here due to the dry process, exposure is more, so the level is much more than TLV.

**In-depth interview 15: Expert in silicosis, 40 years of experience**

**India is one of the highest-burden countries for silicosis as well as tuberculosis. What is your opinion on the importance of addressing this dual burden for India, considering that both diseases are targeted for elimination by the year 2030?**

It is more important than anything else, I am saying it is the most important challenge and it is a most important priority that we should have because when it comes to occupational health, in India maximum mortality as well as morbidity is with occupational respiratory diseases in which pneumoconiosis is number one, and most of the pneumoconiosis is silicosis, of course, there are cases of asbestosis, siderosis, coal worker pneumonia others, but silicosis by far is most important numerically as well as severity wise. You may be aware that recently the Govt. of Rajasthan has compensated more than 15,000 workers for silicosis. I mean they were the mine workers, who were suffering from silicosis so the Govt. decided to pay them some ex gratia and compensation, so it has been paid to more than 15000 people. Now, these are the official numbers within the last few years in one single state of Rajasthan, so from this, we can imagine what will be the severity and what will be the quantity across the country. Therefore, silicosis is the single most important occupational health challenge in our country, that must be tackled with all priority. The second thing - silicosis and tuberculosis - both diseases affect the lungs and both diseases complement each other in damaging the health because there is complementary action and that’s why silico-tuberculosis will have a much higher prognosis, worst prognosis than tuberculosis alone. Second thing, tuberculosis can be treated because it is a bacterial infection but for silicosis, there is no treatment, there is no way we can treat it, we can only mitigate their symptoms, we can only improve their quality of life, but because it is a progressive disease, it is a matter of time.

**We found in our study that silico-TB patients are at 2.3 times higher odds of adverse treatment outcomes as compared to TB patients without silicosis. What according to you are the reasons for these higher odds?**

If anything, it could be an underestimate. The actual morbidity or even mortality may be even higher because it is not easy to reach them. See it's only in the organizations, institutions, and tertiary care centers that we get to know these people and we do their follow-up. Because for tuberculosis at least we have ways of diagnosis but for silicosis, the diagnosis is not easy. What we learned from the Rajasthan experience also that silicosis diagnosis is very difficult because any GP can not diagnose it, even pulmonary physicians also find it difficult, you need certain sets, certain specific things even the ILO radiographs, etc. for prognosis and monitoring the patients. Such information and knowledge are not available across the country, therefore, it is not very easy practically. Now the reasons are I personally feel its silicosis which is much severe disease, which is not a treatable disease, and a disease which is continuously developing and worsening, because once an individual suffers from silicosis there is nothing that you can do about the treatment of the disease, you can only mitigate the symptoms, you can only help in the quality of life for some time. That is perhaps the reason, it is the silicosis part of it that makes things worse, whereas the tuberculosis part of it can be controlled with the help of effective medication which is now available, whereas for silicosis part that thing is not available, and even if tuberculosis is treated with treatment, we may still find that the silicosis remains, and that continues to affects the condition of the patient, the health of the patient. That’s why even if you give him the full treatment of tuberculosis, at the end of it the person still recurs that what it was before, despite successful treatment. Therefore, the outcome is going to be worse. So that’s why without undermining the importance of damage caused by tuberculosis, I will say the damage which is caused by silicosis is much worse and irreversible, so that is the reason for this higher morbidity and mortality in silico-tuberculosis patients in my opinion.

**What can be done for improving the treatment outcomes of silico-TB patients?**

First and foremost, would be diagnosis and catching the cases of silicosis as early as possible, I mean what is more important is the prevention of silicosis, because silicosis is something that cannot be treated, therefore it needs to be prevented, that we all know very well. So that should be the primary step but despite that and for that reason we have to educate the employers, the supervisory people, the common people, including the workers who may be working in susceptible occupations, the susceptible industry, because of what we see is in the organized industry the prevalence of silicosis is very low, but whereas in most of the cases that we have got are from unorganized sectors and that’s why the unorganized or small scale or those who are not very large enterprises, so these are the areas where we need to focus. We need to focus on the prevention of silicosis, we need to focus on proper ways of doing their business, especially manufacturing, mining and whatever is required, that is the primary thing because treatment wise there is very little which is possible. The only thing that we could do is if we diagnose the disease early then probably we will know what our priorities are, and we can give a longer life and better quality of life to people who suffer from silicosis for a longer period. Though whatever we do once a person develops silicosis, we are not able to do much but we can delay the deterioration in the condition and make the person rehabilitate, I mean help in rehabilitation and doing other activities by stopping his exposure because the key would be to stop the exposure and people may even go back and do the same work again even after the diagnosis, then their thing gets worse. That’s why the remedy would be not on the treatment part but it would be on the early detection as well as prevention part.

**Can you elaborate more on how can we go for early detection as well as prevention for silicosis?**

Early detection could be as I said, first and foremost educating the people, making them aware, see if people do not know, if the supervisors, managers, and owners, if do not know that silicosis exists and what is the severity of it, then it will be very difficult for them to even suspect it, that’s why wherever the vulnerable situations are there, these are the places where we need to reach and inform them and educate them about this disease, symptoms, and possibilities. So that has to be done with all the priorities, unless we do that rest all other things will be academic and they really will not give us many benefits, so this has to be on priority. Then early diagnosis before much of the damage occurs, if we diagnose these patients we remove them from exposure as early as possible then certainly the treatment outcomes will improve, and the longevity of such patients will increase. For early diagnosis, I have said their awareness, frequent examinations, and regular medical checkups, these things will certainly help. Currently, the diagnosis of silicosis is by radiology so I do not know what the utility of the serological test is which has been developed. But if those tests are really useful then mass-scale deployment of these tests will also helpful. If such a serological test is not there currently then we look forward to NIOH continuing their research and coming out with such a test or a battery of tests. I know that some of the tests could be common for other respiratory diseases also but then we will have to at least be able to shortlist some people and investigate them in detail. The main factor here would be that since these people are in the unorganized sector or very small industries where the management is not enlightened and they are not willing to spend money or especially before the person shows any signs and symptoms they cannot spend money on this that is the biggest challenge.

**You also mentioned the rehabilitation of the patients, can you also elaborate more, whether are we talking about vocational rehabilitation or pulmonary rehabilitation, and also when we say that improving their quality of life even after they develop silicosis, so any experience of yours using any of the medicines or drugs for improving their quality of life?**

Vocational rehabilitation cannot be done unless we do pulmonary rehabilitation because it’s an integral part. Unless we do pulmonary rehabilitation, the patient is not comfortable so there is no way he is going to work anywhere, that’s why that has to be there, I mean they are not mutually exclusive, they have to be done together. Now vocational rehabilitation will come when we diagnose the disease early enough, when the person has still some energy left and capacity left to work, and here our objective would be to stop further exposure to silica dust. So that’s why vocational rehabilitation should come even before the early signs and symptoms of silicosis are seen. Just by the history and by the environmental monitoring if we know that in this particular situation and these individuals are prone and they should be removed as early as possible and they should be given alternate work, that is the vocational rehabilitation, so that will have to be done. So vocational rehabilitation should start early before even people develop symptoms. Now, this is difficult to implement in practice. Easier for me to tell you but in practice, it’s going to be difficult, I understand that. But then that is where the might of government and regulatory agencies should come in to picture because mines are also coming under various acts and now we are aggregating all that into a single labor code. So both rehabilitations are important.

I am associated with silicosis work and rehabilitation work and some things which have been done in the state of Rajasthan. There we have seen that even giving portable oxygen cylinders and other things are helping the people. Giving them some respiratory physiology, some training, and Yoga, not exactly Yoga but whatever you can say some exercises, they certainly help them. Of course what is happening is most of the silicosis that we know comes in terminal stages, after they are unable to work so in such cases it is more difficult. If we can diagnose the disease early then perhaps we will have better experience in answering what you have asked. But the treatment of course would be supportive, there are no specific drugs, of course, I am not talking about tuberculosis because that is all well known, but apart from tuberculosis, improving their diet, giving them respiratory exercises, and other things, taking care of their other requirements that would help them to live a relatively longer life than what it was before, that is an experience in Rajasthan. But still the situation there also is, I won’t say that we have come a long way it's only giving compensation but the focus is yet to go on the prevention.

**What is your opinion on collaborative bi-directional activities between silicosis and tuberculosis?**

I think this is the most important thing, it is very very important and in my experience or in my opinion or my perception I would say each case of silicosis is a potential case of tuberculosis because lungs are damaged, their socioeconomic condition is much worse, their income stops and the area they live in and you look at all other conditions… many factors which promote tuberculosis are already there, then the hemoglobin also reduces, body resistance reduces and as you know TB is an opportunistic infection because most of us are Mantoux positive, the organism is around us. So, every case of silicosis is a potential case of tuberculosis. Second, most of the cases of silicosis are diagnosed as tuberculosis, I mean, are misdiagnosed as tuberculosis, I will put it this way. Because of the clinical symptoms and other respiratory infections, it leads people to believe then treating people also, the doctors and others, find it very convenient to label the patient as tuberculosis, rather than labeling silicosis for which you need a lot of proof and a lot of conviction, which is not, the facilities may not be easily available at PHC level so therefore they get misdiagnose and mislabeled as tuberculosis anytime even though they do not suffer from tuberculosis at that point of time and therefore they may not come bacteriologically positive. But after some time because of their situation, they develop a secondary infection or they develop the infection with tuberculosis. Silicosis patient who is negative today, after a couple of years will become Koch's positive, this is what we have seen. So therefore given that silicosis may be misdiagnosed or confused with tuberculosis, silicosis patients are very susceptible to tuberculosis, the presence of tuberculosis infection in society, in the community, I feel, I mean nothing can be more important than this bidirectional activity between tuberculosis and silicosis. Both these programs should and must work together, in fact here I will also like to tell you, that I also have some international exposure because I am active in the international commission on occupational health, so there also there has been an international forum and there has been a lot of discussions and all. So initially the silicosis activities, silicosis treatment, and detection and all these activities are focused on as independently, but subsequently internationally also it was learned that it will help and it will aid if we combine tuberculosis and silicosis, then we will be able to diagnose or find out, detect more cases of silicosis and treat them effectively. So it is not only in India but international wisdom also says that both have to coordinate and collaborate and bidirectional activities should be taken up in the right direction.

**You mentioned that each patient of silicosis should be diagnosed with tuberculosis and is a potential case for tuberculosis, what will be the other way around, I mean?**

Yes, tuberculosis patients, those who are suffering from or those who have suffered from and as a result their lungs are and their respiratory capacity is weakened will be definitely at higher risk if they are exposed to dust. So if tuberculosis patients are exposed to dust and such vulnerable occupations where silicosis is a possibility then definitely they will suffer silicosis faster than other normal individuals.

**So you suggest that every patient with tuberculosis should also be diagnosed with silicosis?**

Here the occupational history is more important because in the case of silicosis there will be a definite occupational history. Generally, silicosis won’t happen with the, I mean to say if you are staying in the house, near a highway or you are near a flyover and therefore you get more dust and therefore you develop silicosis, that is very very rare. But to develop silicosis, you have to have exposure to dust and exposure to a reasonable amount or reasonable quantity of dust. Therefore, it cannot be missed if a proper history is taken. So that’s why history taking is very important and wherever the person gives a history of exposure to dust, silicotic dust, or such occupations, then we should screen those persons for silicosis. Similarly, if persons with a history of tuberculosis also if they are working in such occupations where they are prone to silicosis, they should be screened for silicosis.

**For tuberculosis, we already have a national program and very well-defined algorithms as well as criteria for diagnosis. For silicosis what do you suggest suppose that among patients with occupational history and get diagnosed with tuberculosis, if we want to diagnose silicosis among these patients, what would be the better method from the point of view of public health? For diagnosing tuberculosis silicosis there are established methods, but for diagnosing silicosis among tuberculosis patients, what would be the best method or better method if we want to implement such bidirectional activities?**

As far as diagnosis of silicosis is concerned, currently, we do not have any methods other than radiology, because it’s not a microbiological disease, so, therefore, there are no such test which is available where you can detect an organism or that kind of screening is not possible, the only screening which is available for silicosis right now to my knowledge is X-rays (radiology) and radiology also should be on the appropriate machine, because if you do 60mA machine, if you use it, it may be not able to diagnose but of course now that question is less because now we have better radiological equipment available which is also at primary health level things may be improved. So apart from radiology, yes as I said if some serological test is developed, if they really come out and if they have acceptable sensitivity and specificity then they will become our first-choice screening test.

**Under the programmatic settings i.e., if we talk about the public health aspect of implementing such bidirectional activities, we would require diagnostic methods at the PHC level as well for diagnosing silicosis among tuberculosis patients with an occupational history. How do you suggest exactly what can be done more in the way that I mean what kind of machines, digital X-ray machines would require with how much resolution at the PHC level so that they will be able to differentiate easily between silicosis and tuberculosis?**

I think for that purpose we have laid down guidelines by ILO and that should be followed. This is not something that we should leave at the discretion of the doctors or based on their experience. It should not be a subjective thing; it has to be an objective thing. Whatever guidelines, ILO has specified everything, and now ILO has come out even with a digital set of X-rays, and ILO is conducting repeated training for that, ILO radiography for silicosis, so that capacity building has to be done. Then suspicion of silicosis should be there and these things can be developed at maybe district places first and then at a place where there are more people of this nature. So depending on the exposed population the prevalence or presence of the exposed population and their numbers, the centers which can diagnose can be increased in those places where there is high prevalence or where there is a high likelihood, we can have better X-ray machines even at PHC level; where there is low, we can have them at the district level and patients can be referred there, like that we can do it.

**We have a TB elimination program, and on similar lines usually, we get complaints from PHC doctors as well as from doctors even at district hospitals that there is no silicosis elimination program so what is your opinion on the need for such defined programmatic guidelines for silicosis management or rehabilitation?**

Yes, the need is there, we need to have a silicosis program but unfortunately, we cannot have a silicosis treatment program, will have to have a silicosis prevention program and that is one. Second yes we can, now that there are more and more cases, see the problem with silicosis is people are not willing to report even cases of silicosis. But now that the cases are being diagnosed, more and more cases as we have seen in the court have also intervened, the human rights commission has also intervened, we are seeing more silicosis cases, now we have more experience, so we should get this information together. What are the experiences at various places, collate them together or maybe conduct different studies I am sure there will be many meta-analysis studies also available, so learn from them and then accordingly develop these guidelines which should be circulated and disseminated to the level of PHCs.

**When we talk about prevention, we understand that there are certain statutory, and regulatory authorities as well, and certain under Factories Act as well there are certain guidelines laid out but a common drawback of these we see is the smaller units, which goes sometimes beyond the preview of the Factories Act, what we also call as unorganized sectors, so any suggestions for what can be done for these unorganized sectors, how can they be looped into the arms of the law and how can they be mandated to implement such silicosis control or prevention activities?**

See the factories act itself has provisions wherein like Factories Act say 20 persons without electricity and 10 persons with electricity and all that, these are the places where the Factories Acts are applicable. But in hazardous cases or hazardous occupations even these requirements can also be diluted further or they need not be fulfilled and the chief inspector of factories has a right to declare any industry, even if it is an unorganized industry, even if it is the small industry to be in the purview of Factories Act. So they will not be immune to the acts, again there are two things, the law has to reach there, and now what we have seen is there are a lot of challenges with the intention and integrity of the implementation agencies. So, I mean they would typically have focused on where there is more noise, where there is likely to be questions from the top, so that kind of a thing. For small places and all, they do come in their purview, their purview or their jurisdiction is not as small as we presume, it is much more. See even if it’s a small mine, open cast mine, there will be people working there, which comes under the purview of the Factories Act, so if they want they can reach there. Then even in the large organization or even in the medium organization, they are not seen to be focusing so much as they should do for all these reasons that’s why I talked about ethics and integrity. Another thing is it is very tough to implement the provisions of the Factories Act, the implementing agency has very few qualified medical personnel, they are made mostly of engineers and which is right because there is plant machinery, plant processes, and all that. So they should also have some basic knowledge or orientation to occupational health and especially respiratory diseases or such common diseases, which are not there currently. Even under the Factories Act, there are certified surgeons, now NIOH Ahmedabad is situated in the state of Gujarat, so in Gujarat whatever certifying surgeons are there, most of them have not done even their AFIH, they are deputed, they are on deputation from health department so they are just MBBS doctors with no exposure to occupational health, no knowledge in occupational health, no qualifications in occupational health, so in the capacity building we have to ensure, see occupational health we are saying this three months AFIH course, it’s not a big deal, it’s not something which is a post-graduate course or expert and all that, in three months we do not produce any experts, you run that course in your department so you know that, in three months only sensitization of the general practitioner can be done, not more than that. So, therefore, such courses should be conducted and all primary health care doctors should undergo this course. Unfortunately, the MBBS curriculum doesn’t have much of this, there is a very less focus and very less coverage of occupational health there, but we will have to revise that and we will have to see that.

**Is there anything else you would like to say that you feel you were not able to say during the interview?**

There has to be mass awareness about occupational health, see currently what we see is that health is seen as welfare, in most industries health is seen as welfare activity, wherein they have to compulsorily or necessarily spend some money and not expect any returns on that money, therefore, spent as less as possible. We also see in many industries, therefore, the, I mean there is a difference in focus which they have on their main activities and the medical activities in their organization, so they tend to outsource the medical activities, so wherein some doctors will come, they will keep changing and there is no understanding of what is happening inside the plant. Therefore, the focus on occupational health has to be increased across industries, awareness has to be increased, and enforcement also has to be increased.
