# Supplementary material for: A mixed-methods study on impact of silicosis on tuberculosis treatment outcomes and need for TB-silicosis collaborative activities in India
Source: Sci Rep. 2023 Feb 16;13:2785. doi: 10.1038/s41598-023-30012-4 (PMC9935606; doi:10.1038/s41598-023-30012-4)
Supplement: Supplementary file 4 — Supplementary Information 4. [file 41598_2023_30012_MOESM4_ESM.doc]

**Supplementary Table 4: Description of codes on the need for collaborative TB-silicosis activities as perceived by experts during April-July 2022**

| **Categories** | **Codes** | **Description** |
| --- | --- | --- |
| Programmatic need | Dual burden | Experts perceived that silicosis and tuberculosis have a dual burden for India and need to be addressed together. |
|  | Higher default | Due to persistent symptoms, patients tend to stop the medicines and default on the treatment regimen. |
|  | Higher death | Due to dual diseases, the complications among patients with silico-tuberculosis are higher. As per the experts, this also led to higher death rates. |
|  | Higher treatment failure | Patients with silico-tuberculosis demonstrate less sputum conversion. Also, experts believed that silico-tuberculosis patients require a longer duration of treatment to achieve sputum conversion. These reasons led to higher treatment failure. |
|  | Higher drug resistance | It was observed by the experts that drug resistance was higher among patients with silico-tuberculosis. |
|  | Early detection | Collaborative TB-silicosis activities would help in the early detection of the disease. X-rays of patients can be shared with physicians for early detection of either disease. |
|  | Diagnostic difficulty | The clinical features and X-ray pictures for silicosis and TB are the same. Therefore, doctors face diagnostic difficulties in differentiating the two diseases. |
|  | Prevent spread of infection | Unless we diagnose cases of TB early, we would not be able to prevent the spread of infection. |
|  | Tuberculosis elimination | India is targeting the elimination of TB by the year 2025. As exposure to silica dust makes people susceptible to TB, experts perceived that it would be necessary to address the rising number of cases of silicosis to keep a check on the number of cases of TB. |
|  | No treatment for silicosis | TB, being an infection, can be treated. However, silicosis, once affected, cannot be reversed. Further, there is no treatment for silicosis. |
|  | No silicosis program | No nationwide program or policy exists for the control/ elimination of silicosis. |
| Client need | Low-income population | People working in industries where silica dust is generated belong to low-income populations and thus, it becomes imperative to detect such cases early. |
|  | Limited job opportunities | People working in the silica dust generating industries do not have the skills to take up any other job. Thereby, they are stuck with the same industries. Further, if the sole earning member of the family gets afflicted with silicosis, it becomes difficult for the family to survive. Thus, early detection would be helpful. |
|  | Occupational history not elicited | Sometimes even doctors are not eliciting occupational history. When the patient presents with TB-like symptoms, doctors just send their sputum and start treatment without asking for any occupational history. |
|  | Increased risk of TB | The experts noted a higher prevalence of TB in areas with industries producing silica dust. Also, silica dust led to an immunocompromised state and increased the susceptibility of TB. |
|  | Worsened prognosis | Both diseases complement each other in damaging the lungs. The prognosis is much more severe when both conditions co-exist. |

TB: tuberculosis

**Supplementary Table 5: Description of codes on the implementation mechanisms for collaborative TB-silicosis activities as perceived by experts during April-July 2022**

| **Categories** | | **Codes** | **Description** |
| --- | --- | --- | --- |
| Diagnostic strategy | | Investigate all silicosis for TB | Silica dust exposure leads to an immunocompromised state and increases susceptibility to the development of TB. Apart from this, silicosis itself predisposes the patients to develop TB. Thus, the experts suggested that all patients with silicosis should be tested for TB. |
|  |  | Investigate treatment failure for silicosis | Experts felt that a large number of drug-resistant cases may have been associated with silicosis, and would be contributing to treatment failures. Thus, they suggested investigating all patients with TB with the treatment outcome of ‘treatment failure’ for silicosis. |
|  |  | Investigate all TB with occupational history for silicosis | Experts perceived that all patients with TB with an occupational history of exposure to dust in industries should be screened for silicosis. They believed that occupational history for those people working in silica-dust-exposed industries cannot be missed if a proper history is taken. Therefore, it would not be difficult to identify the patients with TB for screening for silicosis. |
|  |  | Bidirectional screening in high silicosis-burden areas | In areas with a high silicosis burden such as industrial pockets or zones, there are higher chances of early detection of silicosis as well as TB. As per the experts, there are identified pockets in each state where industries where silica dust is known to be generated. Thus, the experts felt that bidirectional screening for TB-silicosis in areas with high silicosis burden would give a better yield. |
| Diagnostic tests | TB among silicosis | Sputum microscopy | For the diagnosis of TB among patients with silicosis, the experts suggested the recommended diagnostic tests such as sputum microscopy and Cartridge-based Nucleic Acid Amplification Test (CBNAAT). Under the TB program, even if the recommended tests are negative, physicians can label the patient as suffering from TB based on symptoms. |
|  |  | Nucleic Acid Amplification Test |  |
|  |  | Clinical |  |
|  | Silicosis among TB | High-resolution CT | For diagnosis of silicosis among TB, a high-resolution CT scan was said to be the gold standard test, but a costly one. Generally, the experts suggested, that high-resolution CT was prescribed for differentiating silicosis from TB. They also suggested pulmonary function tests using a spirometer to monitor lung capacity. Further, the experts suggested that the chest X-ray remained the cheaper option for the diagnosis of silicosis. However, the radiological findings suggestive of silicosis should be supplemented with an occupational history of exposure to silica dust to arrive at a final diagnosis. |
|  |  | Pulmonary Function Test |  |
|  |  | Occupational history |  |
|  | Both | X-ray | Chest X-ray is useful for the diagnosis of either disease. Nowadays, digital X-rays are frequently used as they provide a better resolution. When in doubt, high-resolution CT is preferred for differentiating silicosis from TB. As mentioned earlier, experts suggested supplementing the history of exposure to silica dust with the radiological findings. |
|  |  | Digital X-ray |  |
| Program modalities | | Block-level facility | For implementing the collaborative TB-silicosis activities, the experts believed in developing in-patient facilities with the provision of X-rays and pulmonary function tests at the Community Health Centers (CHCs) located at the block level. At least CHCs in areas with the presence of silica-dust-generating industries could be developed to implement the collaborative activities. |
|  |  | Referral center | Experts also suggested having a referral center at the district level. The patients who require advanced care can be referred from the CHCs to such referral centers. |
|  |  | MoU with private | For making radiology services affordable, MoUs can be signed by the government with private providers. The people working in silica-prone industries are very poor and are not able to afford the cost of X-rays or CT scans. Thus, such MoUs would subsidize the costs of radiology. |
|  |  | Patient welfare committee | For reducing the costs of radiology investigations, the experts also suggested utilizing the patient welfare committees functional at all the government-run peripheral health centers. |
|  |  | Trained physiotherapist | Physiotherapists are available at the Community Health Centers (CHCs). However, they are not trained in the exercises which would help patients with decreased lung capacity in silicosis. Thus, the experts suggested posting a trained physiotherapist at the CHCs for providing respiratory rehabilitation to patients with silicosis. |
| Workplace interventions | | Digital X-ray van | For early diagnosis of silicosis at the workplace, the experts suggested using a digital X-ray van. Such a van is currently functional in the Khambhat block of Anand district in Gujarat. |
|  |  | Sputum collection at workplace | For early diagnosis of TB at the workplace, it was suggested to collect sputum from the people at the workplace. The collected sputum can be sent to a Designated Microscopy Center (generally at a Primary Health Center) for testing. Reports can be delivered at the workplace and patients can be further managed based on the sputum results. |

CBNAAT: Cartridge-based Nucleic Acid Amplification Test; CHC: Community Health Center; CT: Computer Tomography; MoU: Memorandum of Understanding; TB: tuberculosis
