# Supplementary material for: A mixed-methods study on impact of silicosis on tuberculosis treatment outcomes and need for TB-silicosis collaborative activities in India
Source: Sci Rep. 2023 Feb 16;13:2785. doi: 10.1038/s41598-023-30012-4 (PMC9935606; doi:10.1038/s41598-023-30012-4)
Supplement: Supplementary file 5 — Supplementary Information 5. [file 41598_2023_30012_MOESM5_ESM.doc]

**Supplementary Table 6: Description of codes on the reasons for unfavorable treatment outcomes among patients with silico-tuberculosis as perceived by experts during April-July 2022**

| **Categories** | **Codes** | **Description** |
| --- | --- | --- |
| Patient factors | Persistent symptoms | As silicosis is not cured, the symptoms of the patients persist even after the treatment with TB. This makes the patients think that medicines are not effective and they stop treatment. |
|  | Seeking symptomatic relief | A patient starts feeling better after one or two months of the intensive phase of the treatment regimen for TB. As they feel symptomatically good, they stop their treatment. |
|  | Self-medication | As the symptoms of TB and silicosis are the same, people working in factories think that they are suffering from recurrent TB. Once they are conversant with the medications, on developing similar symptoms again, the patients tend to self-medicate themselves and their colleagues. |
|  | Adverse drug reactions | People with silico-tuberculosis stop anti-TB medicines if they experience adverse drug reactions. |
|  | Lack of medicine adherence | Due to odd and long working hours, persistent symptoms, and due to migration, people with silico-tuberculosis find it difficult to comply with the anti-TB regimen. |
|  | Lack of time | Workers are most often busy with their work. Moreover, employers do not give them a break to visit the hospital. Thus, their primary goal is to work rather than take care of their health. |
|  | Lack of awareness | Patients do not suspect a problem with their health and think that the diseases cannot occur to them due to a lack of awareness. Also, they are not able to seek appropriate guidance due to the lack of awareness. |
|  | Lack of PPE use | People working in factories do not use PPE. Due to lower education levels and a lack of awareness, they do not use PPE. |
|  | Lack of alternative job | As people working in the silica-dust industries are usually daily wagers, they find it difficult to search for an alternative job. Even experts who have worked with agate (stone bead) workers found it difficult to find alternate occupations for them. |
|  | Lack of access to healthcare | Even when patients develop symptoms or adverse drug reactions, due to the remote location of certain secondary or tertiary-level healthcare facilities, they might find it difficult to access appropriate healthcare. |
|  | Lack of appropriate healthcare-seeking | Experts felt that the workers were not visiting a Primary Health Center in case of any health-related complaints. The people working in factories generally consult a non-allopathic doctor or visit a medical store for their illnesses. Thus, they tend to self-medicate themselves, and the chances of developing drug resistance increase. |
|  | Delayed healthcare seeking | The working population sought healthcare only when the disease has progressed. A timely visit to Primary Health Centers was not observed by the experts. |
|  | Concomitant tobacco/ alcohol use | Due to the long working hours, many workers abuse tobacco and alcohol to keep themselves awake and attentive. The use of such addictive substances also increases the chances of unfavorable treatment outcomes for TB. |
|  | Return to dusty work environments | Once the patients start feeling better during the initial intensive phase of the anti-TB treatment regimen, or, after receiving initial symptomatic relief, the workers return to dusty work environments which increases their exposure to silica dust even further. The vicious cycle continues and they again develop TB. |
|  | Migrant daily wage earners | Once they are known to be suffering from silicosis or silico-tuberculosis, they should be shifted to an alternative occupation. But, people working in silica-dust generating factories are migrant daily wage earners. They have limited job opportunities. They tend to return to dusty work environments. |
|  | Fear of getting laid off | Due to limited job opportunities, the workers fear that if they develop TB or silicosis, they would be asked to leave their job. Due to this fear of getting laid off, they do not undergo a medical examination. |
| Disease factors | Alveolar block | The alveoli of the lungs get blocked due to silica dust. The silica dust in alveoli cannot be removed by any means, as per the experts. |
|  | Lack of gas exchange | Due to alveolar block, gas exchange cannot take place and the patient progresses towards respiratory failure. |
|  | Decreased lung capacity | Experts felt that due to decreased lung capacity, patients with silico-tuberculosis had unfavorable TB treatment outcomes. |
|  | Presence of comorbidities | Comorbidities like diabetes and HIV increase the probability of unfavorable TB treatment outcomes among patients with silico-tuberculosis. Comorbidities result in lesser sputum conversion rates. |
|  | Dormant tuberculosis bacilli | TB bacilli remain dormant in the lungs, which gets activated among patients with silico-tuberculosis from time to time and causes relapse of TB. |
|  | Dual disease burden | As the patients are suffering from two lung diseases, the effect is doubled. As per the experts, both the diseases - silicosis and TB - complement each other. When we treat TB, it is only one disease that is cured, the silicotic component continues even after cessation of dust exposure. Due to the dual disease burden, the prognosis is much worse as compared to TB alone. |
|  | Macrophage dysfunction | The experts explained that the macrophages primarily work to remove the silica dust particles. As the macrophages are already overwhelmed in removing silica dust particles, when the patient is exposed to the TB bacilli, the macrophages fail to clear the TB bacilli effectively. This phenomenon makes patients with silico-tuberculosis more susceptible to complications with higher chances of unfavorable TB treatment outcomes. |
|  | Dense fibrosis | Patients with silico-tuberculosis ultimately develop progressive massive fibrosis which gets complicated due to the invasion of the TB bacilli, increasing the chances of death among such patients. |
|  | Drugs unable to reach lung tissue | Due to fibrosis of the lung tissue, the blood supply is reduced. Among patients with silico-tuberculosis, due to the lack of blood supply to the lung tissue affected by fibrosis, the drug is unable to penetrate the tissue thereby reaching lower concentrations. Due to the lower concentration of drugs, they are not effective. As the drugs are rendered ineffective, treatment failure is higher among patients with silico-tuberculosis. |
|  | Non-tuberculous mycobacteria | Although non-pathogenic, patients with silicosis often demonstrate the presence of non-tuberculous mycobacteria (e.g. mycobacterium kansasii or avium) along with the tuberculosis bacilli. The presence of non-tuberculous mycobacteria increases the chances of drug resistance as they are resistant to many drugs. |
|  | Drug resistance | The presence of quartz (silica dust) in the lungs also leads to the emergence of drug resistance among patients with silico-tuberculosis. |
|  | Recurrent pneumothorax | As per the experts, recurrent pneumothorax is a terminal complication among patients with silicosis, which leads to death. Patients with silico-tuberculosis have a greater tendency of developing recurrent pneumothorax and thereby have higher chances of death. |
|  | Delayed diagnosis | Most of the people working in the silica-dust-generating factories are migrant workers who tend to seek care when the disease has progressed. A lack of awareness also contributes to delayed diagnosis. Also, symptoms of silicosis are presumed to be that of TB. |
|  | Misdiagnosis | Silicosis is most often misdiagnosed as TB. Due to a lack of response to treatment and lack of awareness, even silico-tuberculosis is misdiagnosed as multi-drug resistant TB. |
| Workplace factors | Lack of employer support | Employers do not allow time for workers to consult a doctor or visit a health facility in case of illness. |
|  | Silica dust exposure | Silica dust exposure was said to be one of the reasons for unfavorable treatment outcomes. After initial symptomatic relief, workers return to their jobs where they are exposed to silica dust again. The vicious cycle continues as they develop TB again and their silicosis worsens. |
|  | Workplace spread of infection | Due to the lack of use of PPE or lack of cough etiquette, the TB infection keeps on spreading in the workplace. |

PPE: Personal Protective Equipment; TB: Tuberculosis

**Supplementary Table 7: Description of codes on the solutions for unfavorable treatment outcomes among patients with silico-tuberculosis as perceived by experts during April-July 2022**

| **Categories** | | **Codes** | **Description** |
| --- | --- | --- | --- |
| Improve health systems | | Early diagnosis | As there is no cure for silicosis, early diagnosis is important to prevent silicosis. Early diagnosis will enable us to remove the patient from further exposure to silica dust and thereby increase the survival of the patient. |
|  |  | Symptomatic relief | Due to the lack of a definitive cure for silicosis, experts felt the need for symptomatic relief to improve their physical quality of life and provide them with a comfortable life. |
|  |  | Health check-ups | Health check-ups would help in early diagnosis and increase the survival of the workers. Such health check-ups should be linked with the nearest TB center. The health department can coordinate with industries for such health check-ups. |
|  |  | In-patient treatment | Patients with silico-tuberculosis should be provided indoor treatment at government health facilities. Such government hospitals should be equipped with facilities such as pulmonary function tests, X-rays, oxygen therapy, bronchodilators, steroids, and other supportive treatment. |
|  |  | Early rehabilitation | Vocational and pulmonary rehabilitation should both be done together and timely. Pulmonary rehabilitation should be done to make the patient comfortable and thereby vocational rehabilitation should be done to remove the patient from further exposure to silica dust. Early detection will also help in early rehabilitation and the use of suitable personal protective equipment. |
|  |  | Mobile van | Experts suggested using a mobile van for providing timely services to the patients such as for addressing adverse drug reactions. It was also suggested to use a mobile van for diagnostic procedures in areas with a high silicosis burden. |
|  |  | Tracking migrant workers | As the workers are migrating from one place to another, it was suggested to track them. The workers change their job and continue to get exposed to silica dust. Tracking the workers will help in establishing a robust surveillance system and monitoring the continuum of care. |
|  |  | Health card | Patients with silico-tuberculosis must be given a health card with all information about their investigation, ongoing treatment, and dates of follow-up visits. |
|  |  | Develop cost-effective screening test | A cost-effective screening test for silicosis, such as a serological test, was needed for early diagnosis. Experts felt that even if a test would help to shortlist some people, they could be then investigated in detail. |
| Updates in TB program | | Differential TB care | Differential TB care is a guideline under the National TB Elimination Program (NTEP) wherein patients at high risk of unfavorable TB treatment outcomes are identified early and relevant interventions are provided to avoid unfavorable outcomes. Experts perceived the need to advocate for including silicosis as one of the important risk factors for unfavorable TB treatment outcomes under the differentiated TB care model. |
|  |  | Differential silicosis care | Experts felt the need to implement differential silicosis care guidelines similar to the differential TB care model. Patients with silicosis should be screened and treated for other underlying diseases. Experts suggested including certain criteria like oxygen level, pulmonary function test, breathlessness, or any other symptomatic criteria in differential silicosis care. Experts also suggested integrating the differential silicosis care model with the NCD program and referring patients with specific risk factors to appropriate higher centers for management. |
|  |  | Occupational history in TB program | The occupational history of exposure to silica dust should be included in the TB program and those found to have a history should be screened for silicosis. This would help in curtailing further exposure to silica dust for the workers. |
|  |  | Comprehensive TB care package | Patients with silico-tuberculosis should be extended all the support in terms of nutrition, treatment of opportunistic infections, cash assistance, regular follow-up, and other investigations to improve their treatment outcomes. People working in the silica-dust-generating industries are generally migrant workers making it difficult to track them. Suitable mechanisms were needed, as per the experts, to provide a comprehensive TB care package to all patients with silico-tuberculosis. |
|  |  | Prophylactic anti-TB drugs | Experts suggested exploring administering anti-TB drugs prophylactically to workers exposed to silica dust for the prevention of TB. |
|  |  | Longer treatment | Experts believed that patients with silico-tuberculosis should be treated for a longer duration instead of the conventional duration under the TB program. Longer time for drugs to reach the site and higher chances of drug resistance among patients with silico-tuberculosis were the reasons provided by the experts for recommending a longer treatment of TB. |
|  |  | Closer and longer follow-up | Patients with silico-tuberculosis display more symptoms than patients with TB alone. Due to persistent symptoms, patients with silico-tuberculosis tend to flip from one doctor to another. A closer and longer follow-up are therefore required for patients with silico-tuberculosis. |
|  |  | Counseling on persistent silicosis | Patients with silico-tuberculosis need to be counseled that since silicosis cannot be cured, they would continue to have symptoms of silicosis. Only the TB disease would be cured with the regular intake of anti-TB medicines. |
|  |  | Integrated surveillance | To track the patients with silico-tuberculosis, a robust surveillance system is needed with integration of all diseases including silicosis and TB. To start with, the experts suggested integrating occupational health surveillance with the National TB Elimination Program (NTEP). |
|  |  | Inter-sectoral coordination | Inter-sectoral coordination between the departments of labor and health is required for better delivery of services for patients with silico-tuberculosis. |
|  |  | Training for Medical Officers | Many patients with silico-tuberculosis are misdiagnosed as patients with drug-resistant TB. Thus, training and awareness of diagnosis and management of silico-tuberculosis are required for medical officers. Further, experts suggested that those medical officers who are posted/deputed to serve in the department of labor should be specifically trained in the diagnosis of silicosis. |
|  |  | Bidirectional activities | Experts suggested that bidirectional activities between silicosis and TB were successfully implemented in the state of Rajasthan with well-established linkages between the two programs. Experts believed that not only in India but also in the international forum it has been suggested to implement bidirectional activities between silicosis and TB for early detection and effective treatment. |
|  |  | Online registry with teleconsultation | In the state of Rajasthan, the government has implemented an online system of registration for workers with exposure to silica dust. The medical officers at the Primary Health Center and Community Health Center can upload the chest X-rays of workers online for comments from experts on diagnosis through teleconsultation. If TB is ruled out and if experts suggest, then medical officers prescribe a high-resolution CT scan and seek advice from the experts. |
|  |  | Employer as treatment supporter | Experts suggested employers be nominated as treatment supporters under the NTEP program for workers who are diagnosed with TB. Treatment supporters replenish the medicines of patients with TB every month and monitor their treatment adherence. As per the experts, appointing employers as treatment supporters would improve TB treatment outcomes. |
| Patient factors | | Improve healthcare seeking | Experts suggested that workers should be motivated to go for medical check-ups at government healthcare facilities to enable early diagnosis and treatment. |
|  |  | Awareness on disease | It was felt important to generate awareness of silicosis and silico-tuberculosis and the severity associated with these diseases among workers working in silica-dust-generating industries. By creating awareness, workers would easily suspect the chances of suffering from such diseases and would seek care. Timely care-seeking would ensure early detection of cases. Also, more the awareness, the healthier the workforce. |
|  |  | Financial assistance | Some monetary assistance while the workers have suffered from silicosis would help them to avail better healthcare. |
|  |  | Pension schemes | Most of the workers who are diagnosed with silicosis would not be able to continue to work for a longer time. Thus, a pension scheme would ensure some financial support to the silicosis-affected household. |
| Employer interventions | | Stop further dust exposure | The key to improving the treatment outcomes of patients with silico-tuberculosis lies with prevention rather than the treatment of diseases, the experts felt. Once silicosis is diagnosed, stopping any further dust exposure was said to be the most important intervention suggested by the experts. Early diagnosis coupled with the removal of the worker from dust exposure was needed. The worker needs to be offered an alternate occupation where he is no longer exposed to silica dust. Failing to stop the exposure leads to repeated treatment for TB. |
|  |  | Employer support | Owners of industries/factories should try providing financial support to the workers suffering from silico-tuberculosis. |
|  |  | Provision of sick leave | Experts felt that employers should allow a leave of around 15 days without a cut in the salary for their employees suffering from silicosis to allow them proper rest. |
|  |  | Employer awareness generation | Creating awareness about the diseases among employers was also felt to be important. Many workers affected by silicosis work in unorganized sectors. Thus, supervisors of such workers should also be made aware of the disease. Awareness should be created on how silicosis develops due to silica dust exposure and how it can lead to the development of TB. This awareness generation would help in the early treatment of the workers. |
| Adverse drug reactions | | Awareness on ADR | Even when the workers are on treatment regimens for TB, symptoms such as hemoptysis and breathlessness might continue due to the silicosis component of the disease. The workers might perceive these symptoms as being caused due to the anti-TB drugs. Also, the treatment supporters would not be available in all areas where the workers suffering from TB are residing. Thus, the experts felt that it was very important to generate awareness among the workers on the common adverse drug reactions and counseling on not to stop the drugs but to seek care. The workers need to be informed about the benefits of completing the full course of treatment in the prescribed months under the NTEP. |
|  |  | Monitor ADR | The adverse drug reactions of the workers need to be monitored by peripheral health staff. The health staff should regularly visit the workers for treatment for TB and act as a link between the worker and the medical officer. The health staff can deliver the medicines prescribed by the medical officer for managing the adverse drug reactions, which would help them to complete the treatment. |
|  |  | Managing ADR | Adverse drug reactions are mostly treated symptomatically. The experts suggested that symptomatic medicines should be made available to workers who experience adverse drug reactions. As per the experts, patients are compliant with the treatment, however, become non-compliant when their adverse drug reactions are not addressed properly. |
|  |  | Frontline worker involvement | For managing the adverse drug reactions, the experts opined to train peripheral health workers like accredited social health activists (ASHA), multi-purpose health workers (MPHW), and female health workers (FHW) on the simple medicines required for managing the ADR. |
| Alternative therapies | | Physiotherapy | Experts who had experience in the state of Rajasthan suggested incorporating physiotherapy exercises for patients with silicosis for any possible additional relief from the symptoms. |
|  |  | Oxygen therapy | Portable oxygen cylinders have been used for patients with silicosis in Rajasthan and have given positive results to the experts. |
| Silicosis prevention and control | Surveillance | Silicosis surveillance | A surveillance system needs to be established for tracking all patients with silicosis. The experts believed that similar to the surveillance for TB, a routine surveillance mechanism needs to be established for silicosis. A surveillance system would be helpful in the early detection of cases of silicosis. |
|  |  | Camp-based surveillance | Experts also suggested arranging diagnostic camps in industries where silica dust is generated. Such camps would also help in the early detection of cases of silicosis. |
|  |  | Active case finding | Active search for cases through a survey in the field would help in the detection of cases of silicosis. Active case finding is already being used for TB, the experts suggested expanding it to include silicosis as well. |
|  |  | Mapping areas | Identifying and mapping areas where industries generating silica dust are located in Gujarat as well as in the rest of India would be an initial first step towards eliminating the diseases. Relevant districts and sub-district areas/clusters should first be identified to prioritize them for tracking. |
|  |  | Periodic examination | Periodic medical examination of workers exposed to silica dust should be mandatorily carried out. It is a statutory requirement to be repeated every six months and should be followed by all factories. Apart from routine medical check-ups, X-rays and sputum should be a part of such examinations. |
|  |  | Tracking unorganized sectors | After mapping the areas/clusters having factories where silica dust is being generated, it was felt important to track these factories. The experts believed that most of such factories would be belonging to the unorganized sector and that most of the cases of silicosis were being reported from the unorganized sector. Thus, tracking would help to focus on them for implementing control activities. |
|  | Programmatic guidelines | Diagnostic algorithm | Experts opined on the lack of a standardized diagnostic algorithm for silicosis. Also, the lack of standardized guidelines on the certification of silicosis was highlighted. Diagnostic algorithms need to be developed and circulated widely. |
|  |  | Management guidelines | Standardized guidelines on preventive treatment, symptomatic management, and treatment options should be developed and circulated widely. |
|  |  | Rehabilitation guidelines | Although rehabilitation mechanisms such as physiotherapy, oxygen therapy, change in occupation, etc. were suggested by the experts, they felt that such guidelines should be a part of a standardized document. |
|  |  | Silicosis elimination program | A silicosis elimination program encompassing diagnostic criteria, preventive treatment, symptomatic management, prevention of silica dust exposure, awareness generation, treatment, and rehabilitation was felt important for wider implementation. Program managers are clueless on several aspects even after a patient with silicosis is diagnosed. A separate program with reporting and review/monitoring would entail funding of the program activities under a separate budget head. |
|  |  | Pneumoconiosis policy | A policy on the detection, prevention, relief, and rehabilitation of pneumoconiosis is in place in Rajasthan. An expert from Rajasthan suggested scaling up this policy for the entire country. The expert believed that silicosis is not a problem that is restricted to certain states, it is a nationwide problem. There are other sectors such as coal mines in many other states. Therefore, it was suggested to design and implement a nationwide pneumoconiosis policy focusing not only on silicosis but also on other industrial dust-related respiratory diseases. |
|  | Preventing silica dust exposure | Alternative uses of silica dust | Experts suggested exploring alternative uses of silica dust being generated in the industries. A few examples given by the experts were the preparation of bricks, tiles, construction blocks, ceramic cups, and ceramic saucers, from the silica dust generated. |
|  |  | Silica dust disposal | In Khambhat, currently, due to the use of the water spraying system, the silica dust was getting settled on the ground as wet dust. However, when the wet dust dried, it was again used to generate dry silica dust. Thus, the experts suggested disposing of the wet dust through a system of deep burial or covering it with soil. |
|  |  | Subsidy for silica dust disposal | Disposal of silica dust was perceived to be important. Thus, it was suggested that government should provide subsidies to employers for establishing silica dust disposal systems. |
|  |  | Exhaust system | An exhaust system was experimented with by some experts for capturing silica dust and reducing exposure to the workers. The experts also suggested installing exhaust fans at places in the factories where silica dust was generated. Experts suggested that the use of exhaust systems should be explored in factories where silica dust was generated. |
|  |  | Water spraying system | Also called a wet process, many factories/cottage industries are using the water spraying system. In this system, a continuous drip of water droplets is poured exactly on the site where the polishing of stones takes place. The water drops settle some of the silica dust which is generated during the polishing of agate stones. As per the experts, this system is in use at Khambhat as well as at some places in Rajasthan. |
|  |  | Multi-sectoral coordination | To tackle the problem of silicosis, the experts suggested establishing coordination between various departments such as health, labor and employment, and the environment. The district collector (administrative head of a district in India) should facilitate such coordination among various departments for the prevention and control of silicosis. |
|  |  | Dedicated place for all workers | In Khambhat block, many agate stone polishers are working at their homes in the cottage industry. Experts suggested having an industrial estate near their homes where all workers can work together. As all workers would be at one dedicated place, it would simplify the process of registration of workers and conducting periodic medical examinations. |
|  |  | PPE | Masks, goggles, and hand gloves were the personal protective equipment suggested for reducing the exposure to silica dust among the workers. |
|  |  | Dust reduction at source | Reduction of dust at the source to the permissible level through elimination, substitution, engineering control, administrative control, and lastly the use of personal protective equipment was suggested by the experts. The experts reiterated that this should be the hierarchy of control measures in the factories. Industrial hygiene measures for the reduction of silica dust at the source and in its path in the air before it reaches the worker are of paramount importance. |
|  |  | Alternative job | To stop further dust exposure, the workers need to be moved to an alternative job. This alternative job within the same factory should be in an area where there is no silica dust being generated. Experts believed that it was easier to say but difficult to put into practice. However, the experts felt that it was the role of government regulatory agencies to make sure that the factory owners shift the workers to an alternative job based on the symptoms they develop. The experts also said that it was important to not remove the person from his job as this will again lead to undernutrition and cause other diseases. So, such a job change should ensure that their source of livelihood remains intact. |
|  |  | Substitution of hazardous material | Substituting from a more hazardous material (for example, stones) to less hazardous types of material was suggested. As per the experts, such substitution was one of the methods of reducing dust exposure of workers to harmful forms of silica dust. |
|  |  | Enclosed workspaces | Places, where the silica dust is being generated at the factories, should be enclosed so that only a limited number of workers are exposed to the dust. Also, the experts suggested having only a limited number of workers in areas in the factories where dust was being generated. |
|  |  | Widespread prevention activities | Experts felt that the workers working in industries do not understand the seriousness of silicosis as a disease. Many workers suffer from silicosis within 5-10 years of exposure and die within 15 years of exposure. Once the disease develops, there is not much that can be done due to the lack of a cure for silicosis, the experts felt. So, the experts recommended widespread prevention activities. They also suggested that a part of the budget allocated for rehabilitation activities should be spared for prevention activities. |
|  | Law enforcement | Law enforcement for smaller units | Experts felt that it was the smaller units from where most of the cases of silicosis were being reported. The smaller units were also the ones flouting the statutory norms laid down under the Factories Act and the Mines Act. Thus, the experts suggested assigning accountability among the statutory agencies to implement the provisions laid down under the Factories Act, the Mines Act, and the BOCW (Building and Other Construction Workers) Act at the smaller units. |
|  |  | Ethical law enforcement | Experts agreed that the authorities can declare any unit, however small, to be under the purview of the Factories Act. But, the experts flagged concerns regarding the law reaching the smaller units, largely called the unorganized sector. Experts opined that there were a lot of challenges surrounding the intention and integrity of the implementing agencies. The experts also raised concerns regarding the outsourcing of medical examinations of the workers to doctors who are not having an understanding of occupational health. |
|  |  | Qualified personnel for law enforcement | The need for posting qualified personnel in the implementing agency was highlighted by the experts. The implementing agency has very few qualified medical personnel to implement the provisions of the Factories Act. As per the experts, the certifying surgeons who are deputed to the implementing agency are just MBBS doctors with no knowledge, exposure, or qualifications in occupational health. The experts went on to explain that even the Associate Fellow in Industrial Health (AFIH) course was only a three-month course. It was not a postgraduate-level course and a three-month course would not churn out experts in occupational health. It was also suggested to add larger parts of occupational health to the MBBS curriculum. |

ADR: Adverse Drug Reactions; AFIH: Associate Fellow in Industrial Health; ASHA: Accredited Social Health Activist; BOCW: Building and Other Construction Workers; CT: Computer Tomography; FHW: Female Health Worker; NCD: Non-Communicable Disease; NTEP: National Tuberculosis Elimination Program; MBBS: Bachelor of Medicine and Bachelor of Surgery; MPHW: Multi-purpose Health Worker; TB: Tuberculosis
